# Interplay of Polar Order and Positional Order in Liquid Crystals–Observation of Re‐entrant Ferroelectric Nematic Phase

**DOI:** 10.1002/anie.202516302

**Published:** 2025-09-30

**Authors:** Grant J. Strachan, Shona J. Ramsay, Marijus Juodka, Damian Pociecha, Jadwiga Szydlowska, John M.D. Storey, Nataša Vaupotič, Rebecca Walker, Ewa Gorecka

**Affiliations:** ^1^ Faculty of Chemistry University of Warsaw Zwirki i Wigury 101 Warsaw 02‐089 Poland; ^2^ School of Natural and Computing Sciences University of Aberdeen Aberdeen Great Britain; ^3^ Faculty of Natural Sciences and Mathematics University of Maribor Maribor Slovenia; ^4^ Jozef Stefan Institute Ljubljana Slovenia

**Keywords:** Nematic, Proper ferroelectricity, Re‐entrant phenomena

## Abstract

We show that development of polar order may spontaneously destroy the lamellar structure of a liquid crystal. This results in an unusual sequence of phases, with the ferroelectric nematic phase appearing below a non‐polar smectic phase. The effect is related to unfavourable dipole interactions within the smectic layers and can be explained by Landau theory, in which the temperature‐dependent term is non‐monotonic as it is renormalised by spontaneous electric polarisation.

## Graphical abstract

In most systems the degree of order increases monotonically with decreasing temperature. Here we report that due to unfavourable dipole interactions within the smectic layers, the lamellar structure of a liquid crystal may be spontaneously destroyed by the development of polar, ferroelectric order. This leads to the re‐entrant phenomenon, in which the liquid phase (nematic) is formed on cooling from the 1D crystal phase (smectic).

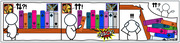

Re‐entrant behaviour refers to a situation where a system transitions from one phase to another, and upon further variation of an external parameter—typically temperature—it reverts back to the original phase. The phenomenon of re‐entrant phase behaviour is unusual, as in most systems the degree of order increases monotonically with decreasing temperature. It is driven by a competition between ordering forces (enthalpic contributions) and disordering tendencies (entropic effects). The phenomenon has been reported in very different materials, like binary mixtures with limited solubility,^[^ ^1^ ^]^ ferromagnetic systems,^[^ ^2^ ^]^ superconductors,^[^ ^3^ ^]^ etc. In liquid crystals (LCs), such competing effects may arise from dipolar interactions, steric hindrance, or molecular geometry. Re‐entrancy has been often reported in systems composed of rod‐like molecules with strongly interacting terminal groups and attributed to competing forces that favour bilayer packing in the higher‐temperature smectic phase and monolayer packing at lower temperatures, with a nematic phase obtained in a temperature range between these smectics.^[^ ^4^, ^5^, ^6^, ^7^ ^]^ The phenomenon has also been observed in materials with mesogenic cores that deviate from typical rod‐ or disc‐like shapes.^[^ ^8^, ^9^ ^]^ In these cases, packing frustration may destabilise ordered phases, leading the system to revert to a less ordered (e.g., nematic or even isotropic liquid) phase upon cooling, before going again to a more ordered structure. Other re‐entrant examples are related to the reappearance of a non‐tilted smectic phase below the tilted one,^[^ ^10^ ^]^ or ferroelectric order below the phase with antiferroelectric order.^[^ ^11^ ^]^ Here we studied re‐entrant phenomena for a new class of mesogens able to form proper ferroelectric phases. In contrast to traditional ferroelectric LCs—where chirality or bent molecular shape is essential—the proper ferroelectric systems derive their polar properties directly from the dipole–dipole interactions. LCs with proper ferroelectric order display spontaneous polarisation equalling that of crystalline materials, in combination with fluidity and varying degrees of symmetry depending on the type of LC phase. The first example of these, the ferroelectric nematic (N_F_) phase, was discovered in 2017,^[^ ^12^, ^13^, ^14^ ^]^ and since then the realm of proper polar LC phases has expanded to include orthogonal and tilted layered phases (SmA_F_,^[^ ^15^, ^16^, ^17^ ^]^ SmA_AF_^[^ ^18^ ^]^ and SmC_F_^[^ ^19^, ^20^ ^]^), heliconical phases (N_TBF_,^[^ ^21^, ^22^ ^]^ ${\mathrm{SmC}}_{P}^{H}$ ^[^ ^23^, ^24^ ^]^) and the antiferroelectric nematic phase (N_X_/M_AF_/SmZ_A_^[^ ^25^, ^26^ ^]^).

We have studied two homologous series of compounds featuring a highly fluorinated mesogenic core and thus having a strong dipole moment along the molecular long axis (∼11D): the *SR‐n‐Re* series, terminated with a dioxane unit, and the *GS‐n‐Re* series, terminated with a phenyl group. In both series, *n* stands for the number of carbon atoms in the terminal chain. Both series show similar trends—the behaviour of short homologues is dominated by a broad temperature range of nematic phases, and the longest homologues form smectic phases (Figure 1; Table S1). As the terminal chain length increases, the temperature at which lamellar order appears rises. This behaviour is typical: elongation of the terminal chain increases the tendency for self‐segregation between alkyl chains and mesogenic cores, promoting the formation of a lamellar structure. However, for the studied materials the lamellar structure appears for longer homologues than in other series of similar mesogens, suggesting that the propensity for lamellar structure is rather weak.^[^ ^21^ ^]^ Simultaneously, elongation of the terminal chain decreases the onset temperature of polar order. This is attributed to the increasing longitudinal distance between dipoles, which are mainly located in the mesogenic cores. What is exceptional for both series is that the appearance of polar order seems to weaken the lamellar structure; as a result, for intermediate homologues, specifically *SR‐6‐Re, SR‐7‐Re* and *GS‐5‐Re*, an unusual phase sequence is found with a reappearance of a nematic phase below a smectic phase. On cooling, these homologues undergo a transition from the isotropic liquid to a non‐polar nematic phase, followed by the formation of an orthogonal non‐polar smectic A (SmA) phase; a nematic phase reappears at lower temperatures, but now with ferroelectric order (reN_F_), which upon further cooling is followed by a transition to a tilted ferroelectric smectic phase (SmC_F_). It appears that, in these systems, the build‐up of polar order can destabilise the lamellar structure, giving rise to a polar nematic phase below a non‐polar smectic phase.

**Figure 1:**
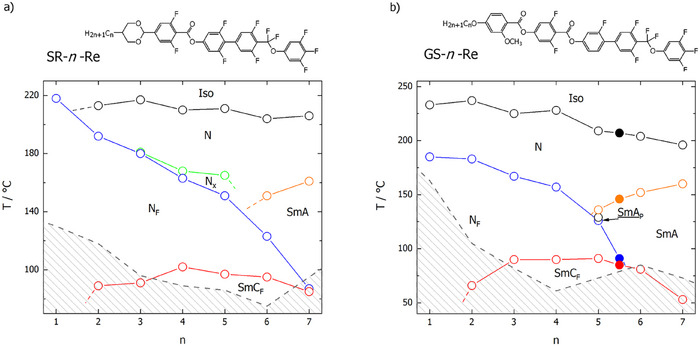
Phase diagrams for the *SR‐n‐Re* and *GS‐n‐Re* homologue series, with their general molecular structure. The transition temperatures for the mixture of homologues *GS‐5‐Re* and *GS‐6‐Re* are marked by the filled points.

The X‐ray scattering studies support the assigned phase sequences (Figures 2 and S1). For *SR‐6‐Re*, the re‐entrant N_F_ phase appears between two smectic phases; in the smectic phases the diffraction signal related to the layer thickness is of instrumental resolution. In the reN_F_ phase as well as in the N phase, it broadens, and the corresponding longitudinal correlation length deduced from the signal width is of the order of the molecular length, 2–3 nm. In the SmA phase, the position of the signal is constant and corresponds to the molecular length. At the transition to the re‐entrant nematic phase the position of this signal, which reflects the longitudinal distance between molecules, moves to higher angles. In the SmC_F_ phase the layer spacing is smaller than in the SmA phase due to the molecular tilt. In *GS‐5‐Re* the temperature range of the smectic phase separating N and reN_F_ phases is considerably narrower, and the changes of X‐ray diffraction signal width are more gradual on approaching the smectic phase from the nematic phases, above and below. Optical studies with polarised‐light optical microscopy revealed that in cells (with parallel rubbing of polymer at both surfaces) the director is perfectly orientated along the rubbing direction in the nematic phase, and the transition to the smectic phase is observed only as suppression of the Brownian motions of the director (Figures S3 and S4). At the transition to the SmC_F_ phase, the sample loses alignment, and a non‐characteristic stripy texture is formed. Optical birefringence (Δ*n*) measurements show small steps at both the N‐SmA and at the SmA‐reN_F_ phase transitions, reflecting the small increase of orientation order associated with both lamellar and polar alignment of molecules (Figure S3). In *GS‐5‐Re*, two distinct thermal events in Δ*n* versus *T* were detected (Figure S3b), preceding the reN_F_ phase, suggesting the existence of two smectic phases between the reN_F_ and N phases, the upper temperature one being a non‐polar SmA phase, while the exact nature of the lower temperature SmA phase (ferroelectric or possibly antiferroelectric) is difficult to resolve due to the narrow temperature window of the phase; thus, this phase is referred to as SmA_P_.

**Figure 2:**
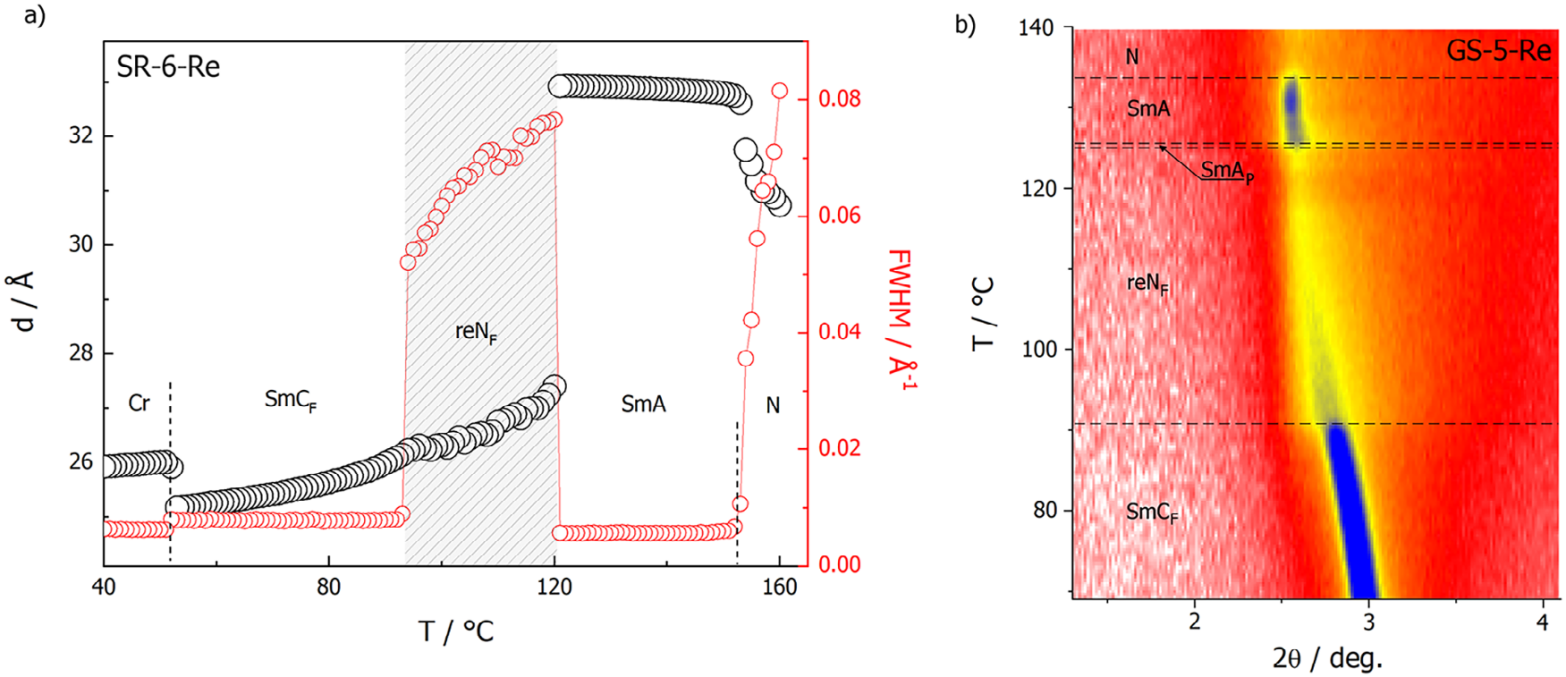
a) The layer spacing (in smectic phases) or longitudinal distance between molecules (in nematic phase) deduced from the position of the low‐angle X‐ray diffraction signal (black circles) and the full width at half maximum of the diffraction signal, reflecting the positional correlation length (red circles) for *SR‐6‐Re*, and b) the 2D plot of low‐angle diffraction signal intensity versus scattering angle and temperature for *GS‐5‐Re*.

The polar character of the re‐entrant N_F_ phase is confirmed by its response to an electric field; a clear polarisation switching current peak is observed under application of a triangular voltage. The determined value of spontaneous polarisation (Figure 3) indicates nearly complete alignment of molecular dipoles in the reN_F_ phase. In *SR‐6‐Re* the evolution of polarisation in the reN_F_ phase on approaching the SmA phase suggests discontinuous changes characteristic of a first‐order phase transition, while for its shorter homologue *SR‐4‐Re* as well as for *GS‐5‐Re* the polarisation in the N_F_ phase gradually decreases on approaching the non‐polar phase, suggesting a nearly continuous phase transition. The reN_F_ phase shows strong second harmonic generation (SHG) activity, which confirms the non‐centrosymmetric, polar nature of the phase (Figure S4). Also, a dramatic increase in the dielectric response upon entering the reN_F_ from the SmA phase is consistent with the ferroelectric character of the reN_F_ phase (Figure S5).

**Figure 3:**
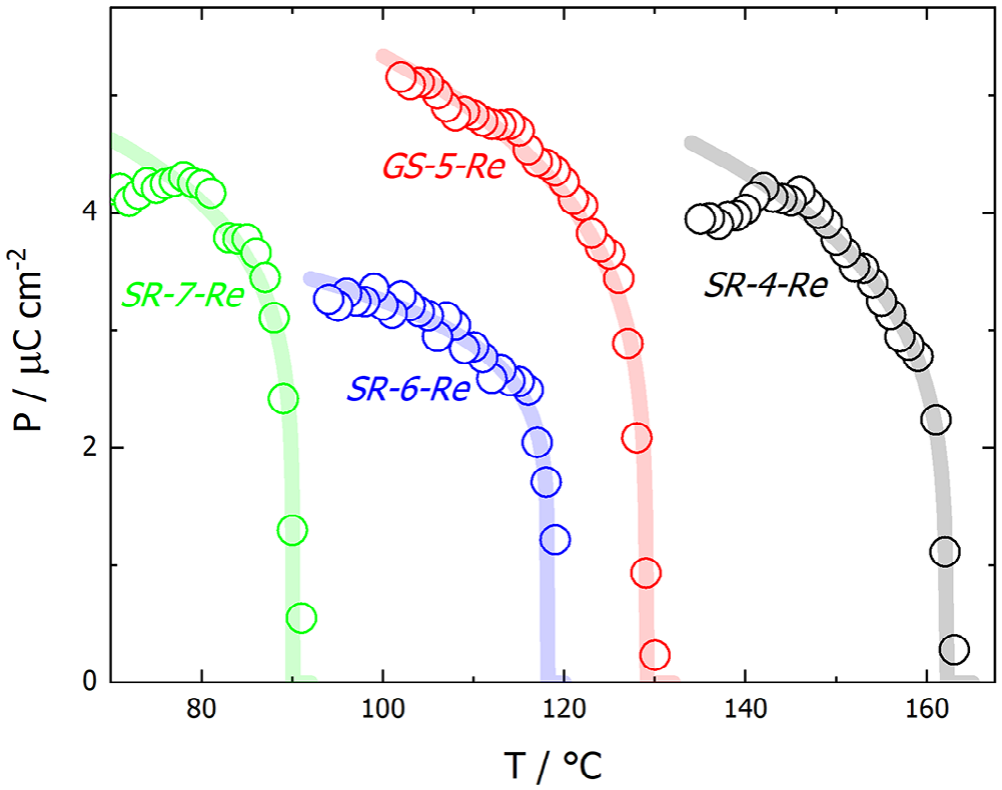
Spontaneous polarisation versus temperature for *SR‐4‐Re*, *SR‐6‐Re, SR‐7‐Re* and *GS‐5‐Re*.

Further evidence of polar order in the reN_F_ phase comes from the observation of optical textures (Figure 4). Although cells with planar anchoring show nearly ideal alignment of the director, a few air bubbles can be produced as the cell is filled with LC material. The observation of the director field around such bubbles allows us to follow the changes in the polar character of the phases. In the upper‐temperature, non‐polar nematic phase, the director is radially aligned around air bubbles, whereas in the ferroelectric reN_F_ phase, it adopts a tangential configuration. The latter is a characteristic behaviour of polar fluids that minimises surface‐bound charges. Interestingly, in *GS‐5‐Re*, an asymmetric defect pattern, tangential on one side and radial on the other, appears in the reN_F_ near the transition to the smectic phase, suggesting that the smectic phase directly above the reN_F_ phase might be antiferroelectric.

**Figure 4:**
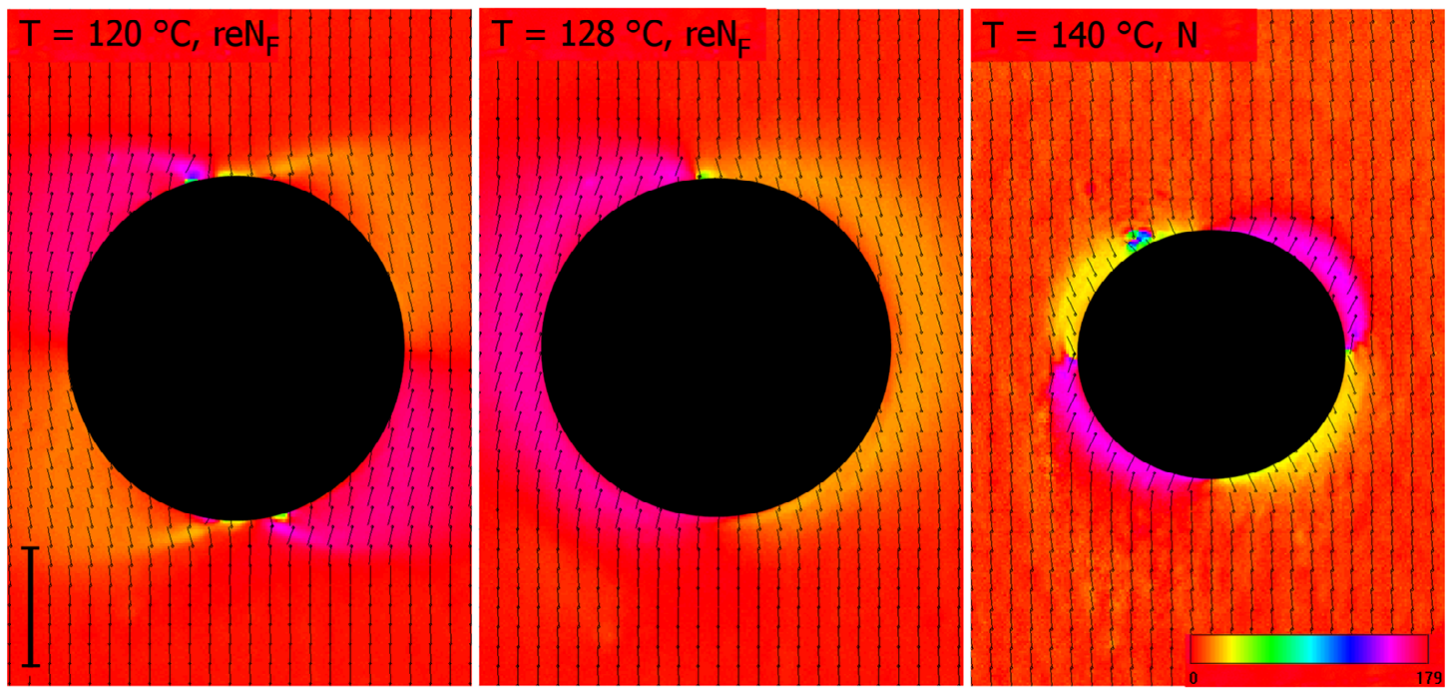
PolScope textures showing the director field in N and reN_F_ phases around an air bubble for compound *GS‐5‐Re* observed in a cell with planar anchoring and rubbing parallel on both surfaces. The colour scale codes the azimuthal orientation of the director, and a scale bar, 20 µm, is placed along the rubbing direction.

These experimental findings highlight a unique interplay between polar order and positional order in liquid crystalline phases exhibiting proper ferroelectric behaviour. We suggest that in such systems there are competing tendencies for molecular arrangement: dipole–dipole interactions favour ferroelectric order along the director, while within well‐defined smectic layers the antiparallel packing of dipoles is preferred. Thus, upon the onset of the polar phase, the system may prefer to avoid a layer structure, favouring instead a nematic phase, in which ‘transverse interactions’ between molecular dipoles are less energetically unfavourable (Figure 5a).

**Figure 5:**
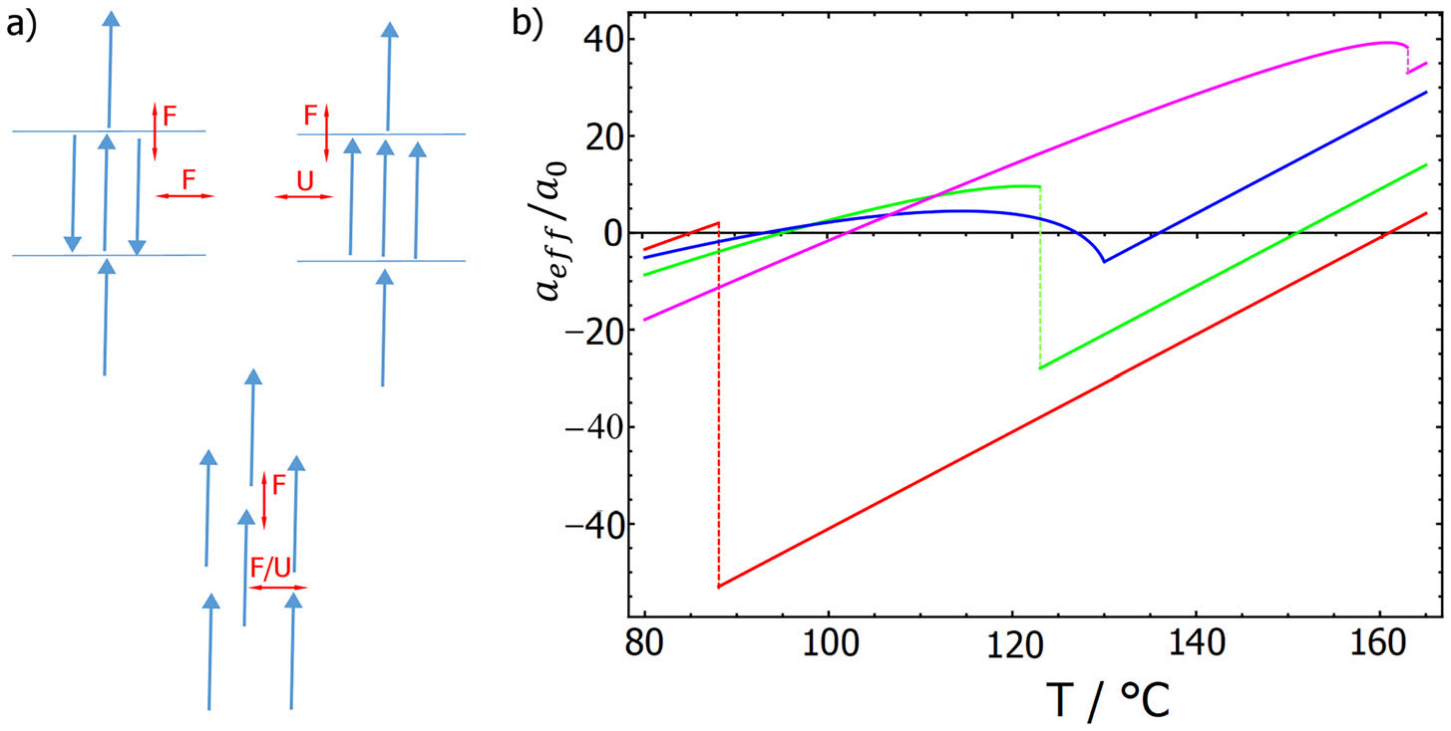
a) Considering nearest neighbour interactions of longitudinal molecular dipole moments, an antiparallel orientation of the neighbouring molecules within the smectic layer results in their favourable interactions (F), while the parallel alignment leads to unfavourable (U) interactions. Parallel arrangement of molecular dipoles in successive layers is always preferred. A displacement of the neighbouring dipoles in a nematic phase makes the transverse interactions between parallel dipoles less unfavourable. b) The temperature‐dependent Landau parameter $a_{\mathrm{eff}}$ for materials *SR‐4‐Re* (magenta), *SR‐6‐Re* (green), *SR‐7‐Re* (red), and *GS‐5‐Re* (blue). In the range in which the *a*_eff_ parameter is positive, the nematic phase becomes more stable than the smectic one.

To describe the observed phase transitions, we apply a model analogous to the Landau theory of the re‐entrant nematic–smectic A phase transition, developed by Pershan and Prost.^[^ ^27^ ^]^ Assuming a second‐order phase transition from the nematic to smectic phase, the energy density difference between the smectic and nematic phases, *f_S_*, is expressed as a power series in the smectic order parameter, ψ, as

(1) $$f_{S}=\frac{1}{2}a{\left|\psi \right|}^{2}+\frac{1}{4}b{\left|\psi \right|}^{4},$$

where a and b are Landau parameters. Parameter $a=a_{0}\left(T-T_{\mathrm{NS}}\right)$ is temperature dependent and changes sign at *T*_NS_, the transition temperature to the smectic phase. We assume that the smectic order parameter couples to polarisation (*P*) at temperatures $T<T_{P}$, where $T_{P}$ is the temperature at which polar order becomes favourable. Following Pershan and Prost,^[^ ^27^ ^]^ the simplest approach is to add an additional term of the form $g\left(P\right){\left|\psi \right|}^{2}$ to the free energy density. If a term $\frac{1}{2}g_{2}P^{2}{\left|\psi \right|}^{2}$ is added to Equation (1), the parameter a changes to $a_{\mathrm{eff}}$

(2) $$a_{\mathrm{eff}}=a_{0}\left(T-T_{\mathrm{NS}}+\frac{g_{2}}{a_{0}}P{\left(T\right)}^{2}\right).$$

With $g_{2}>0$, the transition temperature to the smectic phase will be shifted to some lower value $T_{\mathrm{PS}}$ given by

(3) $$T_{\mathrm{PS}}=T_{\mathrm{NS}}-\frac{g_{2}P{\left(T_{\mathrm{PS}}\right)}^{2}}{a_{0}}.$$

For the second‐order phase transition from the nematic to the smectic phase, the sign of $a_{\mathrm{eff}}$ defines whether the system is in the nematic ($a_{\mathrm{eff}}>0$) or smectic phase ($a_{\mathrm{eff}}<0$). At the onset of the polar order, parameter $a_{\mathrm{eff}}$ can jump from a negative to a positive value if polarisation is high enough. To have a discontinuous change in $a_{\mathrm{eff}}$, the phase transition to the polar phase needs to be first order. We can express the free energy density $f_{P}$ related to the polar order as

(4) $$f_{P}=\frac{1}{2}a_{P}P^{2}+\frac{1}{4}b_{P}P^{4}+\frac{1}{6}c_{P}P^{6},$$

where *P* is polarisation and $a_{P}$, $b_{P}$ and $c_{P}$ are Landau parameters. Parameter $a_{P}$ changes sign at temperature $T^{*}$, so we express it as $a_{P}=a_{P0}\left(T-T^{*}\right)$. For the first‐order phase transition, $b_{P}<0$ and $c_{P}$ is positive. Neglecting the term with polarisation in $a_{\mathrm{eff}}$ in the smectic free energy density, the phase transition to the polar phase is found by setting $f_{P}=0$ at P≠0 and one finds the transition temperature $T_{P}$:

(5) $$T_{P}=T^{*}+\frac{3b_{P}^{2}}{16a_{P0}c_{P}}.$$

At this temperature, polarisation, which is obtained by satisfying the condition $\delta f_{P}/\delta P=0$, is

(6) $$P^{2}\left(T_{P}\right)=\frac{3\left|b_{P}\right|}{4c_{P}}.$$

With further lowering of the temperature, polarisation increases as

(7) $$P^{2}=\frac{1}{2c_{P}}\left(\left|b_{P}\right|+\sqrt{\frac{b_{P}^{2}}{4}+4c_{P}a_{P0}\left(T_{P}-T\right)}\right).$$

At temperature $T_{P}$, the parameter $a=a_{0}\left(T_{P}-T_{\mathrm{NS}}\right)$ is negative. At the onset of polar order it jumps to the value $a_{\mathrm{eff}}\left(T_{P}\right)=a_{0}\;\left(T_{P}-T_{\mathrm{NS}}+\frac{g_{2}}{a_{0}}P{\left(T_{P}\right)}^{2}\right)$, which can be either negative or positive, depending on the magnitude of polarisation (given by Equation 6). If $a_{\mathrm{eff}}\left(T_{P}\right)>0$, then the smectic phase is not stable anymore, and one observes a phase transition to the polar nematic phase, N_F_. With further lowering of the temperature, polarisation increases as given by Equation (7); however the increase is slower than the decrease of the term $\left(T-T_{\mathrm{NS}}\right)$ in the expression of $a_{\mathrm{eff}}$, so eventually $a_{\mathrm{eff}}$ becomes negative, allowing transition back to the smectic phase.

If $a_{\mathrm{eff}}\left(T_{P}\right)<0$, then a transition to a polar smectic phase is observed. With further reduction of temperature, there are two possibilities. Even though $a_{\mathrm{eff}}$ approaches 0 with decreasing temperature, it might not reach it, so there is no formation of a nematic phase. On the other hand, if it becomes positive, one obtains a re‐entrant (polar) nematic phase below the polar smectic phase.

If $b_{P}$ in Equation (4) is positive, the phase transition to the polar phase is of the second order. In this case transition to the polar phase occurs at temperature $T^{*}$, and with reduction of temperature polarisation increases as

(8) $$P^{2}=\frac{1}{2c_{P}}\left(-b_{P}+\sqrt{b_{P}^{2}+4c_{P}a_{P0}\left(T^{*}-T\right)}\right).$$

At $T=T^{*}$, $a_{\mathrm{eff}}$ is still negative, but it increases towards 0 with decreasing temperature. At temperature $T_{\mathrm{rN}}$, where the condition

(9) $$T_{rN}-T_{\mathrm{NS}}+\frac{g_{2}}{a_{0}}P{\left(T_{rN}\right)}^{2}=0$$

is fulfilled, $a_{\mathrm{eff}}$ becomes 0 and then positive with further lowering of temperature. This means that at temperatures $T<T_{\mathrm{rN}}$ the smectic phase is not stable anymore and we have a transition to the (re‐entrant) nematic phase, which is polar. With further lowering of the temperature, the value $a_{\mathrm{eff}}$ will eventually start to decrease and become 0 at temperature $T_{\mathrm{rS}}$ given by

(10) $$T_{rS}-T_{\mathrm{NS}}+\frac{g_{2}}{a_{0}}P^{2}\left(T_{rS}\right)=0$$

So, at $T<T_{\mathrm{rS}}$ one observes a restoration of polar smectic phase.

We estimated the magnitudes of Landau parameters for the studied materials. For this purpose, we take *P* as a polar order parameter, with values between 0 and 1, defined as the ratio between the polarisation at some temperature and maximum polarisation. The phase behaviour of the *SR‐n‐Re* compounds can be explained by a weak first‐order phase transition to the polar phase and the behaviour of material *GS‐5‐Re* by a second‐order phase transition. The temperature dependence of $a_{\mathrm{eff}}/a_{0}$ for these materials is shown in Figure 5b. Not all the parameters can be calculated from the measured transition temperatures, so some values had to be estimated. For the *SR‐n‐Re* series, we assume that $P^{2}\left(T_{P}\right)=0.075$, which means $c_{P}=10b_{P}$. Because for homologues *n* = 6 and 7 a sequence N → SmA → N_F_ is observed, $a_{\mathrm{eff}}\left(T_{P}\right)>0$ and $T_{\mathrm{NS}}>T_{P}$. From this condition we find that $g_{2}>374a_{0}$ for *n* = 6 and $g_{2}>974a_{0}$ for *n* = 7. We chose $g_{2}=500a_{0}$ and $g_{2}=1000a_{0}$ for *n* = 6 and *n* = 7, respectively. At the temperature of the re‐entrant smectic phase $a_{\mathrm{eff}}\left(T_{\mathrm{rS}}\right)=0$, which gives $a_{P0}=1.15\cdot 10^{-3}b_{P}$ for *n* = 6 and $a_{P0}=1.7\cdot 10^{-4}b_{P}$ for *n* = 7. To model the phase sequence of *n* = 4, we estimate $T_{\mathrm{NS}}$ by a linear extrapolation of $T_{\mathrm{NS}}$ for *n* = 6 and 7. For this material, $T_{P}$ is the temperature at which a transition from N to N_F_ is observed. At $T=T_{P},a=a_{0}\left(T-T_{\mathrm{NS}}\right)$ *T* = *T* is still positive, so there is a direct transition from the nematic to the ferroelectric nematic phase. We assume that $g_{2}/a_{0}$ also reduces approximately linearly with reducing *n* and choose $g_{2}=70a_{0}$. By requiring $a_{\mathrm{eff}}\left(T_{\mathrm{rS}}\right)=0$, we obtain $a_{P0}=0.033b_{P}$. The transition temperatures taken to estimate the Landau parameters are given in Table S1.

For material GS‐5‐Re, we require $a_{\mathrm{eff}}\left(T_{\mathrm{rN}}\right)=0$ and $a_{\mathrm{eff}}\left(T_{\mathrm{rS}}\right)=0$. From these two conditions we find $a_{P0}c_{P}=0.413b_{P}^{2}$. If we assume that $P^{2}\left(T_{\mathrm{rN}}\right)=0.1$, we find also $c_{P}=0.014b_{P}$ and $a_{P0}=30b_{P}$ and $g_{2}=0.17a_{0}$.

In summary, in the field of LCs, re‐entrant phenomena—where the nematic phase reappears below the smectic phase as the temperature is lowered—are not unique and have been observed in many systems with competing interactions. However, in the materials studied here, this behaviour is rather unusual, as the melting of smectic layers is induced by polar order. The re‐entrant phenomenon can be explained within the framework of Landau theory, where the typical linear temperature dependence of the coefficient a is violated and becomes non‐monotonic near the transition to the polar phase. This non‐monotonicity is the reason for the re‐emergence of nematic order.

## Supporting Information

The authors have cited additional Refs. [28, 29, 30, 31], within the Supporting Information.

## Conflict of Interests

The authors declare no conflict of interest.

## Supporting information



Supporting Information

## Data Availability

The data that support the findings of this study are available in the supplementary material of this article.
