# Supplementary material for: Interplay of Polar Order and Positional Order in Liquid Crystals–Observation of Re‐entrant Ferroelectric Nematic Phase
Source: Angew Chem Int Ed Engl. 2025 Sep 30;64(46):e202516302. doi: 10.1002/anie.202516302 (PMC12603991; doi:10.1002/anie.202516302)
Supplement: Supplementary file 1 — Supporting Information [file ANIE-64-e202516302-s001.pdf]

Supporting Information

Interplay of Polar Order and Positional Order in Liquid Crystals – Observation of Re-entrant Ferroelectric Nematic Phase

Grant J. Strachan[a], Shona J. Ramsay[b], Marijus Juodka[b], Damian Pociecha[a], Jadwiga Szydlowska[a], John M.D. Storey[b], Natasa Vaupotic[c], Rebecca Walker[b], Ewa Gorecka [a]

[a] Grant J. Strachan, Jadwiga Szydlowska, Damian Pociecha*, Ewa Gorecka
Faculty of Chemistry
University of Warsaw
Zwirki i Wigury 101, Warsaw 02–089, Poland

E-mail: pociu@chem.uw.edu.pl

[b] Shona J. Ramsay, Marijus Juodka, John M.D. Storey, Rebecca Walker
School of Natural and Computing Sciences, University of Aberdeen, Aberdeen, Great Britain

[c] Natasa Vaupotic
Faculty of Natural Sciences and Mathematics, University of Maribor, Maribor, Slovenia; Jozef Stefan Institute, Ljubljana, Slovenia

1. Experimental methods 2
2. Additional results 3
3. Synthetic Procedures and Structural Characterisation 7
  3.1 Synthesis of SR-*n*-Re series 7
    General procedure for esterification SR-*n*-Re 8
    NMR Spectra SR-*n*-Re 13
  3.2 Synthesis of GS-*n*-Re series 22
    General Methods: 22
    NMR Spectra GS-*n*-Re 34
4. References 42

1. Experimental methods

Calorimetric studies: transition temperatures and the associated enthalpy changes were measured by differential scanning calorimetry using either TA DSC Q200 or Mettler Toledo DSC3 instrument. Measurements were performed under a nitrogen atmosphere with a heating/cooling rate of 5 or 10 K min^{-1}.

Optical Studies: Observations of optical textures of liquid crystalline phases was carried out by polarised-light optical microscopy using a Zeiss AxioImager.A2m microscope equipped with a Linkam heating stage. For the determination of optical axis direction and birefringence variation in the sample PolScope Abrio system mounted on the Zeiss microscope was used. Optical birefringence as a function of temperature was measured with a setup based on a photoelastic modulator (PEM-90, Hinds) working at a modulation frequency f = 50 kHz; as a light source a halogen lamp (Hamamatsu LC8) equipped with narrow bandpass filters was used. The transmitted light intensity was monitored with a photodiode (FLC Electronics PIN-20) and the signal was deconvoluted with a lock-in amplifier (EG&G 7265) into 1f and 2f components to yield a retardation induced by the sample. Knowing the sample thickness, the retardation was recalculated into optical birefringence. Samples were prepared in 1.6-μm-thick cells with planar anchoring. The alignment quality was checked prior to measurement by inspection under the polarised-light optical microscope.

X-ray diffraction studies: 2D-XRD patterns were registered using a Bruker D8 GADDS system, equipped with micro-focus-type X-ray source with Cu anode and dedicated optics and VANTEC2000 area detector. For precise measurements of layer structure small angle diffraction experiments were performed on a Bruker Nanostar system (IμS microfocus source with copper target, MRI heating stage, Vantec 2000 area detector).

Second Harmonic Generation: The SHG response was investigated using a home-made microscopic setup based on a solid-state laser EKSPLA NL202. Laser pulses (9 ns) at a 10 Hz repetition rate and max. 2 mJ pulse energy at λ=1064 nm were applied. The pulse energy was adjusted for each sample to avoid its decomposition. The infra-red beam was incident onto a cells with planar anchoring at both surfaces, the cell thickness was ~5 μm. An IR pass filter was placed at the entrance to the sample and a green pass filter at the exit. For controlling temperature Linkam stage was used. Optical SHG images were recorded with homemade microscopic setup.

Spontaneous Polarization Measurements: spontaneous electric polarisation was determined by integration of the current peaks recorded during polarization switching upon applying a triangular-wave voltage (60Hz, $200V_{pp}$). The repolarization current was measured by monitoring the voltage drop on the 100kOhm resistor in parallel connection with the LC cell. The cell with in-plane electric field was used, the ITO electrodes were deposited on one surface, the distance between electrodes was 2 millimetres. The thin polymer layers deposited on both surfaces ensured planar alignments of molecules. The cell thickness was 4 μm. The obtained value of the polarization should be treated with some caution as the electric field is not ideally uniform between electrodes, and the switching occurs also on the electrodes.

Dielectric spectroscopy: The complex dielectric permittivity was measured in the 1 Hz–10 MHz frequency range using a Solartron 1260 impedance analyzer. The material was placed in 10-μm-thick glass cell with gold electrodes (without surfactant to avoid the influence of the high capacitance of a thin polymer layer). The amplitude of the measuring ac voltage, 20 mV, was low enough to avoid Fréedericksz transition in ferroelectric nematic phases.

2. Additional results

Table S1: Phase transition temperatures (in °C) and entropy changes (scaled by gas constant, R) determined by DSC (on heating scans) for homologues of the SR-n-Re series.

n	*Phase sequence*
**1**	Cr 130 (18.01) N_F 207 (2.03) Iso
**2**	Cr 119 (15.18) [SmC_F 89[a]] N_F 194 (0.52) N 213 (0.60) Iso
**3**	Cr 96 (12.25) [SmC_F 91 (0.21)[b]] N_F 181[a] N_x 180 (0.33) N 217 (0.49) Iso
**4**	Cr 88 (17.91) SmC_F 102 (0.32) N_F 163 (0.21) N_x 168[a] N 210 (0.43) Iso
**5**	Cr 86 (11.95) SmC_F 97 (0.36) N_F 151[a] N_x 165 (0.15) N 211 (0.37) Iso
**6**	Cr 75 (15.25) SmC_F 95 (0.50) N_F 123 (0.39) SmA 151 (0.10) N 204 (0.36) Iso
**7**	Cr 95 (18.51) [SmC_F 85[a]] N_F 88[a] SmA 161 (0.13) N 206 (0.43) Iso

[a] Transition visible only on POM
[b] Data extracted from DSC cooling trace
[ ] represent monotropic transition

Table S2: Phase transition temperatures (in °C) and entropy changes (scaled by gas constant, R) determined by DSC (on heating scans) for homologues of the GS-n-Re series.

n	*Phase sequence*
**2**	Cr 105 (11.64) [SmC_F 66 (0.08)[a]] N_F 183 (0.10) N 234 (0.36) Iso
**3**	Cr 82 (8.11) SmC_F 90 (0.02) N_F 167 (0.06) N 225 (0.22) Iso
**4**	Cr 61 (5.85) SmC_F 90 (0.02) N_F 157 (0.07) N 229 (0.23) Iso
**5**	Cr 73 (2.20) SmC_F 91 (0.03) N_F 126[b] SmA_P 129[b] SmA 136 (0.01) N 209 (0.20) Iso
**6**	Cr 85 (15.24) [SmC_F 81 (0.40)] SmA 152 (0.04) N 204 (0.26) Iso
**7**	Cr 73 (4.33) [SmC_F 53 (0.54)] SmA 160 (0.04) N 196 (0.28) Iso

[a] Data extracted from DSC cooling trace
[b] Overlapping peaks on DSC trace, temperature taken from POM
[ ] represent monotropic transition

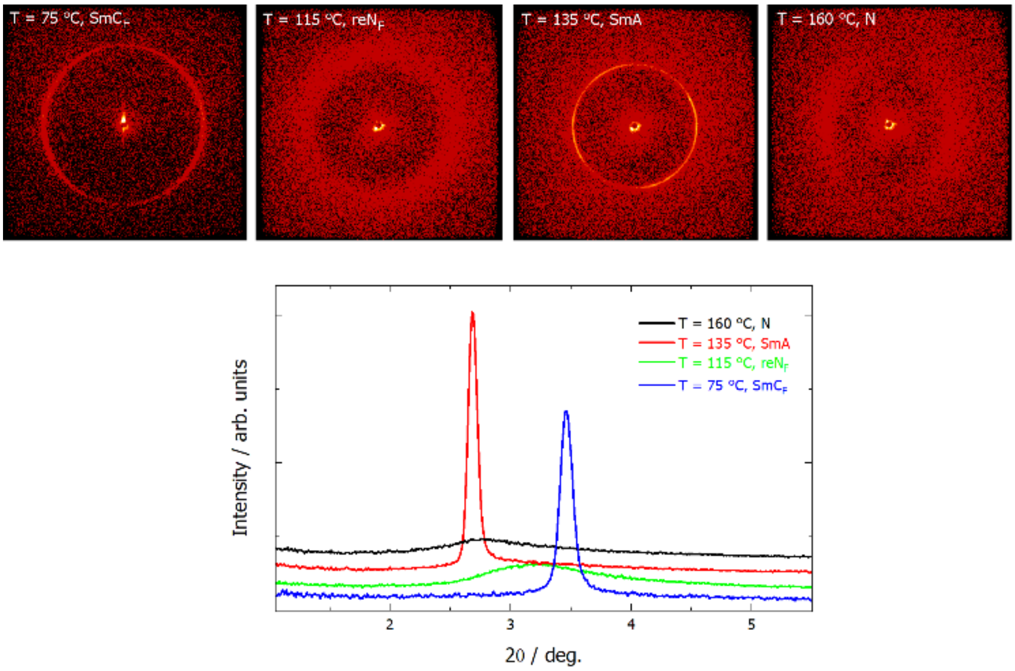

Figure S1: Small angle X-ray diffraction data for SR-6-Re: (upper raw) 2D diffraction patterns recorded in sequence of LC phases, (bottom) intensity of diffracted signal vs. diffraction angle obtained by integration of above patterns over azimuthal angle. Non-monotonic change of the positional order character between short-range in nematic phases and long-range in smectic phases is clearly evidenced by the width of small-angle diffraction signal.

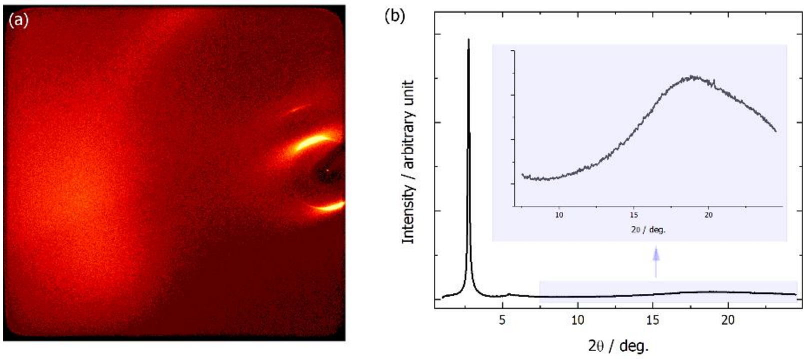

Figure S2: Liquid-like character of smectic phase formed by SR-6-Re: (a) 2D diffraction patterns recorded in broad angle range for SmA phase (T=135 °C), (b) intensity of diffracted signal vs. diffraction angle obtained by integration of 2D pattern over azimuthal angle. Diffused character of the high-angle signal (see inset) clearly evidences lack of the long-range positional order of molecules within smectic layers.

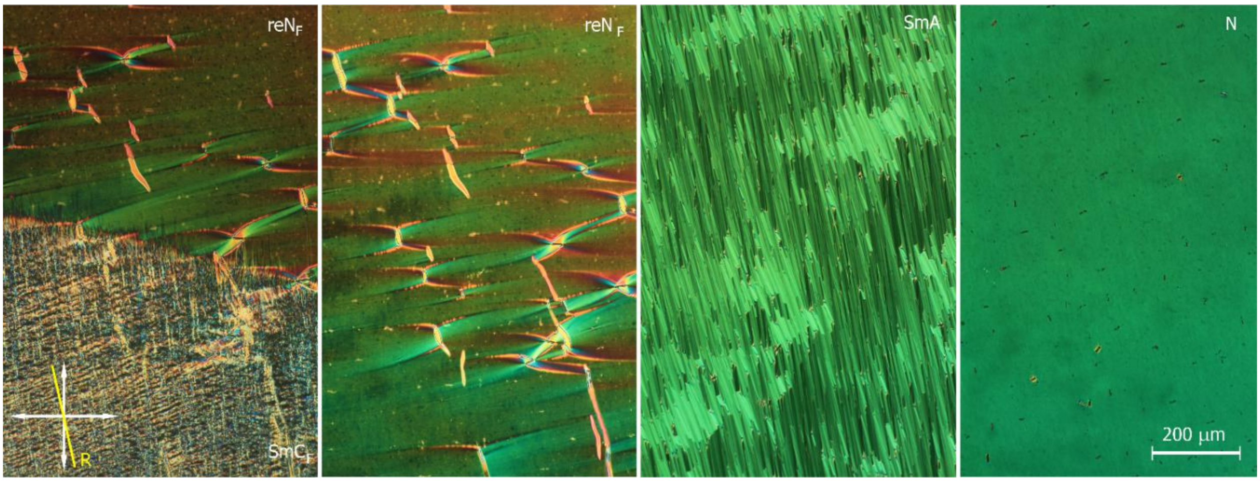

Figure S3: Textures of mesophases for SR-6-Re observed in 5-μm-thick cell with planar anchoring (rubbed at both surfaces unidirectionally). Polarizer and analyser were crossed (white arrows) while the rubbing direction (yellow line) was slightly inclined to polarizers. Note, that in left panel the transition from SmC$_F$ to reN$_F$ phase is observed.

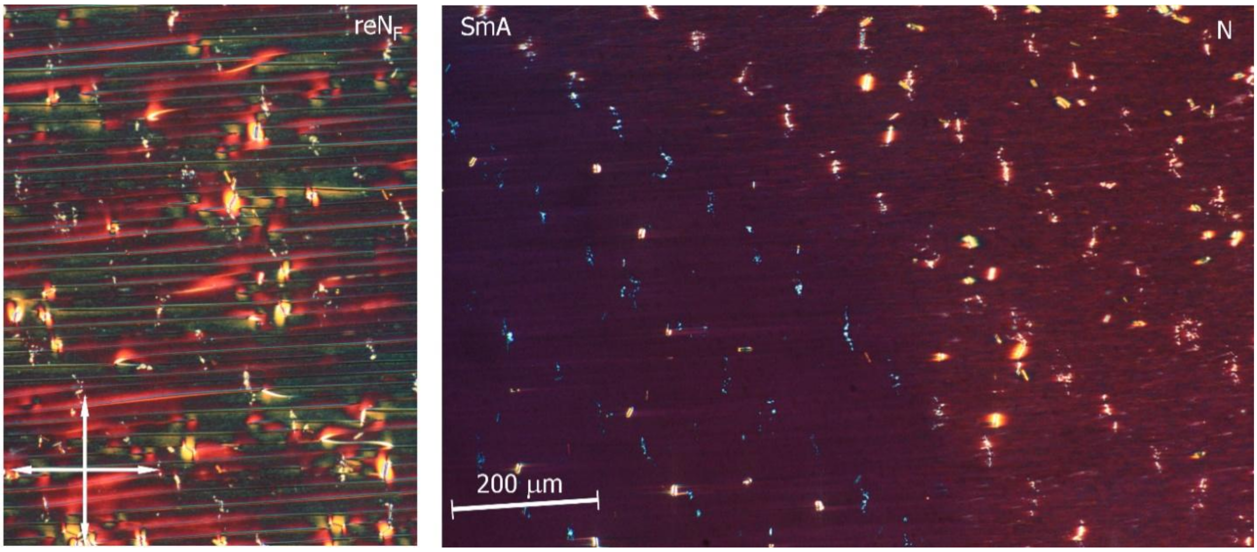

Figure S4: Textures of mesophases for GS-5-Re observed in 3-μm-thick cell with planar anchoring (rubbed at both surfaces unidirectionally). Right panel shows transition from N to SmA phase. Polarizer and analyser were crossed (white arrows), scale bar shows rubbing direction while the rubbing direction that was slightly inclined to polarizers.

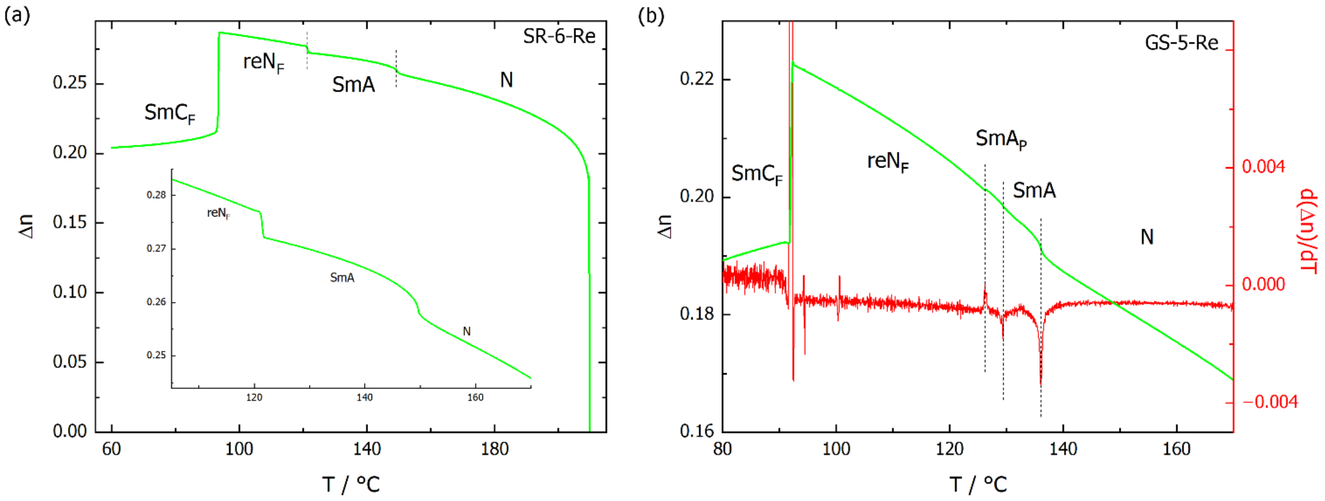

Figure S5: Temperature dependence of optical birefringence (green line) measured for green light (λ=532 nm) for compounds (a) SR-6-Re and (b) GS-5-Re. For GS-5-Re the derivative of Δn(T) is also shown (red line) to indicate the phase transition temperatures more clearly.

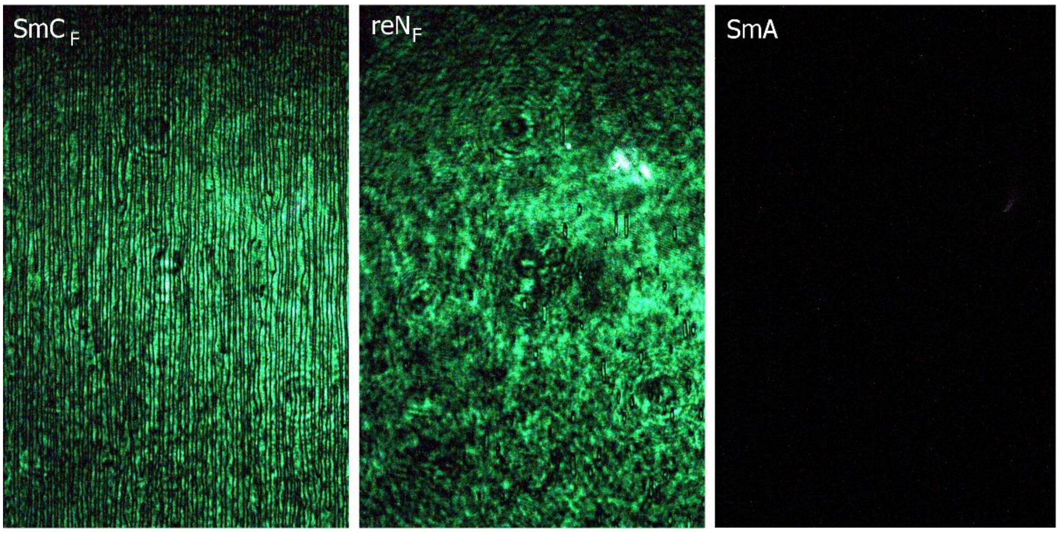

Figure S6: SHG microscopic images taken for GS-5-Re, SmA is SHG silent showing that the phase is non-polar.

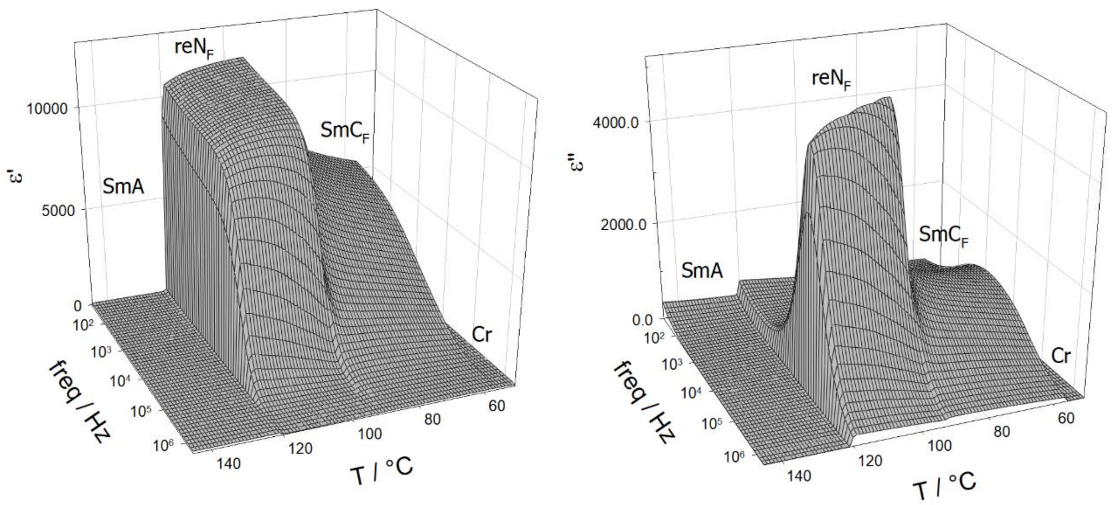

Figure S7: Apparent dielectric permittivity (real and imaginary part of Δε) vs. frequency and temperature for compound SR-6-Re. The strong dielectric response in reN$_F$ that appears below SmA shows the polar nature of the phase.

Table S3: Transition temperatures taken to model a_{eff} for materials shown in Figure 5; T_{NS} transition from the apolar nematic to the smectic phase, T_P to the polar phase, T_{rN} to the re-entrant nematic phase and T_{rS} to the re-entrant smectic phase

material	T_{NS} [°C]	T_P [°C]	T_{rN} [°C]	T_{rS} [°C]
SR-4-Re	130	163	/	102
SR-6-Re	151	123	123	95
SR-7-Re	161	88	88	85
GS-5-Re	136	130	127	93

3. Synthetic Procedures and Structural Characterisation

Unless otherwise stated, all materials were obtained from commercial sources and used without further purification. Reactions were monitored using thin layer chromatography (TLC) using aluminium-backed plates with a coating of Merck Kieselgel 60 F254 silica and an appropriate solvent system. Spots were visualised using UV light (254 nm). Flash column chromatography was carried out using silica grade 60 Å 40-63 micron. ^{1}H, ^{19}F, and ^{13}C NMR spectra were recorded on either a 400 MHz Agilent NMR spectrometer using either $CDCl_3$ or DMSO-d_6 as solvent and using residual non-deuterated trace solvents as reference. Chemical shifts (δ) are given in ppm relative to TMS (δ = 0.00 ppm). Coupling constants (J) are given in Hz and are $^{3}J_{HH}$ unless otherwise stated. Mass spectroscopy was conducted on a Micromass LCT instrument.

3.1 Synthesis of the SR-*n*-Re series

The synthetic route followed for series SR-*n*-Re is summarised in Scheme S1. Intermediates 1.1-4 were synthesised according to procedures detailed in Juodka *et al.*[28] ; intermediates 1.5 and 1.6 and final products followed procedures as in reference [29].

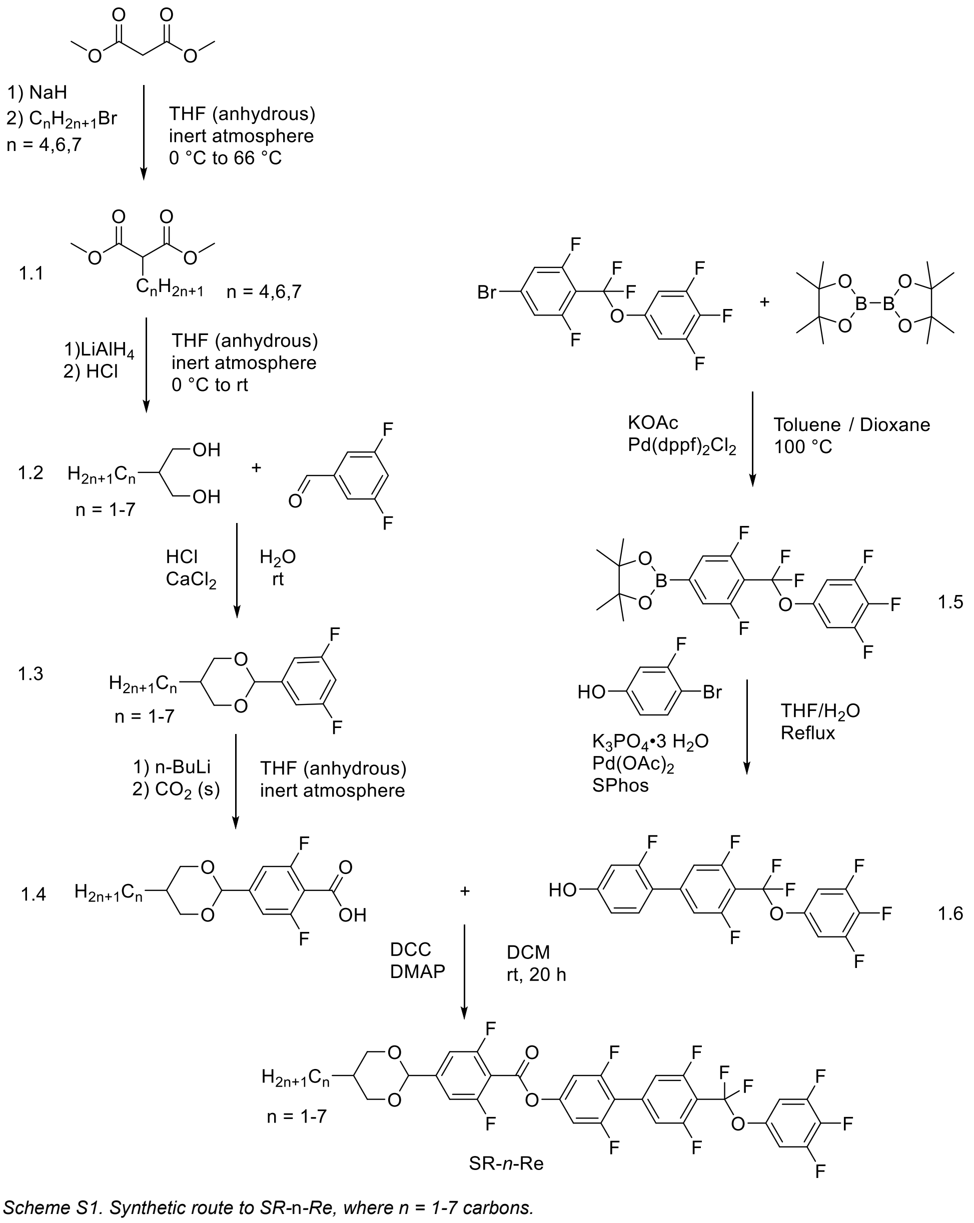

*Scheme S1. Synthetic route to SR-*n*-Re, where n = 1-7 carbons.*

General procedure for esterification SR-*n*-Re

The required 4-(5-*alkyl*-1,3-dioxan-2-yl)-2,6-difluorobenzoic acid (1.4) (1 eq) and DCC (1.3 eq) were dissolved in DCM (10 mL) and stirred until the mixture turned opaque. 1.6 (1.1 eq) and DMAP (0.13 eq) were added and the reaction mixture stirred for 18 hours. The solvent was removed *in vacuo* and the crude product purified by flash column chromatography (DCM, followed by 1:9 ethyl acetate:petroleum ether (40-60)) and subsequent recrystallisation in ethanol to yield the title compound as a white solid.

*Table S4. Quantities of reagents used in the synthesis of SR-*n*-Re where n = 1-7.*

	4-(5-*alkyl*-1,3-dioxan-2-yl)-2,6-difluorobenzoic acid 1.4		4'-(difluoro(3,4,5-trifluorophenoxy)methyl)-2,3',5',6-tetrafluoro-[1,1'-biphenyl]-4-ol 1.6	
n	**Mass (g)**	**Moles (mmol)**	**Mass (g)**	**Moles (mmol)**
1	0.059	0.228	0.101	0.230
2	0.056	0.206	0.100	0.228
3	0.059	0.206	0.100	0.228
4	0.065	0.216	0.100	0.228
5	0.069	0.220	0.101	0.230
6	0.068	0.207	0.102	0.233
7	0.075	0.219	0.099	0.226

4'-(difluoro(3,4,5-trifluorophenoxy)methyl)-2,3',5',6-tetrafluoro-[1,1'-biphenyl]-4-yl 2,6-difluoro-4-(5-methyl-1,3-dioxan-2-yl)benzoate (SR-1-Re)

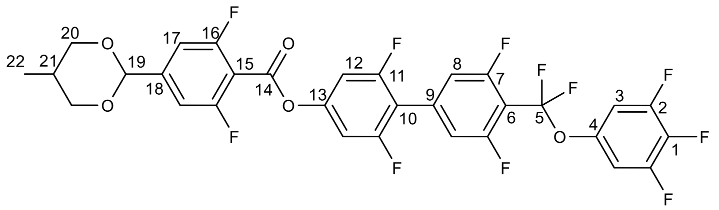

Yield: 0.045g (29%) R_F: 0.28 (1:9 ethyl acetate: petroleum ether (40-60))

m.p. 130 °C, $T_{NF\text{-}I}$ 208 °C

^{1}H NMR (400 MHz, $CDCl_3$) δ ppm: 7.21 (d, $^3J_{HF}$ = 9.4 Hz, 2H, Ar-**H**, H_{17}), 7.16 (d, $^3J_{HF}$ = 10.5 Hz, 2H, Ar-**H**, H_{12}), 7.08 – 7.03 ($m_{overlapping\ d}$, 2H, Ar-**H**, H_8), 7.02 – 6.97 ($m_{overlapping\ d}$, 2H, H_3), 5.41 (s, 1H, R-CH-$(CH_2)_2$-$(O)_2$-**CH**-Ar, H_{19}), 4.28 – 4.17 (m, 2H, R-CH-(**CH_2**)$_2$-$(O)_2$-CH-Ar, H_{20}), 3.59 – 3.45 (m, 2H, R-CH-(**CH_2**)$_2$-$(O)_2$-CH-Ar, H_{20}), 2.30 – 2.14 (m, 1H, R-**CH**-$(CH_2)_2$-$(O)_2$-CH-Ar, H_{21}), 0.80 (d, $^3J_{H\text{-}H}$ = 6.7 Hz, 3H, **CH_3**-Dioxane, H_{22})

^{19}F NMR (proton decoupled) (376 MHz, $CDCl_3$) δ ppm: -61.98 (t, $^4J_{F\text{-}F}$ = 26.8 Hz, 2F, **F_5**), -108.14 (s, 2F, **F_{16}**), -110.52 (t, $^4J_{F\text{-}F}$ = 27.8 Hz, 2F, **F_7**), -111.70 (s, 2F, **F_{11}**), -132.42 (d, $^3J_{F\text{-}F}$ = 20.8 Hz, 2F, **F_2**), -163.06 (t, $^3J_{F\text{-}F}$ = 20.3 Hz, 1F, **F_1**)

^{13}C NMR (101 MHz, $CDCl_3$) δ ppm: 161.14 (dd, $^1J_{CF}$ = 259.0, $^3J_{CF}$ = 5.5 Hz, 2C), 161.32 – 158.47 ($m_{overlapping\ signals,}$ 4C), 158.93 (s, apparent t, 1C), 151.44 (t, $^3J_{CF}$ = 14.2 Hz, 1C), 151.17 (ddd, $^1J_{CF}$ = 251.2, $^2J_{CF}$ = 10.7 Hz, $^3J_{CF}$ = 5.3 Hz, 2C), 146.13 (t, $^3J_{CF}$ = 10.0 Hz, 1C), 144.84 – 144.50 (m, 1C), 139.27 (dt, $^1J_{CF}$ = 250.4, $^2J_{CF}$ = 14.9 Hz, 1C), 134.42 (t, $^3J_{CF}$ = 11.4 Hz, 1C), 120.16 (t, $^1J_{CF}$ = 266.9 Hz, 1C), 114.90 (dd, $^2J_{CF}$ = 24.4, $^4J_{CF}$ = 2.3 Hz, 2C), 113.49 (t, $^2J_{CF}$ = 17.8 Hz, 1C, app dt), 110.55 (dd, $^2J_{CF}$ = 23.6 Hz, $^4J_{CF}$ = 3.4 Hz, 2C), 109.87 (t, $^2J_{CF}$ = 13.8 Hz, 1C), 109.04 (t, $^2J_{CF}$ = 16.7 Hz, 1C), 107.66 (dd, $^2J_{CF}$ = 23.8, $^4J_{CF}$ = 6.5 Hz, 2C), 106.86 (ddd, $^2J_{CF}$ = 21.7 Hz, $^3J_{CF}$ 8.4 Hz, $^4J_{CF}$ = 2.6 Hz, 2C), 98.64 (t, $^4J_{CF}$ = 2.3 Hz, 1C), 73.75 (2C), 29.40 (1C), 12.37 (1C)

IR ν_{max} (cm^{-1}): 3107 (C-H stretch, sp^2 hybridised), 2959 (C-H stretch, sp^3 hybridised), 2111 (C-H bend, overtones), 1744 (C=O stretch, ester)

4'-(difluoro(3,4,5-trifluorophenoxy)methyl)-2,3',5',6-tetrafluoro-[1,1'-biphenyl]-4-yl 4-(5-ethyl-1,3-dioxan-2-yl)-2,6-difluorobenzoate (SR-2-Re)

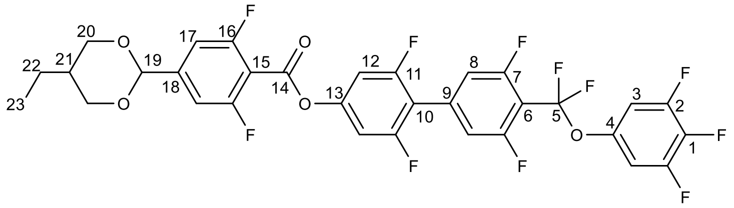

Yield: 0.033g (21%), R_F 0.40 (1:9 ethyl acetate:petroleum ether (40-60))

m.p. 119 °C, ($T_{SmCF-NF}$ 89 °C), T_{NF-N} 192 °C, T_{N-I} 215 °C

^{1}H NMR (400 MHz, $CDCl_3$) δ ppm: 7.20 (d, $^3J_{HF}$ = 9.3 Hz, 2H, Ar-**H**, H_{17}), 7.16 (d, $^3J_{HF}$ = 10.5 Hz, 2H, Ar-**H**, H_{12}), 7.08 – 7.03 ($m_{overlapping\ d}$, 2H, Ar-**H**, H_8), 7.02 – 6.96 ($m_{overlapping\ d}$, 2H, Ar-**H**, H_3), 5.40 (s, 1H, R-CH-$(CH_2)_2$-$(O)_2$-**CH**-Ar, H_{19}), 4.31 – 4.24 (m, 2H, R-CH-(**CH_2**)$_2$-$(O)_2$-CH-Ar, H_{20}), 3.58 – 3.49 (m, 2H, R-CH-(**CH_2**)$_2$-$(O)_2$-CH-Ar, H_{20}), 2.12 – 1.98 (m, 1H, R-**CH**-$(CH_2)_2$-$(O)_2$-CH-Ar, H_{21}), 1.18 (p, $^3J_{H-H}$ = 7.5 Hz, 2H, CH_3-**CH_2**-Dioxane, H_{22}), 0.95 (t, J_{H-H} = 7.5 Hz, 3H, **CH_3**-CH_2-Dioxane, H_{23})

^{19}F NMR (proton decoupled) (376 MHz, $CDCl_3$) δ ppm: -61.93 (t, $^4J_{F-F}$ = 27.5 Hz, 2F, **F_5**), -108.15 (s, 2F, **F_{16}**), -110.50 (t, $^4J_{F-F}$ = 27.8 Hz, 2F, **F_7**), -111.70 (s, 2F, **F_{11}**), -132.42 (d, $^3J_{F-F}$ = 20.7 Hz, 2F, **F_2**), -163.06 (t, $^3J_{F-F}$ = 20.4 Hz, 1F, **F_1**)

^{13}C NMR (101 MHz, $CDCl_3$) δ ppm: 161.14 (dd, $^1J_{CF}$ = 259.0 Hz, $^1J_{CF}$ = 5.6 Hz, 2C), 161.29 – 158.46 ($m_{overlapping\ signals}$, 4C), 158.94 (s, apparent t, 1C), 151.44 (t, $^3J_{CF}$ = 14.3 Hz, 1C), 151.17 (ddd, $^1J_{CF}$ = 250.9 Hz, $^2J_{CF}$ =10.6 Hz, $^3J_{CF}$ = 5.2 Hz, 2C), 146.18 (t, $^3J_{CF}$ = 9.9 Hz, 1C), 144.88 – 144.47 (m, 1C), 138.65 (dt, $^1J_{CF}$ = 250.4, $^2J_{CF}$ = 15.1 Hz, 1C), 134.41 (t, $^3J_{CF}$ = 11.5 Hz, 1C), 120.17 (t, $^1J_{CF}$ = 267.1 Hz, 1C), 114.86 (dd, $^2J_{CF}$ = 24.1, $^4J_{CF}$ = 2.2 Hz, 2C), 113.59 (t, $^2J_{CF}$ = 17.7 Hz, 1C), 110.55 (dd, $^2J_{CF}$ = 23.7, $^4J_{CF}$ = 3.4 Hz, 2C), 109.90 (t, $^2J_{CF}$ = 14.2 Hz, 1C), 109.05 (t, $^2J_{CF}$ = 16.8 Hz, 1C), 107.67 (dd, $^2J_{CF}$ = 23.9, $^4J_{CF}$ = 6.1 Hz, 2C), 106.87 (ddd, $^2J_{CF}$ = 22.0, $^3J_{CF}$ = 8.3, $^4J_{CF}$ = 2.6 Hz, 2C), 98.88 (t, $^4J_{CF}$ = 2.2 Hz, 1C), 72.55 (2C), 35.86 (1C), 21.25 (1C), 11.03 (1C)

IR ν_{max} (cm^{-1}): 3072 (C-H stretch, sp^2 hybridised), 2917 (C-H stretch, sp^3 hybridised), 2106 (C-H bend, overtones), 1762 (C=O stretch, ester)

4'-(difluoro(3,4,5-trifluorophenoxy)methyl)-2,3',5',6-tetrafluoro-[1,1'-biphenyl]-4-yl 4-(5-propyl-1,3-dioxan-2-yl)-2,6-difluorobenzoate (SR-3-Re)

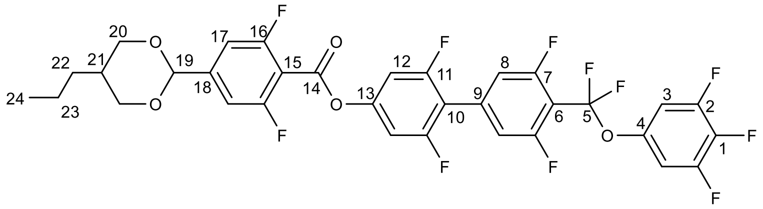

Yield: 0.040g (27%), R_F: 0.47 (1:9 ethyl acetate:petroleum ether (40-60))

m.p. 96 °C, ($T_{SmCF-NF}$ 91 °C), T_{NF-Nx} 180 °C, T_{Nx-N} 181 °C, T_{N-I} 215 °C

^{1}H NMR (400 MHz, $CDCl_3$) δ ppm: 7.20 (d, $^3J_{HF}$ = 9.2 Hz, 2H, Ar-**H**, H_{17}), 7.16 (d, $^3J_{HF}$ = 10.4 Hz, 2H, Ar-**H**, H_{12}), 7.08 – 7.03 ($m_{overlapping\ d}$, 2H, Ar-**H**, H_8), 7.02 – 6.97 ($m_{overlapping\ d}$, 2H, Ar-**H**, H_3), 5.40 (s, 1H, R-CH-$(CH_2)_2$-$(O)_2$-**CH**-Ar, H_{19}), 4.29 – 4.23 (m, 2H, R-CH-(**CH_2**)$_2$-$(O)_2$-CH-Ar, H_{20}), 3.58 – 3.50 (m, 2H, R-CH-(**CH_2**)$_2$-$(O)_2$-CH-Ar, H_{20}), 2.19 – 2.09 (m, 1H, R-**CH**-$(CH_2)_2$-$(O)_2$-CH-Ar, H_{21}), 1.35 (h, $^3J_{H-H}$ = 7.3 Hz, 2H, CH_3-**CH_2**-CH_2-Dioxane, H_{23}), 1.11 (q, $^3J_{H-H}$ = 7.3 Hz, 2H, CH_3-CH_2-**CH_2**-Dioxane, H_{22}), 0.94 (t, $^3J_{H-H}$ = 7.3 Hz, 3H, **CH_3**-CH_2-CH_2-Dioxane, H_{24})

^{19}F NMR (proton decoupled) (376 MHz, $CDCl_3$) δ ppm: -61.95 (t, $^4J_{F-F}$ = 26.5 Hz, 2F, **F_5**), -108.14 (s, 2F, **F_{16}**), -110.52 (t, $^4J_{F-F}$ = 27.0 Hz, 2F, **F_7**), -111.70 (s, 2F, **F_{11}**), -132.42 (d, $^3J_{F-F}$ = 20.7 Hz, 2F, **F_2**), -163.05 (t, $^3J_{F-F}$ = 20.5 Hz, 1F, **F_1**)

^{13}C NMR (101 MHz, $CDCl_3$) δ ppm: 161.01 (dd, $^1J_{CF}$ = 259.0 Hz, $^1J_{CF}$ = 5.5 Hz, 2C), 161.17 – 158.32 ($m_{overlapping\ signals}$, 4C), 158.80 (s, apparent t, 1C), 151.47 (ddd, $^1J_{CF}$ = 250.9 Hz, $^2J_{CF}$ = 10.6 Hz, $^3J_{CF}$ 5.3 Hz, 2C), 151.30 (t, $^3J_{CF}$ = 14.3 Hz, 1C), 146.05 (t, $^3J_{CF}$ = 10.0 Hz, 1C), 144.73 – 144.34 (m, 1C), 138.52 (dt, $^1J_{CF}$ = 250.4 Hz, $^2J_{CF}$ = 15.8 Hz, 1C), 134.28 (t, $^3J_{CF}$ = 11.4 Hz, 1C), 120.03 (t, $^1J_{CF}$ = 266.2 Hz, 1C), 114.77 (dd, $^2J_{CF}$ = 24.4 Hz, $^4J_{CF}$ = 2.0 Hz, 2C), 113.62 – 113.11 (m, 1C), 110.40 (dd, $^2J_{CF}$ = 23.5 Hz, $^4J_{CF}$ = 3.4 Hz, 2C), 109.74 (t, $^2J_{CF}$ = 13.5 Hz, 1C), 108.91 (t, $^2J_{CF}$ = 16.4 Hz, 1C), 107.53 (dd, $^2J_{CF}$ = 23.9, $^4J_{CF}$ = 6.4 Hz, 2C), 106.72 (ddd, $^2J_{CF}$ = 21.9 Hz, $^3J_{CF}$ = 8.5 Hz, $^4J_{CF}$ = 2.6 Hz, 2C), 98.73 (t, $^4J_{CF}$ = 2.3 Hz, 1C), 72.60 (2C), 33.90 (1C), 30.22 (1C), 19.53 (1C), 14.19 (1C)

IR ν_{max} (cm^{-1}): 3109 (C-H stretch, sp^2 hybridised), 2926 (C-H stretch, sp^3 hybridised), 2121 (C-H bend, overtones), 1750 (C=O stretch, ester)

4'-(difluoro(3,4,5-trifluorophenoxy)methyl)-2,3',5',6-tetrafluoro-[1,1'-biphenyl]-4-yl 4-(5-butyl-1,3-dioxan-2-yl)-2,6-difluorobenzoate (SR-4-Re)

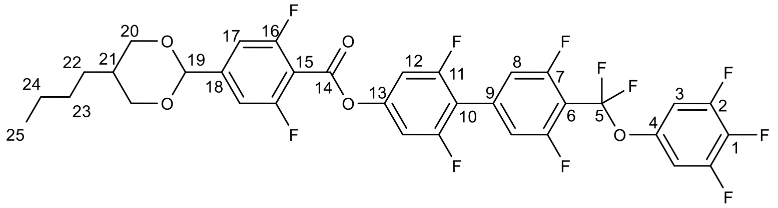

Yield: 0.055 (35%), R_F: 0.50 (1:9 ethyl acetate:petroleum ether (40-60))

m.p. 89 °C, $T_{SmCF-NF}$ 102 °C, T_{NF-N} 163 °C, T_{N-I} 210 °C

^{1}H NMR (400 MHz, $CDCl_3$) δ ppm: 7.23 (d, $^3J_{HF}$ = 9.5 Hz, 2H, Ar-**H**, H_{17}), 7.19 (d, $^3J_{HF}$ = 10.5 Hz, 2H, Ar-**H**, H_{12}), 7.11 – 7.05 ($m_{overlapping\ d}$, 2H, Ar-**H**, H_8), 7.05 – 7.00 ($m_{overlapping\ d}$, 2H, Ar-**H**, H_3), 5.43 (s, 1H, R-CH-$(CH_2)_2$-$(O)_2$-**CH**-Ar, H_{19}), 4.33 – 4.23 (m, 2H, R-CH-(**CH_2**)$_2$-$(O)_2$-CH-Ar, H_{20}), 3.63 – 3.51 (m, 2H, R-CH-(**CH_2**)$_2$-$(O)_2$-CH-Ar, H_{20}), 2.23 – 2.08 (m, 1H, R-**CH**-$(CH_2)_2$-$(O)_2$-CH-Ar, H_{21}), 1.40 – 1.28 (m, 4H, CH_3-**CH_2-CH_2**-CH_2-Dioxane, H_{23-24}), 1.15 (q, $^3J_{H-H}$ = 7.0 Hz, 2H, CH_3-CH_2-CH_2-**CH_2**-Dioxane, H_{22}), 0.94 (t, $^3J_{H-H}$ = 6.9 Hz, 3H, **CH_3**-CH_2-CH_2-CH_2-Dioxane, H_{25})

^{19}F NMR (proton decoupled) (376 MHz, $CDCl_3$) δ ppm: -61.95 (t, $^4J_{F-F}$ = 26.6 Hz, 2F, **F_5**), -108.14 (s, 2F, **F_{16}**), -110.52 (t, $^4J_{F-F}$ = 26.7 Hz, 2F, **F_7**), -111.70 (s, 2F, **F_{11}**), -132.42 (d, $^3J_{F-F}$ = 20.9 Hz, 2F, **F_2**), -163.03 (t, $^3J_{F-F}$ = 20.6 Hz, 1F, **F_1**)

^{13}C NMR (101 MHz, $CDCl_3$) δ ppm: 161.14 (dd, $^1J_{CF}$ = 259.0, $^3J_{CF}$ = 5.5 Hz, 2C), 161.28 – 158.46 ($m_{overlapping}$, 4C), 158.94 (s, apparent t, 1C), 151.44 (t, $^3J_{CF}$ = 14.4 Hz, 1C), 151.17 (ddd, $^1J_{CF}$ = 250.7 Hz, $^2J_{CF}$ = 10.7 Hz, $^3J_{CF}$ = 5.1 Hz, 2C), 146.18 (t, $^3J_{CF}$ = 10.0 Hz, 1C), 144.87 – 144.46 (m, 1C), 139.90 (dt, $^1J_{CF}$ = 250.5, $^2J_{CF}$ = 15.2 Hz, 1C), 134.41 (t, $^3J_{CF}$ = 11.4 Hz, 1C), 120.17 (t, $^1J_{CF}$ = 266.7 Hz, 1C), 114.90 (dd, $^2J_{CF}$ = 24.2, $^4J_{CF}$ = 2.3 Hz, 2C), 113.50 (t, $^2J_{CF}$ = 17.4 Hz, 1C), 110.54 (dd, $^2J_{CF}$ = 23.6, $^4J_{CF}$ = 3.4 Hz, 2C), 109.88 (t, $^2J_{CF}$ = 14.0 Hz, 1C), 109.05 (t, $^2J_{CF}$ = 16.6 Hz, 1C), 107.66 (dd, $^2J_{CF}$ = 23.7, $^4J_{CF}$ = 6.5 Hz, 2C), 106.84 (ddd, $^2J_{CF}$ = 21.8, $^3J_{CF}$ = 8.2, $^4J_{CF}$ = 2.5 Hz, 2C), 98.87 ($^4J_{CF}$, J = 2.2 Hz, 1C), 72.77 (2C), 34.27 (1C), 28.60 (1C), 27.92 (1C), 22.95 (1C), 14.02 (1C)

IR ν_{max} (cm^{-1}): 3107 (C-H stretch, sp^2 hybridised), 2925 (C-H stretch, sp^3 hybridised), 2109 (C-H bend, overtones), 1747 (C=O stretch, ester)

4'-(difluoro(3,4,5-trifluorophenoxy)methyl)-2,3',5',6-tetrafluoro-[1,1'-biphenyl]-4-yl 4-(1,3-dioxan-2-pentyl)-2,6-difluorobenzoate (SR-5-Re)

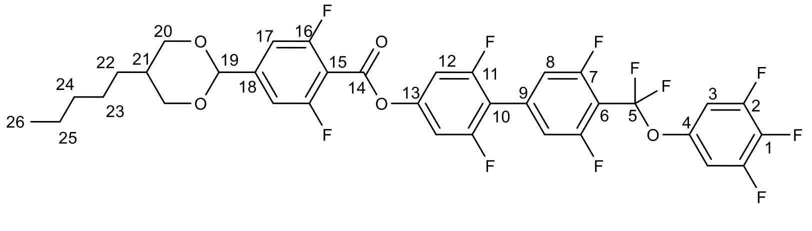

Yield: 0.038g (24%), R_F: 0.52 (1:9 ethyl acetate:petroleum ether (40-60))

m.p. 86 °C, $T_{SmCF-NF}$ 97 °C, T_{NF-Nx} 151 °C, T_{Nx-N} 165 °C, T_{N-I} 210 °C

^{1}H NMR (400 MHz, $CDCl_3$) δ ppm: 7.20 (d, $^3J_{HF}$ = 9.3 Hz, 2H, Ar-**H**, H_{17}), 7.16 (d, $^3J_{HF}$ = 10.5 Hz, 2H, Ar-**H**, H_{12}), 7.08 – 7.03 ($m_{overlapping\ d}$, 2H, Ar-**H**, H_8), 7.03 – 6.97 ($m_{overlapping\ d}$, 2H, Ar-**H**, H_3), 5.40 (s, 1H, R-CH-$(CH_2)_2$-$(O)_2$-**CH**-Ar, H_{19}), 4.30 – 4.22 (m, 2H, R-CH-(**CH_2**)$_2$-$(O)_2$-CH-Ar, H_{20}), 3.59 – 3.48 (m, 2H, R-CH-(**CH_2**)$_2$-$(O)_2$-CH-Ar, H_{20}), 2.19 – 2.06 (m, 1H, R-**CH**-$(CH_2)_2$-$(O)_2$-CH-Ar, H_{21}), 1.40 – 1.23 (m, 6H, CH_3-CH_2-**CH_2-CH_2-CH_2**-Dioxane, H_{22-24}), 1.17 – 1.07 (m, 2H, CH_3-**CH_2**-CH_2-CH_2-CH_2-Dioxane, H_{25}), 0.94 – 0.86 (m, 3H, **CH_3**-CH_2-CH_2-CH_2-CH_2-Dioxane, H_{26})

^{19}F NMR (proton decoupled) (376 MHz, $CDCl_3$) δ ppm: -61.95 (t, $^4J_{F-F}$ = 26.6 Hz, 2F, **F_5**), -108.14 (s, 2F, **F_{16}**), -110.52 (t, $^4J_{F-F}$ = 26.7 Hz, 2F, **F_7**), -111.70 (s, 2F, **F_{11}**), -132.42 (d, $^3J_{F-F}$ = 20.9 Hz, 2F, **F_2**), -163.06 (t, $^3J_{F-F}$ = 20.7 Hz, 1F, **F_1**)

^{13}C NMR (101 MHz, $CDCl_3$) δ ppm: 161.14 (dd, $^1J_{CF}$ = 258.9 Hz, $^3J_{CF}$ = 5.5 Hz, 2C), 161.27 – 158.46 ($m_{overlapping\ signals}$, 4C), 158.93 (s, apparent t, 1C), 151.44 (t, $^3J_{CF}$ = 14.3 Hz, 1C), 151.17 (ddd, $^1J_{CF}$ = 251.0 Hz, $^2J_{CF}$ = 10.7 Hz, $^3J_{CF}$ = 5.2 Hz, 2C), 146.19 (t, $^3J_{CF}$ = 10.1 Hz, 1C), 144.87 – 144.48 (m, 1C), 138.65 (dt, $^1J_{CF}$ = 250.5 Hz, $^2J_{CF}$ = 15.0 Hz, 1C), 134.41 (t, $^3J_{CF}$ = 11.4 Hz, 1C), 120.16 (t, $^1J_{CF}$ = 267.0 Hz, 1C), 114.90 (dd, $^2J_{CF}$ = 24.4 Hz, $^4J_{CF}$ = 2.4 Hz, 2C), 113.76 – 113.25 (m, 1C), 110.54 (dd, $^2J_{CF}$ = 23.7 Hz, $^4J_{CF}$ = 3.4 Hz, 2C), 109.81 (t, $^2J_{CF}$ = 14.1 Hz, 1C), 109.04 ($^2J_{CF}$, J = 16.6 Hz, 1C), 107.66 (dd, $^2J_{CF}$ = 24.0 Hz, $^4J_{CF}$ = 6.5 Hz, 2C), 106.86 (ddd, $^2J_{CF}$ = 21.9 Hz, $^3J_{CF}$ = 8.4 Hz, $^4J_{CF}$ = 2.7 Hz, 2C), 98.87 (t, $^4J_{CF}$ = 2.2 Hz, 4C), 72.77 (2C), 34.27 (1C), 32.05 (1C), 28.17 (1C), 26.10 (1C), 22.60 (1C), 14.15 (1C)

IR v_{max} (cm^{-1}): 3077 (C-H stretch, sp^2 hybridised), 2925 (C-H stretch, sp^3 hybridised), 2033 (C-H bend, overtones), 1750 (C=O stretch, ester)

4'-(difluoro(3,4,5-trifluorophenoxy)methyl)-2,3',5',6-tetrafluoro-[1,1'-biphenyl]-4-yl 4-(1,3-dioxan-2-hexyl)-2,6-difluorobenzoate (SR-6-Re)

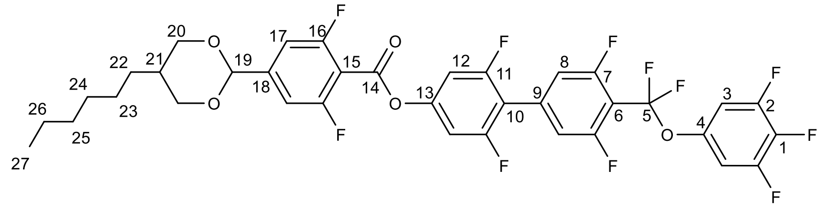

Yield: 0.036 (23%), R_F: 0.53 (1:9 ethyl acetate:petroleum ether (40-60))

m.p.75 °C, $T_{SmCF\text{-}NF}$ 95 °C, $T_{NF\text{-}SmA}$ 123 °C, $T_{SmA\text{-}N}$ 151 °C, $T_{N\text{-}I}$ 204 °C

^{1}H NMR (400 MHz, $CDCl_3$) δ ppm: 7.20 (d, $^3J_{HF}$ = 9.3 Hz, 2H, Ar-**H**, H_{17}), 7.16 (d, $^3J_{HF}$ = 10.5 Hz, 2H, Ar-**H**, H_{12}), 7.08 – 7.03 ($m_{overlapping\ d}$, 2H, Ar-**H**, H_8), 7.03 – 6.97 ($m_{overlapping\ d}$, 2H, Ar-**H**, H_3), 5.40 (s, 1H, R-CH-$(CH_2)_2$-$(O)_2$-**CH**-Ar, H_{19}), 4.29 – 4.21 (m, 2H, R-CH-(**CH_2**)$_2$-$(O)_2$-CH-Ar, H_{20}), 3.59 – 3.50 (m, 2H, R-CH-(**CH_2**)$_2$-$(O)_2$-CH-Ar, H_{20}), 2.19 – 2.05 (m, 1H, R-**CH**-$(CH_2)_2$-$(O)_2$-CH-Ar, H_{21}), 1.37 – 1.23 (m, 8H, CH_3-CH_2-**CH_2-CH_2-CH_2-CH_2**-Dioxane, $H_{22\text{-}25}$), 1.16 – 1.07 (m, 2H, CH_3-**CH_2**-CH_2-CH_2-CH_2-CH_2-Dioxane, H_{26}), 0.95 – 0.85 (m, 3H, **CH_3**-CH_2-CH_2-CH_2-CH_2-CH_2-Dioxane, H_{27})

^{19}F NMR (proton decoupled) (376 MHz, $CDCl_3$) δ ppm: -61.95 (t, $^4J_{F\text{-}F}$ = 27.0 Hz, 2F, **F_5**), -108.14 (s, 2F, **F_{16}**), -110.52 (t, $^4J_{F\text{-}F}$ = 27.0 Hz, 2F, **F_7**), -111.70 (s, 2F, **F_{11}**), -132.42 (d, $^3J_{F\text{-}F}$ = 21.2 Hz, 2F, **F_2**), -163.05 (t, $^3J_{F\text{-}F}$ = 20.9 Hz, 1F, **F_1**)

^{13}C NMR (101 MHz, $CDCl_3$) δ ppm: 161.14 (dd, $^1J_{CF}$ = 258.9 Hz, $^3J_{CF}$ = 5.6 Hz, 2C), 161.27 – 158.46 ($m_{overlapping\ signals}$, 4C), 158.94 (s, apparent t, 1C), 151.58 (ddd, $^1J_{CF}$ = 250.9 Hz, $^2J_{CF}$ = 10.4 Hz, $^3J_{CF}$ = 4.9 Hz, 2C), 151.44 (t, $^3J_{CF}$ = 14.3 Hz, 1C), 146.19 (t, $^3J_{CF}$ = 9.9 Hz, 1C), 144.91 – 144.42 (m, 1C), 138.65 (dt, $^1J_{CF}$ = 250.6 Hz, $^2J_{CF}$ = 15.2 Hz, 1C), 134.41 (t, $^3J_{CF}$ = 11.5 Hz, 1C), 118.84 (t, $^1J_{CF}$ = 266.0 Hz, 1C), 114.91 (dd, $^2J_{CF}$ = 24.3 Hz, $^4J_{CF}$ = 2.4 Hz, 2C), 113.50 (t, $^2J_{CF}$ = 17.1 Hz, 1C), 110.54 (dd, $^2J_{CF}$ = 23.6, $^4J_{CF}$ = 3.4 Hz, 2C), 109.88 (t, $^2J_{CF}$ = 14.1 Hz, 1C), 108.96 (t, $^2J_{CF}$ = 16.8 Hz, 1C), 107.67 (dd, $^2J_{CF}$ = 23.8 Hz, $^4J_{CF}$ = 6.1 Hz), 106.87 (ddd, $^2J_{CF}$ = 21.7 Hz, $^3J_{CF}$ = 8.7 Hz, $^4J_{CF}$ = 2.6 Hz, 2C), 98.87 (t, $^4J_{CF}$ = 2.1 Hz, 1C), 72.77 (2C), 34.27 (1C), 31.78 (1C), 29.54 (1C), 28.21 (1C), 26.40 (1C), 22.74 (1C), 14.20 (1C)

IR v_{max} (cm^{-1}): 3110 (C-H stretch, sp^2 hybridised), 2924 (C-H stretch, sp^3 hybridised), 2110 (C-H bend, overtones), 1755 (C=O stretch, ester)

4'-(difluoro(3,4,5-trifluorophenoxy)methyl)-2,3',5',6-tetrafluoro-[1,1'-biphenyl]-4-yl 4-(1,3-dioxan-2-heptyl)-2,6-difluorobenzoate (SR-7-Re)

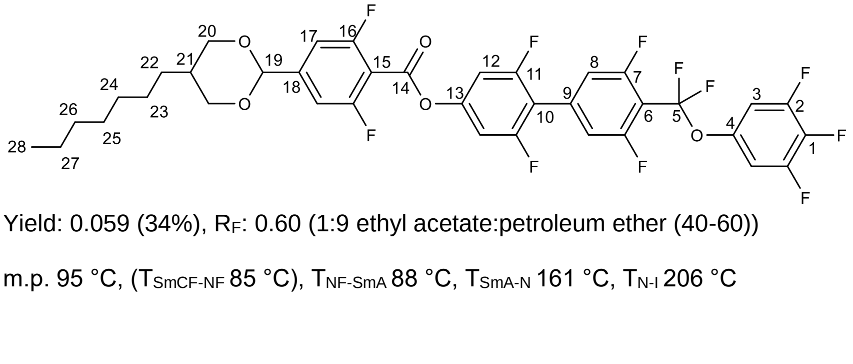

Yield: 0.059 (34%), R_F: 0.60 (1:9 ethyl acetate:petroleum ether (40-60))

m.p. 95 °C, ($T_{SmCF\text{-}NF}$ 85 °C), $T_{NF\text{-}SmA}$ 88 °C, $T_{SmA\text{-}N}$ 161 °C, $T_{N\text{-}I}$ 206 °C

^{1}H NMR (400 MHz, $CDCl_3$) δ ppm: 7.20 (d, $^{3}J_{HF}$ = 9.4 Hz, 2H, Ar-**H**, H_{17}), 7.16 (d, $^{3}J_{HF}$ = 10.5 Hz, 2H, Ar-**H**, H_{12}), 7.08 – 7.03 ($m_{overlapping\ d}$, 2H, Ar-**H**, H_8), 7.03 – 6.97 ($m_{overlapping\ d}$, 2H, Ar-**H**, H_3), 5.40 (s, 1H, R-CH-$(CH_2)_2$-$(O)_2$-**CH**-Ar, H_{19}), 4.29 – 4.22 (m, 2H, R-CH-(**CH_2**)$_2$-$(O)_2$-CH-Ar, H_{20}), 3.58 – 3.49 (m, 2H, R-CH-(**CH_2**)$_2$-$(O)_2$-CH-Ar, H_{20}), 2.19 – 2.05 (m, 1H, R-**CH**-$(CH_2)_2$-$(O)_2$-CH-Ar, H_{21}), 1.34 – 1.23 (m, 10H, CH_3-CH_2-**CH_2-CH_2-CH_2-CH_2-CH_2**-Dioxane, H_{22-26}), 1.15 – 1.06 (m, 2H, CH_3-**CH_2**-CH_2-CH_2-CH_2-CH_2-CH_2-Dioxane, H_{27}), 0.94 – 0.85 (m, 3H, **CH_3**-CH_2-CH_2-CH_2-CH_2-CH_2-CH_2-Dioxane, H_{28})

^{19}F NMR (proton decoupled) (376 MHz, $CDCl_3$) δ ppm: -61.97 (t, $^{4}J_{F\text{-}F}$ = 26.7 Hz, 2F, **F_5**), -108.14 (s, 2F, **F_{16}**), -110.48 (t, $^{4}J_{F\text{-}F}$ = 26.6 Hz, 2F, **F_7**), -111.70 (s, 2F, **F_{11}**), -132.42 (d, $^{3}J_{F\text{-}F}$ = 20.8 Hz, 2F, **F_2**), -163.04 (t, $^{3}J_{F\text{-}F}$ = 20.7 Hz, 1F, **F_1**)

^{13}C NMR (101 MHz, $CDCl_3$) δ ppm: 161.14 (dd, $^{1}J_{CF}$ = 258.9 Hz, $^{3}J_{CF}$ = 5.6 Hz, 2C), 161.30 – 158.46 ($m_{overlapping\ signals}$, 4C), 158.93 (s, apparent t, 1C), 151.44 (t, $^{3}J_{CF}$ = 14.3 Hz, 1C), 151.17 (ddd, $^{1}J_{CF}$ = 251.1 Hz, $^{2}J_{CF}$ = 10.8 Hz, $^{3}J_{CF}$ = 5.2 Hz, 2C), 146.19 (t, $^{3}J_{CF}$ = 9.9 Hz, 1C), 144.97 – 144.26 (m, 1C), 138.43 (dt, $^{1}J_{CF}$ = 250.3 Hz, $^{2}J_{CF}$ = 15.4 Hz, 1C), 134.41 (t, $^{3}J_{CF}$ = 11.5 Hz, 1C), 120.17 ($^{1}J_{CF}$ = 266.7 Hz, 1C), 114.90 (dd, $^{2}J_{CF}$ = 24.4 Hz, $^{4}J_{CF}$ = 2.4 Hz, 2C), 113.49 (t, $^{2}J_{CF}$ = 18.0 Hz, 1C), 110.54 (dd, $^{2}J_{CF}$ = 23.6 Hz, $^{4}J_{CF}$ = 3.4 Hz, 2C), 109.87 (t, $^{2}J_{CF}$ = 14.4 Hz, 1C), 109.04 (t, $^{2}J_{CF}$ = 16.7 Hz, 1C), 107.66 (dd, $^{2}J_{CF}$ = 23.9 Hz, $^{4}J_{CF}$ = 6.4 Hz, 2C), 106.85 (ddd, $^{2}J_{CF}$ = 21.7 Hz, $^{3}J_{CF}$ = 8.5 Hz, $^{4}J_{CF}$ = 2.6 Hz, 2C), 98.87 (t, $^{4}J_{CF}$ = 2.2 Hz, 1C), 72.77 (2C), 34.27 (1C), 31.93 (1C), 29.84 (1C), 29.24 (1C), 28.21 (1C), 26.44 (1C), 22.78 (1C), 14.22 (1C)

IR ν_{max} (cm^{-1}): 3109 (C-H stretch, sp^2 hybridised), 2922 (C-H stretch, sp^3 hybridised), 1981 (C-H bend, overtones), 1757 (C=O stretch, ester)

NMR Spectra SR-*n*-Re

SR-1-Re

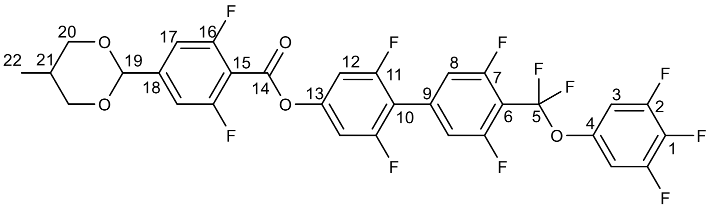

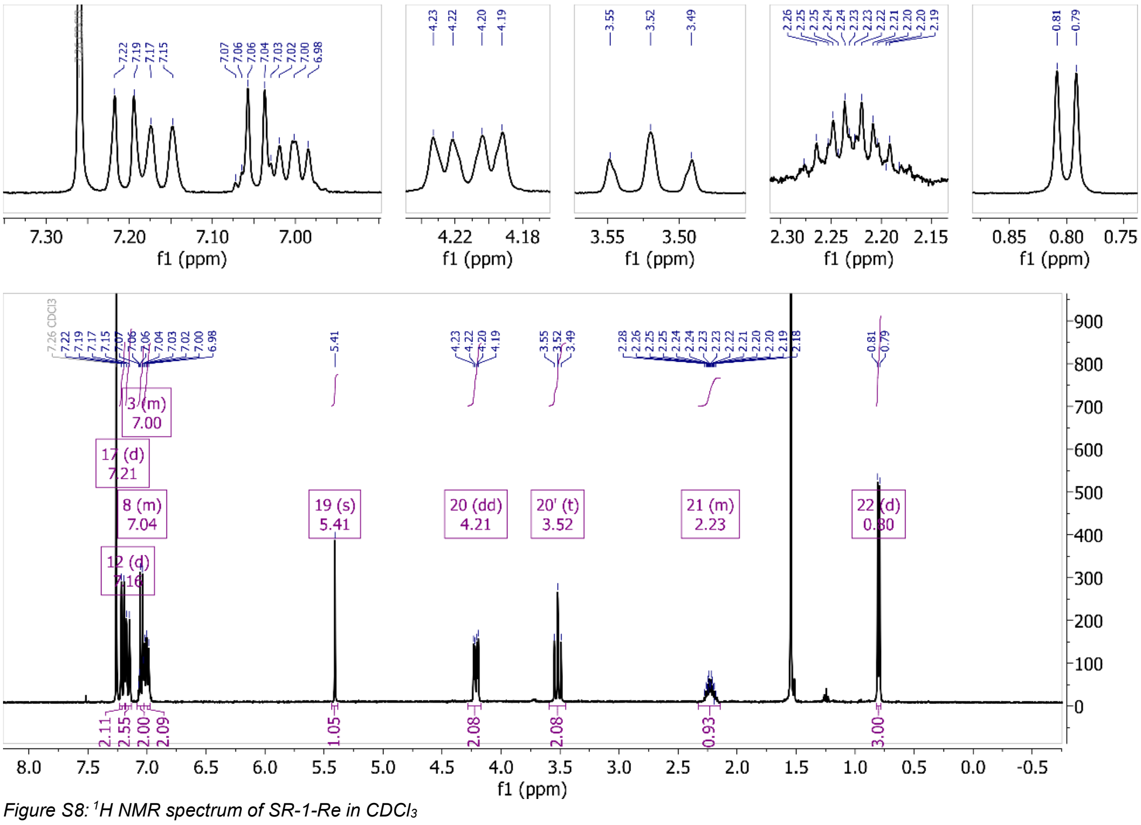

Figure S8: ^{1}H NMR spectrum of SR-1-Re in $CDCl_3$

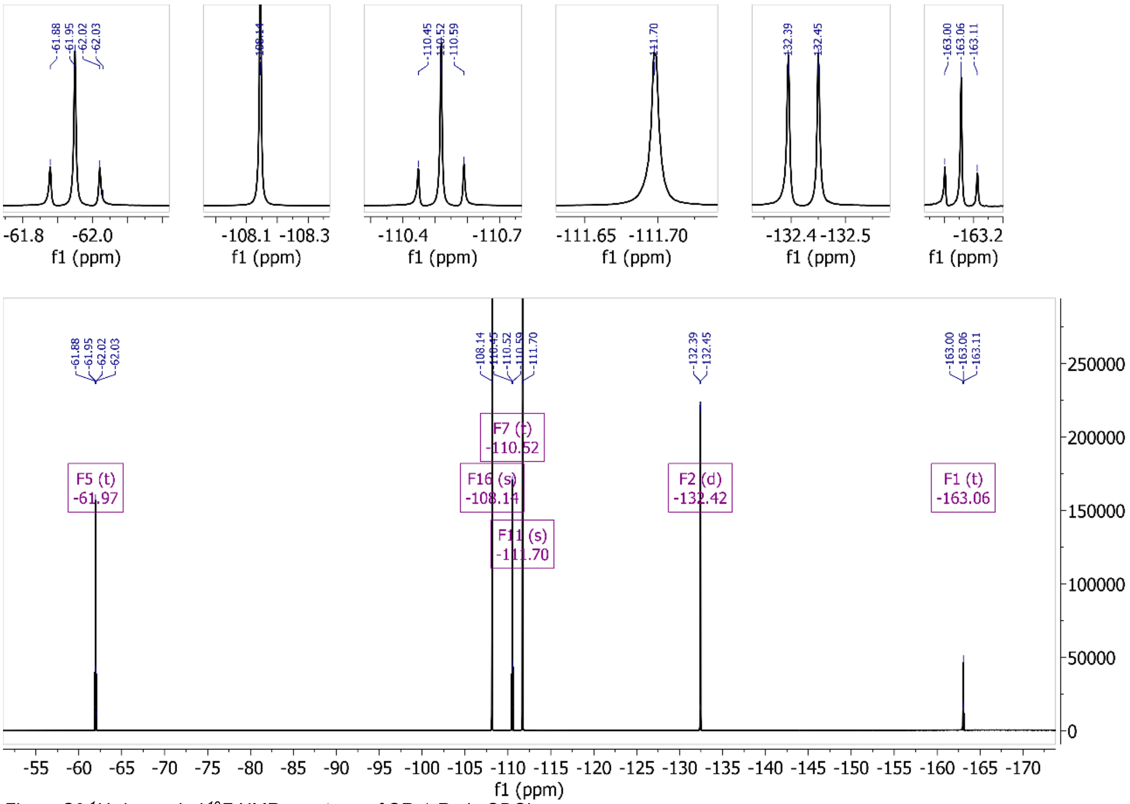

Figure S9: ^{1}H-decoupled ^{19}F NMR spectrum of SR-1-Re in $CDCl_3$

SR-2-Re

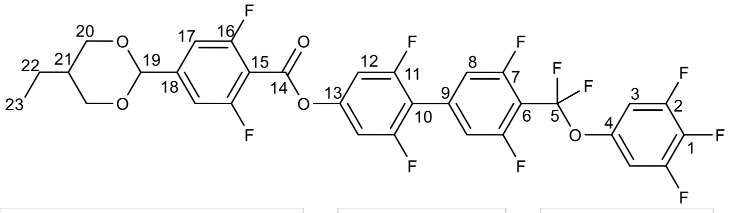

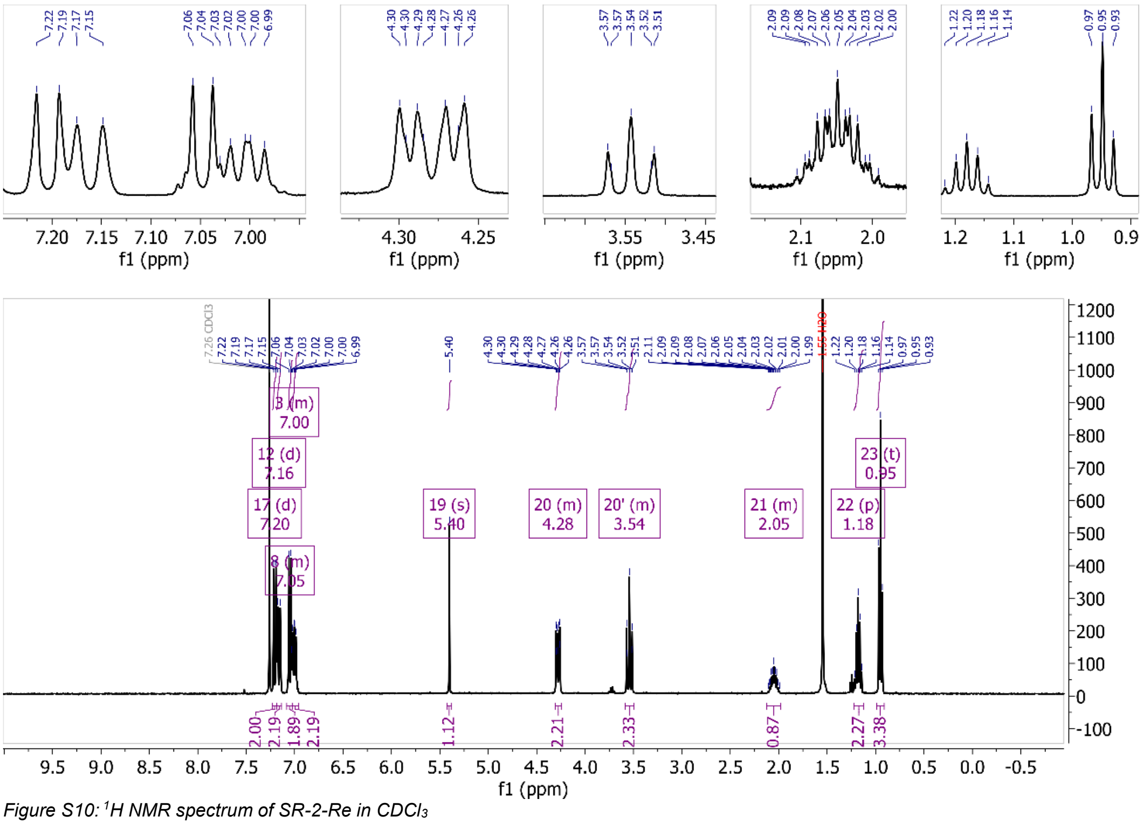

Figure S10: ^{1}H NMR spectrum of SR-2-Re in CDCl₃

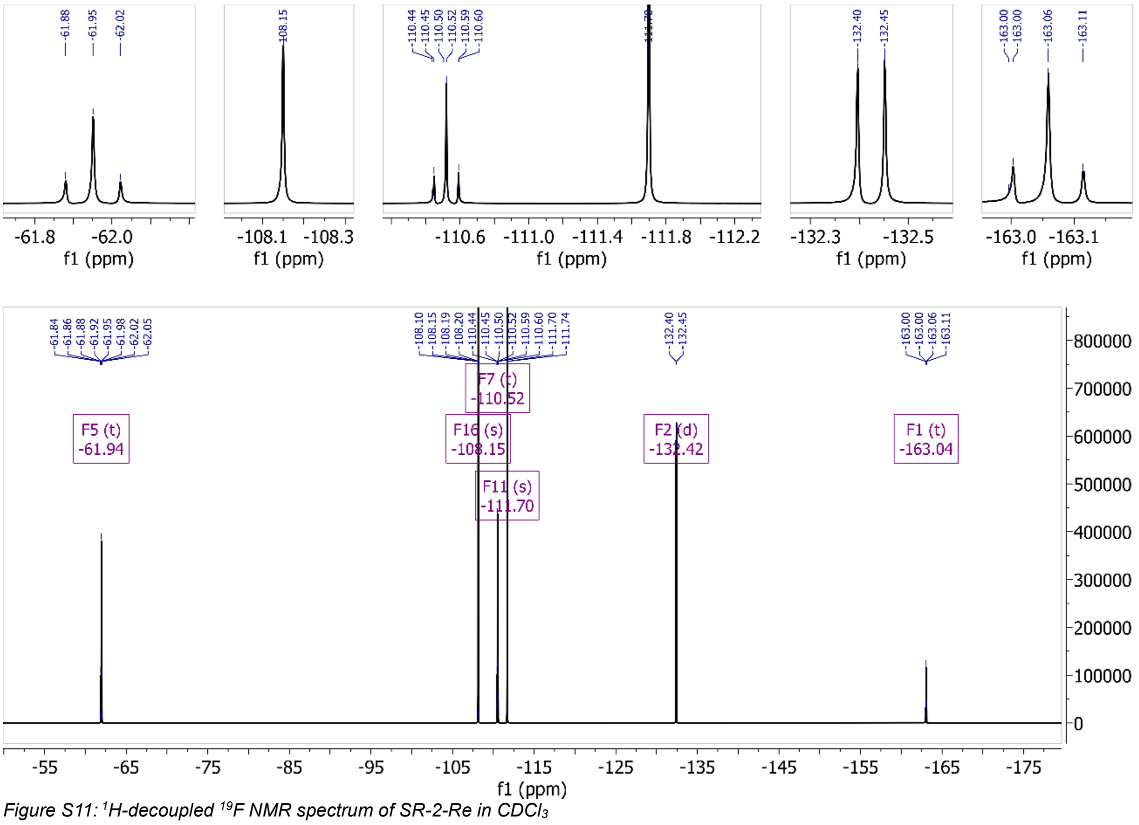

Figure S11: ^{1}H-decoupled ^{19}F NMR spectrum of SR-2-Re in CDCl₃

SR-3-Re

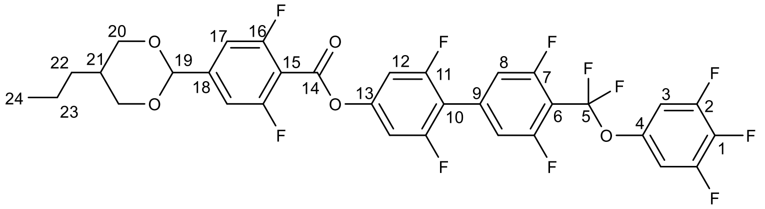

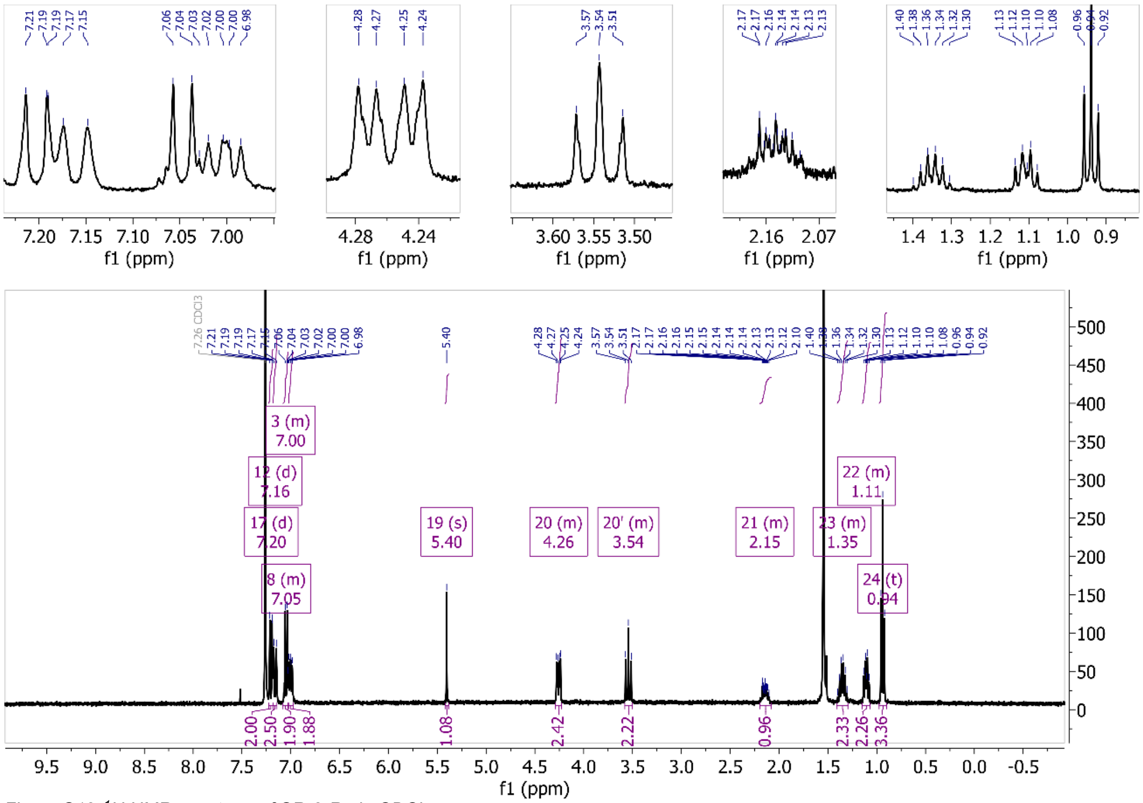

Figure S12: ^{1}H NMR spectrum of SR-3-Re in $CDCl_3$

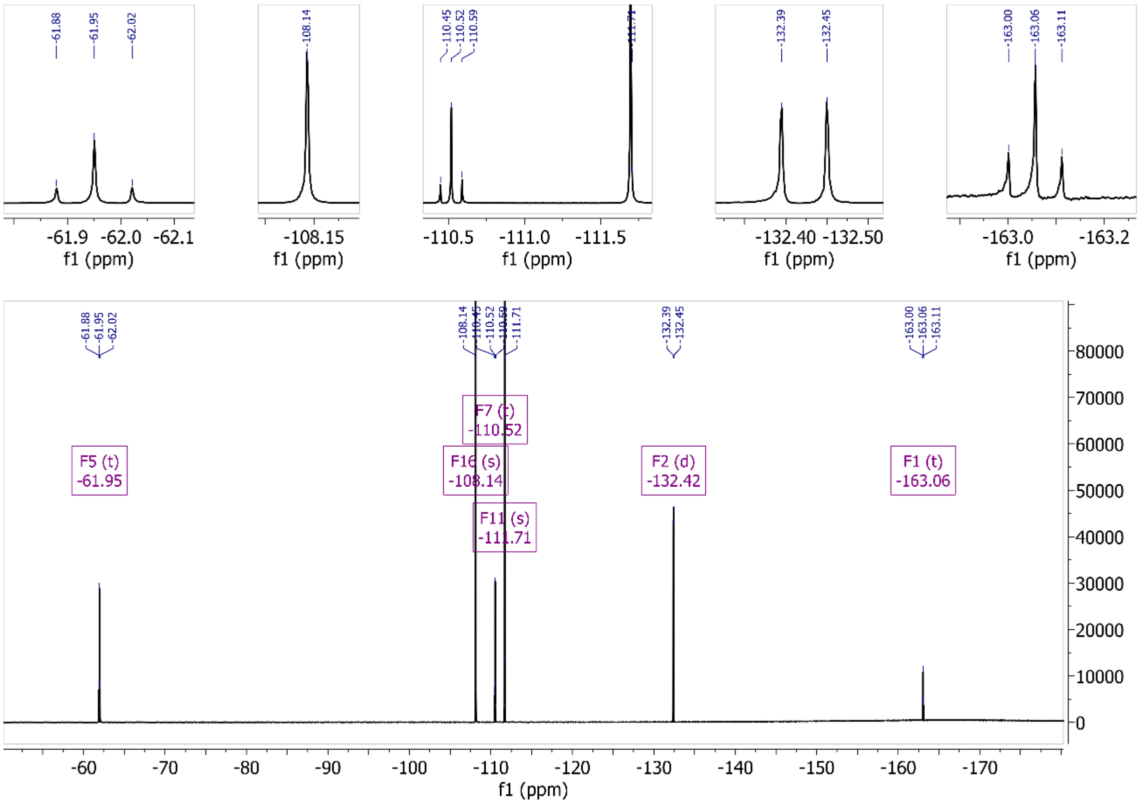

Figure S13: ^{1}H-decoupled ^{19}F NMR spectrum of SR-3-Re in $CDCl_3$

SR-4-Re

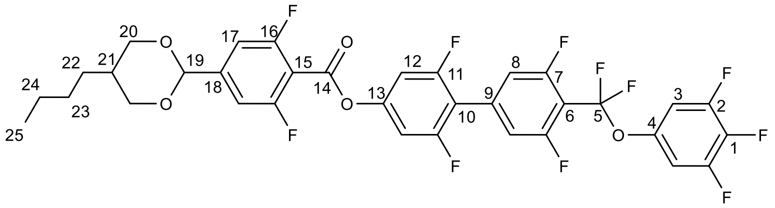

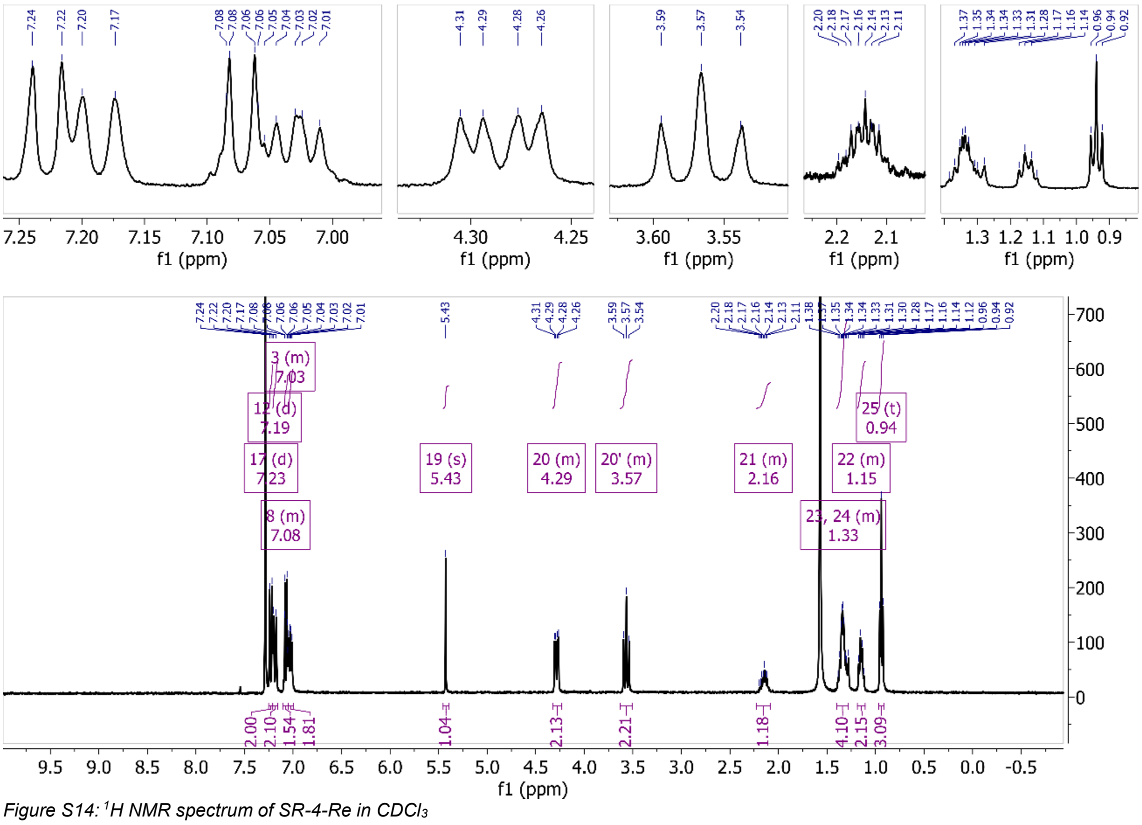

Figure S14: ^{1}H NMR spectrum of SR-4-Re in $CDCl_3$

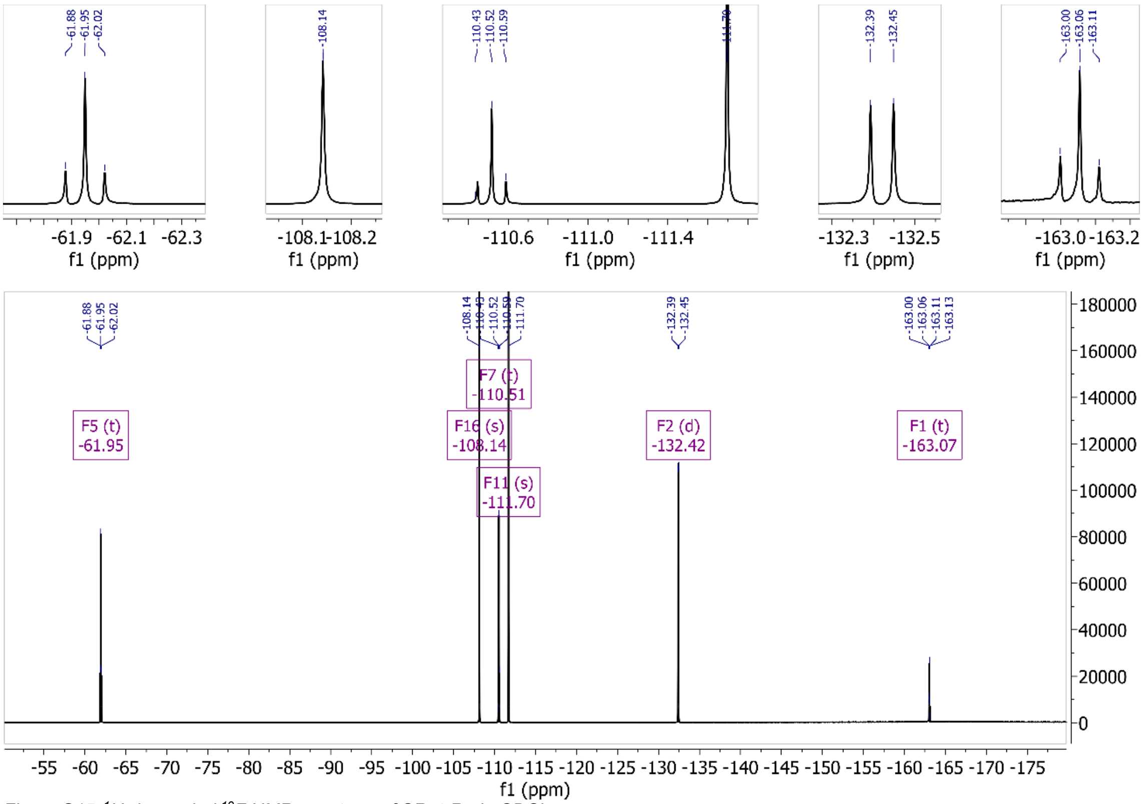

Figure S15: ^{1}H-decoupled ^{19}F NMR spectrum of SR-4-Re in $CDCl_3$

SR-5-Re

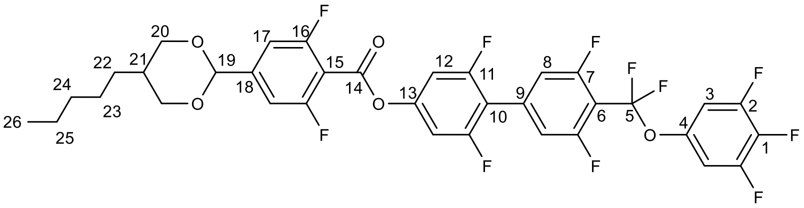

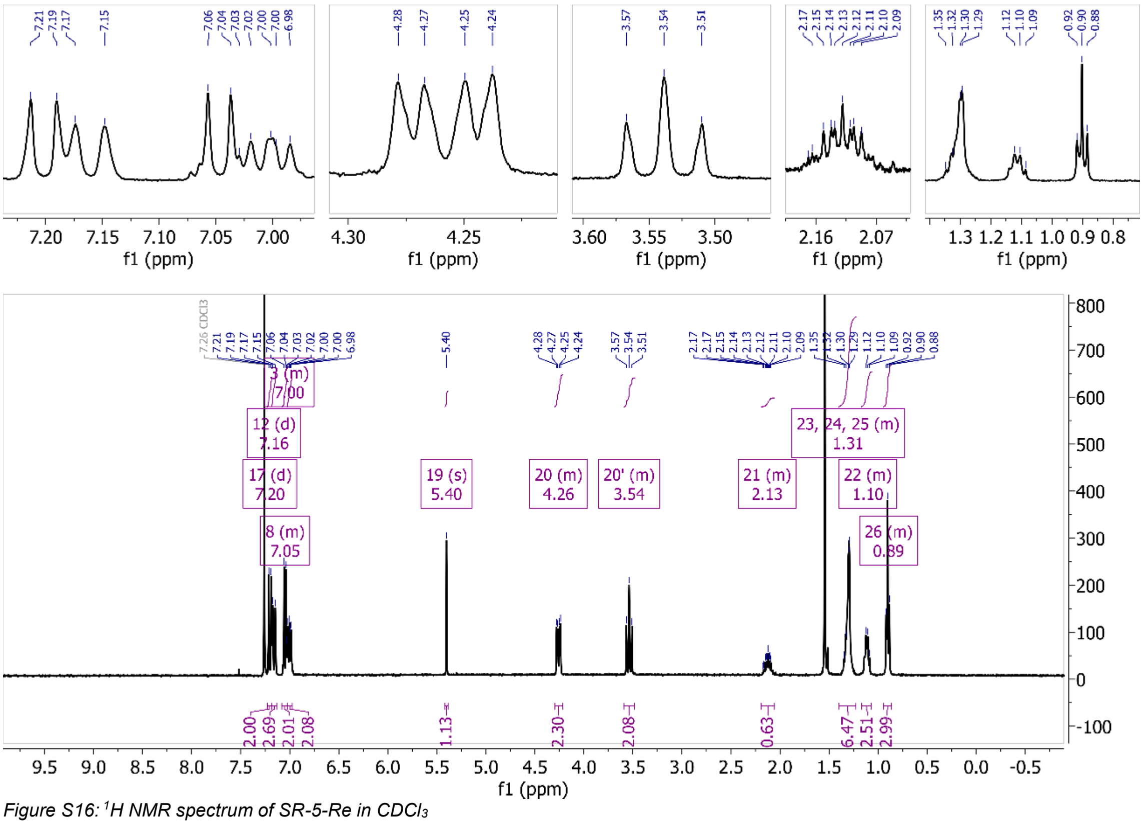

Figure S16: ¹H NMR spectrum of SR-5-Re in $CDCl_3$

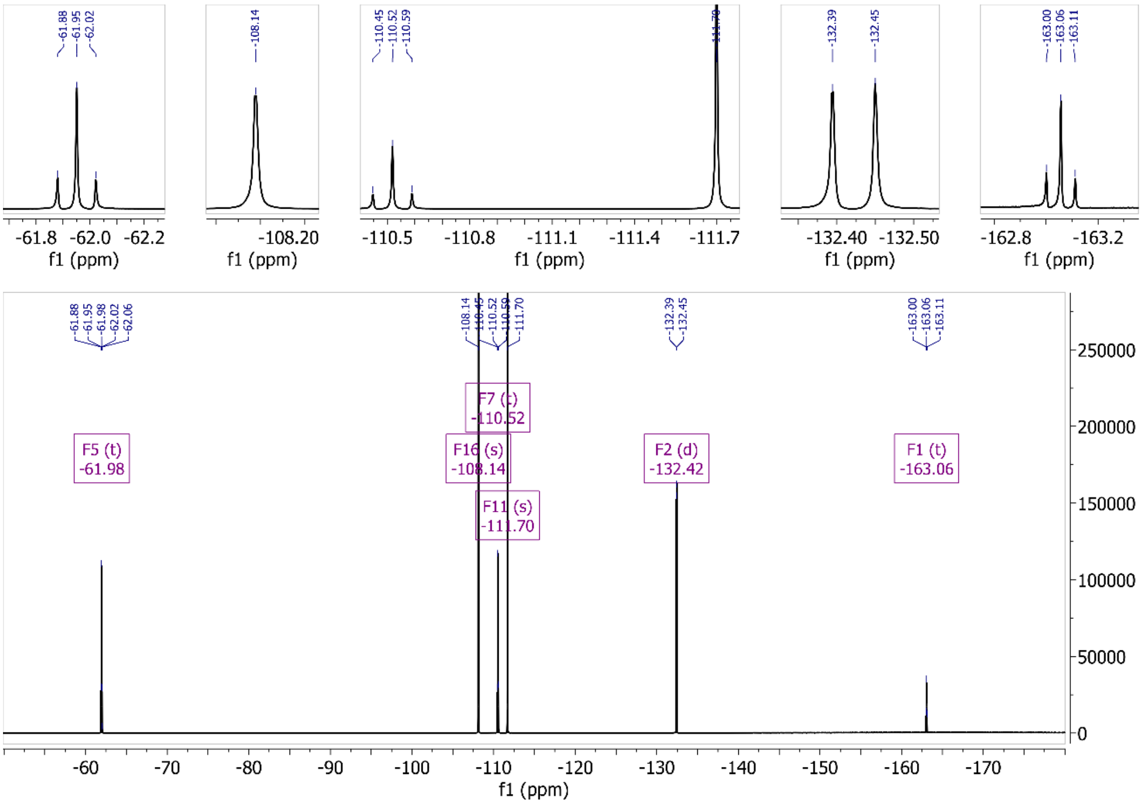

Figure S17: ¹H-decoupled ¹⁹F NMR spectrum of SR-5-Re in $CDCl_3$

SR-6-Re

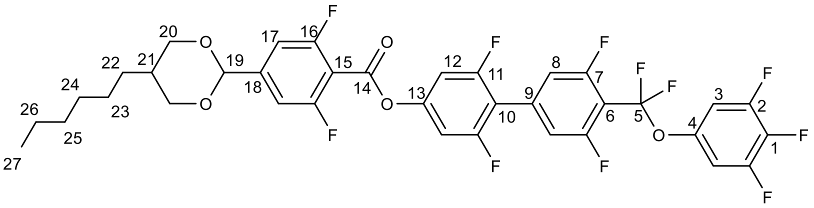

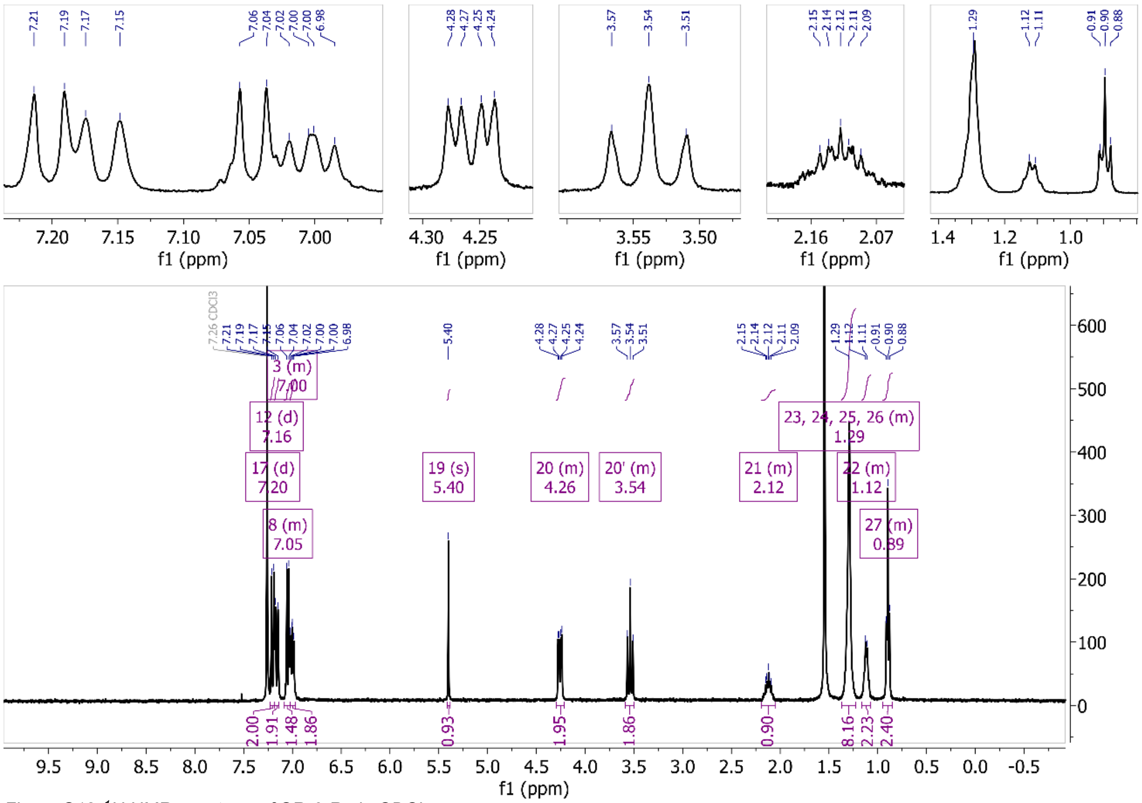

Figure S18: ^{1}H NMR spectrum of SR-6-Re in $CDCl_3$

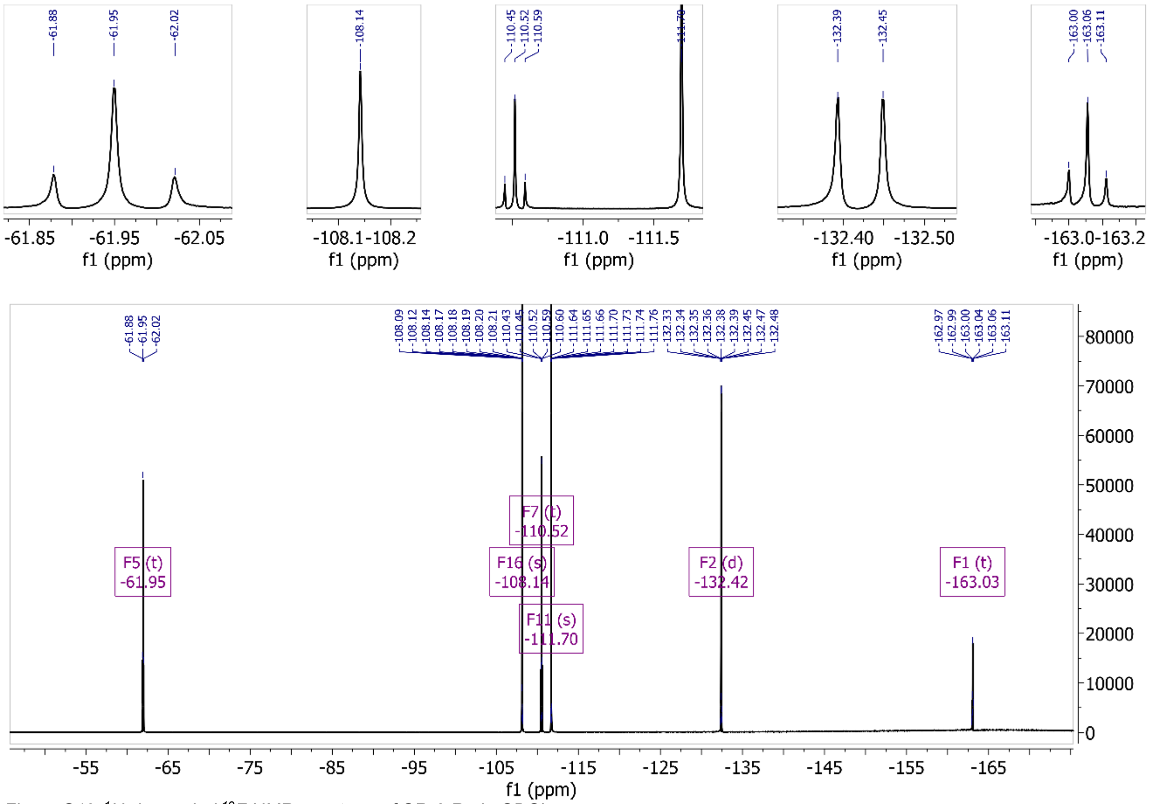

Figure S19: ^{1}H-decoupled ^{19}F NMR spectrum of SR-6-Re in $CDCl_3$

SR-7-Re

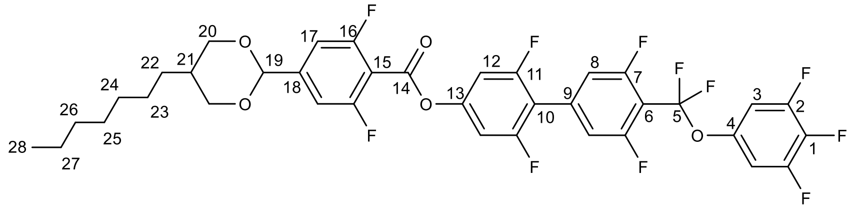

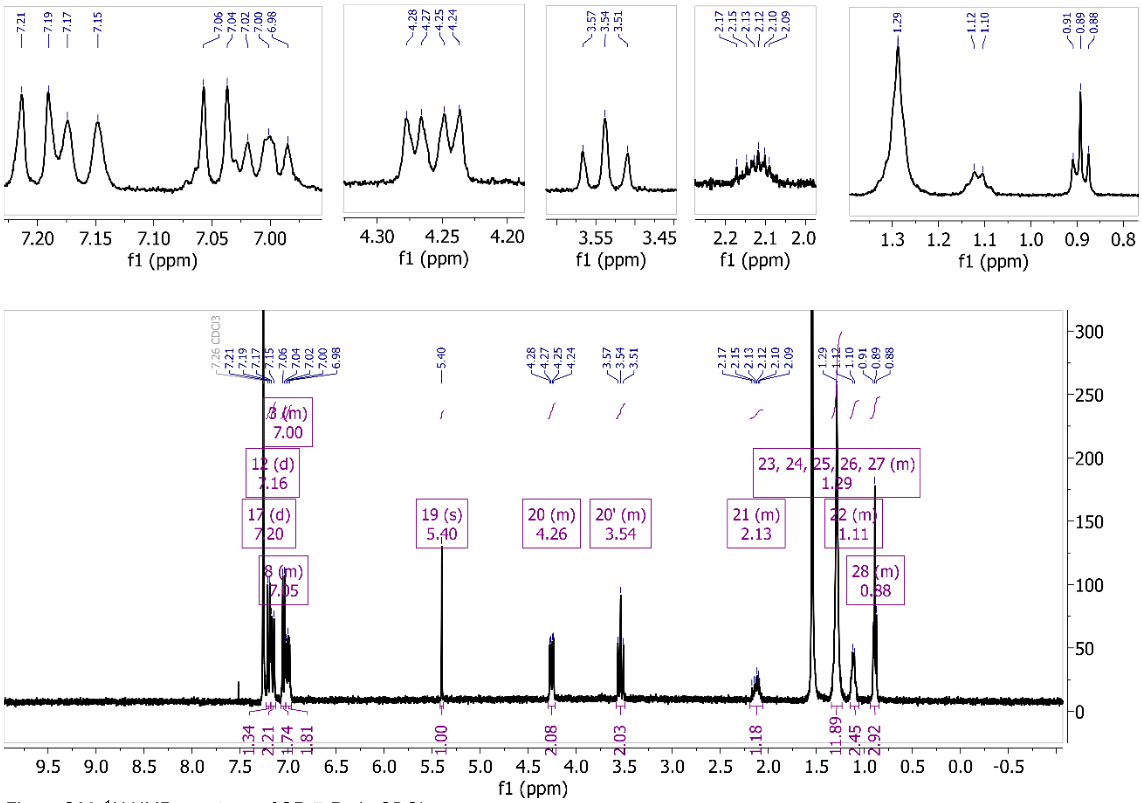

Figure S20: ¹H NMR spectrum of SR-7-Re in $CDCl_3$

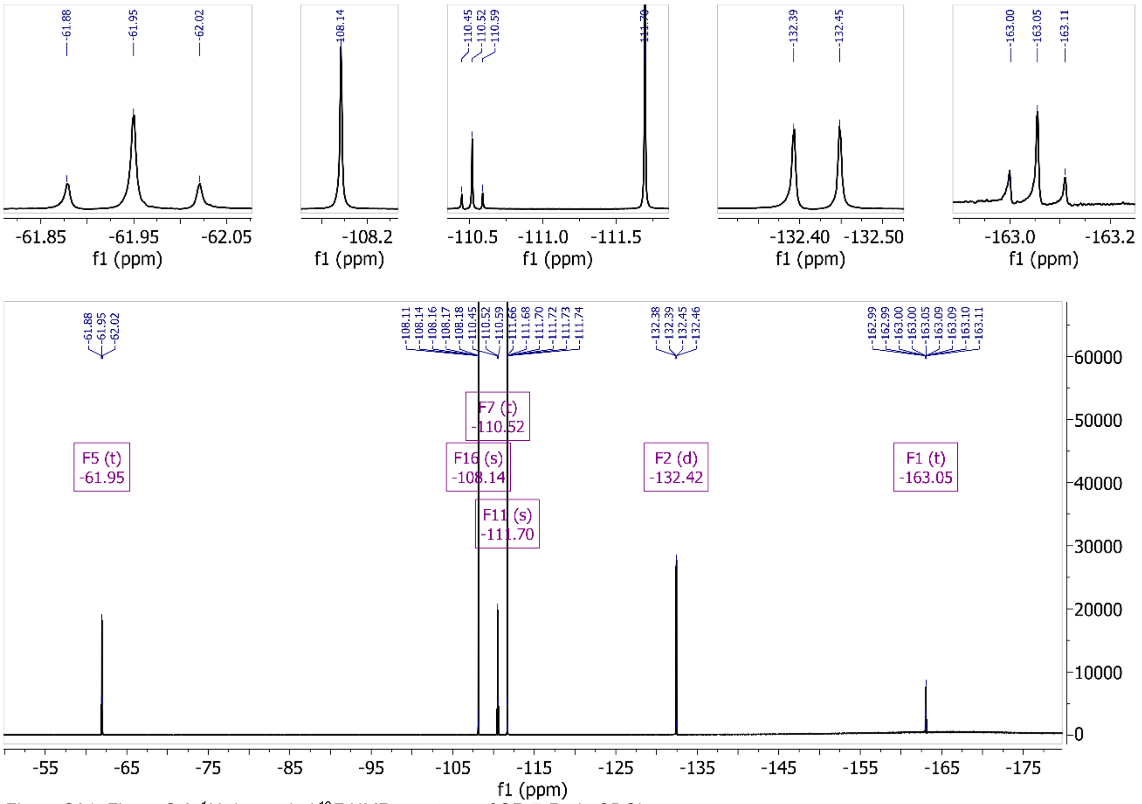

Figure S21: Figure S 2: ¹H-decoupled ¹⁹F NMR spectrum of SR-7-Re in $CDCl_3$

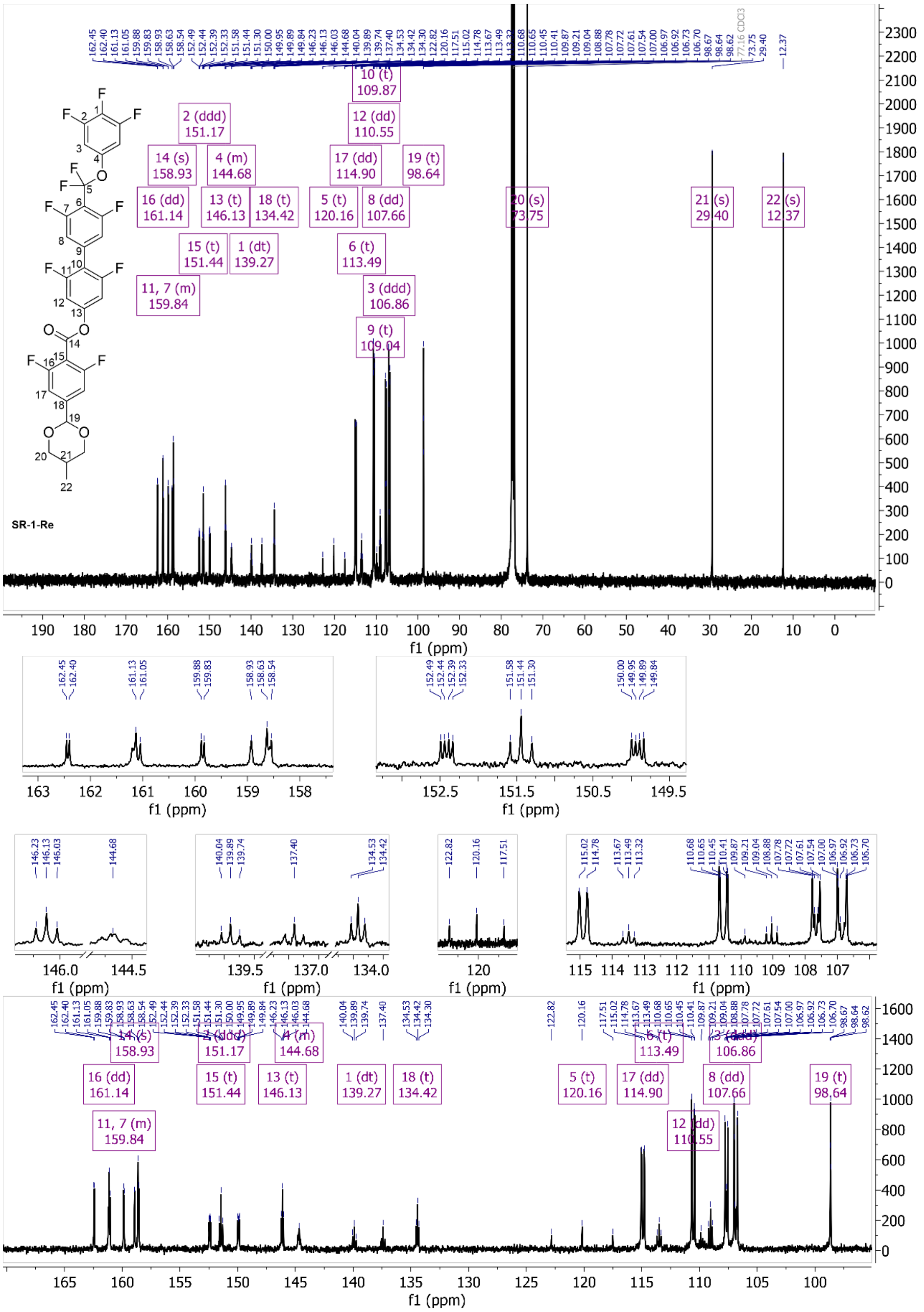

Figure S22: Representative assigned ^{13}C NMR spectrum of SR-1-Re in $CDCl_3$. Top: Full spectrum; bottom: enlarged aromatic region

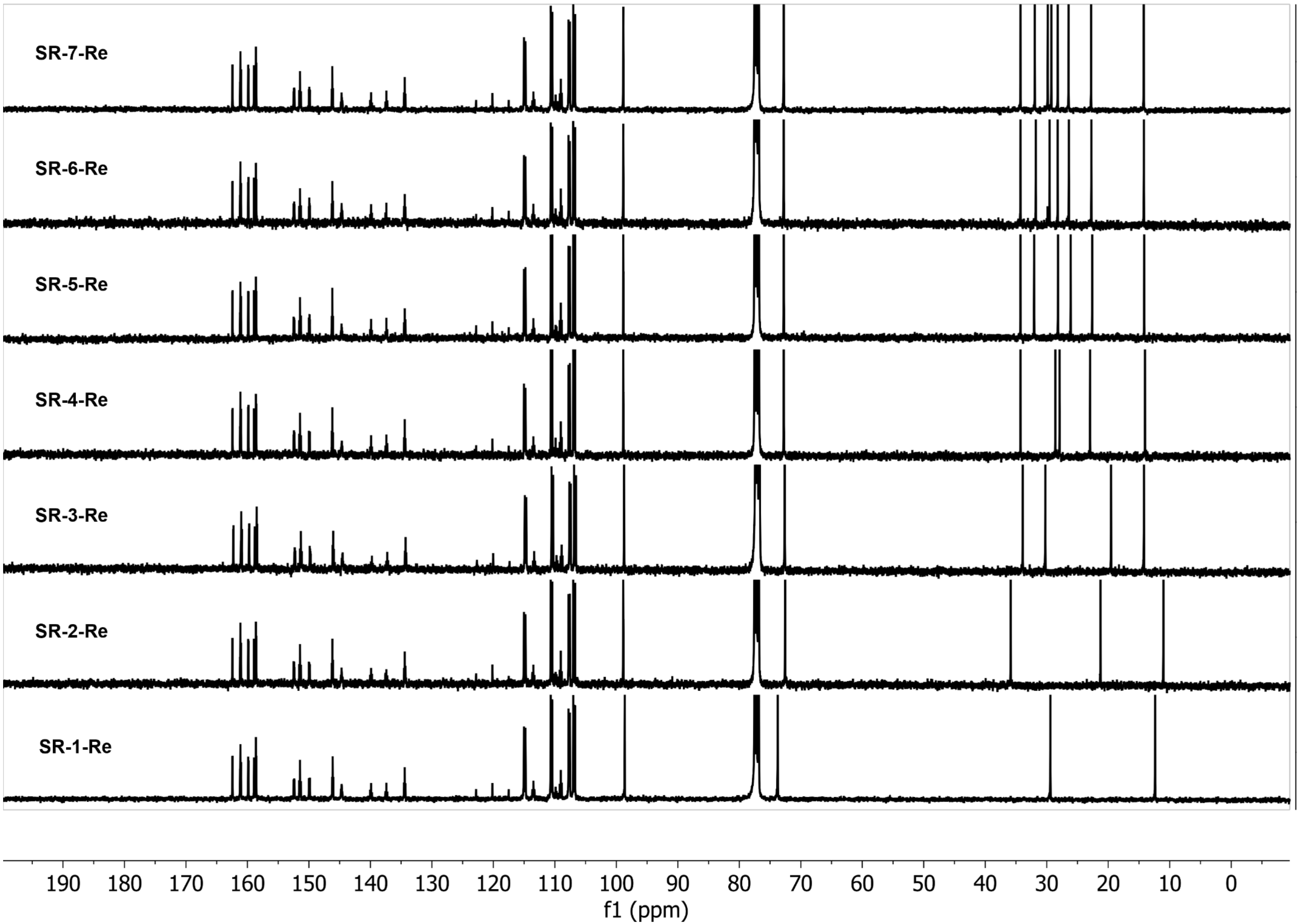

*Figure S23: Stacked ^{13}C NMR spectra for series SR-*n*-Re.*

3.2 Synthesis of GS-*n*-Re series

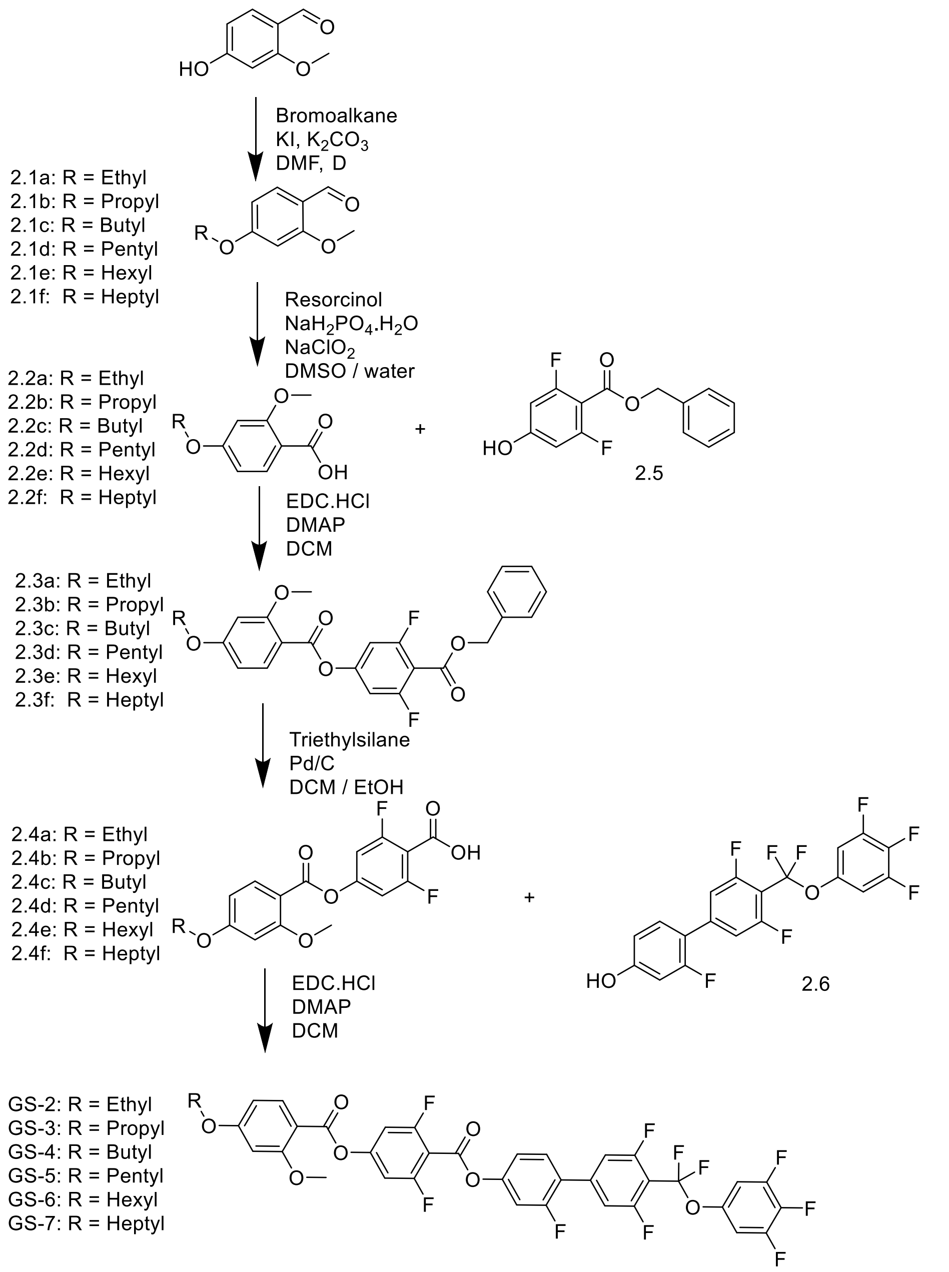

Scheme S2. The synthetic route to materials GS-n. Intermediates 1a[30], 2a[30], 5[31], and 6[21], and mesogens GS-1[31] have been reported previously.

General Methods:

2A. Williamson ether

Potassium carbonate, potassium iodide, and 4-hydroxy-2-methoxybenzaldehyde were added to dry DMF under an argon atmosphere and stirred for 10 minutes. The appropriate alkyl bromide was added and the reaction mixture heated to 70 °C overnight. The mixture was cooled, added to 500 ml of water, and stirred until an orange precipitate formed. This was collected by vacuum filtration and the crude product was carried forward without further purification.

2B. Oxidation

The aldehyde and resorcinol were dissolved in DMSO. Sodium chlorite and sodium dihydrogen phosphate were dissolved in water, and this was added carefully to the organics and the reaction left stirring overnight. The reaction mixture was diluted with 100 ml water, acidified, and the resulting precipitate collected by vacuum filtration.

2C. Esterification

The appropriate benzoic acid and EDC.HCl were dissolved in DCM and stirred for 10 minutes. The corresponding phenol and 4-dimethylaminopyridine (DMAP) were added and the reaction was left stirring at room temperature overnight. The reaction was washed 3x with water and the solvent removed *in vacuo*. The crude product was recrystallised from ethanol to yield the product as a white solid.

2D. Deprotection

Under an argon atmosphere triethylsilane was added dropwise to a stirred solution of 3-x and 5 % Pd/C in a 1:1 mix of ethanol and DCM. The reaction was stirred for 15 minutes after addition was complete, then filtered through celite and the solvent removed *in vacuo*. The crude product was washed with hexane to yield the product as a white powder.

4-propoxy-2-methoxybenzaldehyde 2.1b.

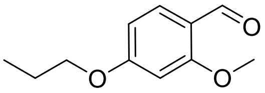

Method 2A

4-hydroxy-2-methoxybenzaldehyde	2.067 g,	13.6 mmol,	1 eq.
1-bromopropane	1.4 ml,	15 mmol,	1.1 eq.
K_2CO_3	3.776 g,	27 mmol,	2 eq.
KI	600 mg,	3.6 mmol,	0.3 eq.
DMF	30 ml		

20 hrs
Yield 2.89 g.
^{1}H NMR (400 MHz, $CDCl_3$) δ = 10.27 (s, 1H), 7.78 (d, *J*=8.6, 1H), 6.52 (dd, *J*=8.6, ^{4}J=2.2, 1H), 6.43 (d, ^{4}J=2.2, 1H), 3.98 (t, *J*=6.6, 2H), 3.88 (s, 3H), 1.89 – 1.75 (m, 2H), 1.04 (t, *J*=7.4, 3H).

4-butyloxy-2-methoxybenzaldehyde 2.1c.

Method 2A

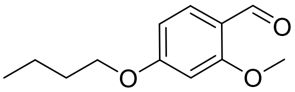

4-hydroxy-2-methoxybenzaldehyde	2.063 g,	13.6 mmol,	1 eq.
1-bromobutane	1.7 ml,	15 mmol,	1.1 eq.
K_2CO_3	3.998 g,	29 mmol,	2.1 eq.
KI	360 mg,	2.1 mmol,	0.2 eq.
DMF	30 ml		

15 hrs
Yield 2.82 g.
^{1}H NMR (400 MHz, $CDCl_3$) δ = 10.28 (s, 1H), 7.79 (d, *J*=8.6, 1H), 6.53 (dd, *J*=8.6, ^{4}J=2.2, 1H), 6.44 (d, ^{4}J=2.2, 1H), 4.03 (t, *J*=6.5, 2H), 3.90 (s, 2H), 1.85 – 1.73 (m, 2H), 1.57 – 1.43 (m, 2H), 0.99 (t, *J*=7.4, 3H).

4-pentyloxy-2-methoxybenzaldehyde 2.1d.

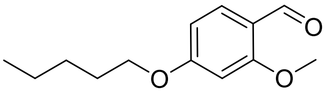

Method 2A

4-hydroxy-2-methoxybenzaldehyde	2.085 g,	13.7 mmol,	1 eq.
1-bromopentane	1.9 ml,	15 mmol,	1.1 eq.
K_2CO_3	3.785 g,	27 mmol,	2 eq.
KI	200 mg,	1.2 mmol,	0.1 eq.
DMF	30 ml		

16 hours
Yield 2.92 g.
^{1}H NMR (400 MHz, $CDCl_3$) δ = 10.28 (s, 1H), 7.79 (d, *J*=8.7, 1H), 7.26 (s, 1H), 6.53 (dd, *J*=8.7, ^{4}J=2.1, 1H), 6.44 (d, ^{4}J=2.1, 1H), 4.02 (t, *J*=6.5, 2H), 3.90 (s, 2H), 1.81 (p, *J*=6.5, 2H), 1.51 – 1.32 (m, 4H), 0.94 (t, *J*=7.0, 3H).

4-hexyloxy-2-methoxybenzaldehyde 2.1e.

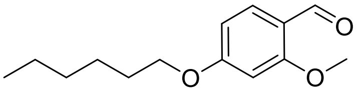

Method 2A

4-hydroxy-2-methoxybenzaldehyde	2.072 g,	13.6 mmol,	1 eq.
1-bromohexane	2.1 ml,	15 mmol,	1.1 eq.
K_2CO_3	3.777 g,	27 mmol,	2 eq.
KI	150 mg,	1.2 mmol,	0.06 eq.
DMF	30 ml		

15 hours
Yield 3.11 g.
^{1}H NMR (400 MHz, $CDCl_3$) δ = 10.31 (s, 1H), 7.82 (d, *J*=8.7, 1H), 6.56 (dd, *J*=8.7, ^{4}J=2.2, 1H), 6.46 (d, ^{4}J=2.2, 1H), 4.05 (t, *J*=6.5, 2H), 3.92 (s, 3H), 1.83 (p, *J*=6.7, 2H), 1.55 – 1.44 (m, 2H), 1.44 – 1.33 (m, 4H), 0.98 – 0.90 (m, 3H).

4-heptyloxy-2-methoxybenzaldehyde 2.1f.

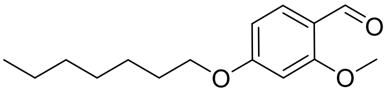

Method 2A

4-hydroxy-2-methoxybenzaldehyde	2.072 g,	13.6 mmol,	1 eq.
1-bromoheptane	2.4 ml,	15 mmol,	1.1 eq.
K_2CO_3	3.774 g,	27 mmol,	2 eq.
KI	100 mg,	1.2 mmol,	0.04 eq.
DMF	30 ml		

15 hours
Yield 3.16 g.
^{1}H NMR (400 MHz, $CDCl_3$) δ = 10.31 (s, 1H), 7.82 (d, *J*=8.7, 1H), 6.56 (dd, *J*=8.7, ^{4}J=2.2, 1H), 6.47 (d, ^{4}J=2.2, 1H), 4.05 (t, *J*=6.5, 2H), 3.92 (s, 3H), 1.89 – 1.75 (m, 2H), 1.55 – 1.39 (m, 2H), 1.42 – 1.29 (m, *J*=3.5, 6H), 0.96 – 0.88 (m, 3H).

4-propoxy-2-methoxybenzoic acid 2.2b.

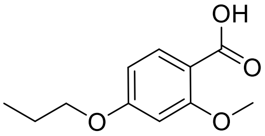

Method 2B

Aldehyde 2.1c	2.546 g,	11.4 mmol,	1 eq.
Resorcinol	4.751 g,	43 mmol,	4 eq.
$NaClO_2$	5.140 g,	57 mmol,	5 eq.
$NaH_2PO_4.H_2O$	6.860 g,	50 mmol,	4.4 eq.
DMSO	60 ml		
Water	45 ml		

23 hrs
Yield 1.68 g.
^{1}H NMR (400 MHz, $CDCl_3$) δ = 8.11 (d, *J*=8.8, 1H), 6.63 (dd, *J*=8.8, ^{4}J=2.2, 1H), 6.53 (d, ^{4}J=2.2, 1H), 4.04 (s, 3H), 3.99 (t, *J*=6.5, 2H), 1.90 – 1.77 (m, 2H), 1.05 (t, *J*=7.4, 3H).

4-Butyloxy-2-methoxybenzoic acid 2.2c.

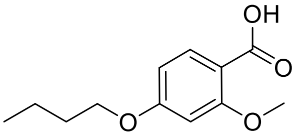

Method 2B

Aldehyde 21b	2.600 g,	12.5 mmol,	1 eq.
Resorcinol	3.975 g,	36 mmol,	3 eq.
$NaClO_2$	4.369 g,	48 mmol,	4 eq.
$NaH_2PO_4.H_2O$	5.810 g,	42 mmol,	3.3 eq.
DMSO	50 ml		
Water	40 ml		

23 hrs
Yield 2.84 g.
^{1}H NMR (400 MHz, $CDCl_3$) δ = 8.12 (d, *J*=8.8, 1H), 6.63 (dd, *J*=8.8, ^{4}J=2.2, 1H), 6.53 (d, ^{4}J=2.2, 1H), 4.04 (s, 3H), 4.03 (t, *J*=6.5, 2H), 1.85 – 1.73 (m, 2H), 1.57 – 1.43 (m, 2H), 0.99 (t, *J*=7.4, 3H).

4-Pentyloxy-2-methoxybenzoic acid 2.2d.

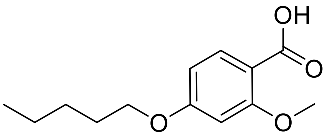

Method 2B

Aldehyde 2.1d	2.90 g,	13 mmol,	1 eq.
Resorcinol	5.10 g,	46 mmol,	3.5 eq.
$NaClO_2$	5.62 g,	62 mmol,	4.7 eq.
$NaH_2PO_4.H_2O$	7.49 g,	55 mmol,	4.2 eq.
DMSO	60 ml		
Water	45 ml		

21 hrs
Yield 3.24 g.
^{1}H NMR (400 MHz, $CDCl_3$) δ = 8.14 (d, *J*=8.8, 1H), 6.66 (dd, *J*=8.8, ^4J=2.2, 1H), 6.55 (d, ^4J=2.2, 1H), 4.06 (overlapping singlet and triplet, 5H), 1.89 – 1.78 (m, 2H), 1.54 – 1.35 (m, 4H), 0.97 (t, *J*=7.0, 3H).

4-Hexyloxy-2-methoxybenzoic acid 2.2e.

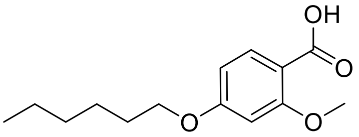

Method 2B

Aldehyde 2.1e	3.109 g,	13 mmol,	1 eq.
Resorcinol	2.17 g,	20 mmol,	1.5 eq.
$NaClO_2$	4.79 g,	53 mmol,	4 eq.
$NaH_2PO_4.H_2O$	6.50 g,	47 mmol,	3.6 eq.
DMSO	60 ml		
Water	40 ml		

50 hrs
Yield 3.98 g.
^{1}H NMR (400 MHz, $CDCl_3$) δ = 8.14 (d, *J*=8.8, 1H), 6.66 (dd, *J*=8.8, ^4J=2.2, 1H), 6.55 (d, ^4J=2.2, 1H), 4.06 (overlapping singlet and triplet, 5H), 1.88 – 1.77 (m, 2H), 1.55 – 1.43 (m, 2H), 1.42 – 1.33 (m, 4H), 0.98 – 0.90 (m, 3H).

4-Heptyloxy-2-methoxybenzoic acid 2.2f.

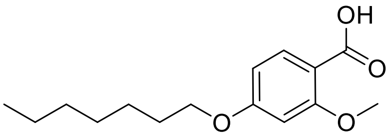

Method 2B

Aldehyde 2.1f	3.168 g,	13 mmol,	1 eq.
Resorcinol	2.609 g,	24 mmol,	1.8 eq.
$NaClO_2$	4.963 g,	55 mmol,	4.2 eq.
$NaH_2PO_4.H_2O$	6.370 g,	46 mmol,	3.5 eq.
DMSO	60 ml		
Water	40 ml		

50 hrs
Yield 2.80 g.
^{1}H NMR (400 MHz, $CDCl_3$) δ = 8.15 (d, *J*=8.8, 1H), 6.66 (dd, *J*=8.8, ^4J=2.3, 1H), 6.55 (d, ^4J=2.3, 1H), 4.06 (overlapping singlet and triplet, 5H), 1.95 – 1.77 (m, 2H), 1.54 – 1.39 (m, 2H), 1.36 – 1.29 (m, 6H), 0.96 – 0.88 (m, 3H).

4-((benzyloxy)carbonyl)-3,5-difluorophenyl-4'-ethoxy-2'-methoxybenzoate 2.3a

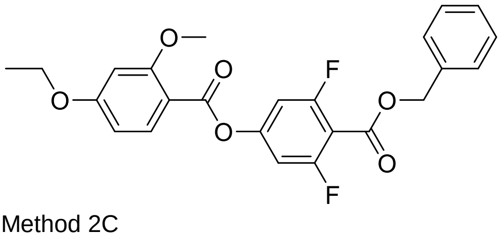

Method 2C

Benzoic acid 2.2a	350 mg,	1.8 mmol,	1.1 eq.
Benzyl-4-hydroxy-2,6-difluorobenzoate	427 mg,	1.6 mmol,	1 eq.

EDC.HCl	636 mg,	3.2 mmol,	2 eq.
DMAP	23 mg,	0.18 mmol,	0.1 eq.
DCM	10 ml		

18 hrs
Yield 170 mg.
^{1}H NMR (400 MHz, $CDCl_3$) δ = 8.05 – 7.97 (m, 1H), 7.47 – 7.32 (m, 5H), 6.91 (d, J_{HF}=9.6, 2H), 6.54 (overlapping signals, 2H), 5.40 (s, 2H), 4.17 – 4.07 (m, 2H), 3.91 (s, 3H), 1.51 – 1.42 (m, 3H).

4-((benzyloxy)carbonyl)-3,5-difluorophenyl-4'-propoxy-2'-methoxybenzoate 2.3b

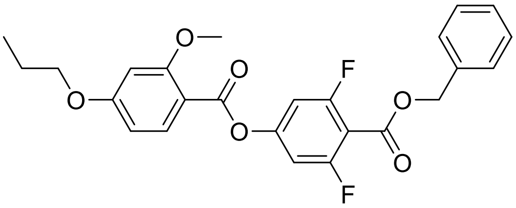

Method 2C

Benzoic acid 2.2b	962 mg,	2.3 mmol,	2.3 eq.
Benzyl-4-hydroxy-2,6-difluorobenzoate	264 mg,	1 mmol,	1 eq.
EDC.HCl	346 mg,	1.8 mmol,	1.8 eq.
DMAP	10 mg,	0.1 mmol,	0.1 eq.
DCM	10 ml		

18 hrs
Yield 160 mg.
^{1}H NMR (400 MHz, $CDCl_3$) δ = 8.01 (d, J=8.6, 1H), 7.48 – 7.32 (m, 5H), 6.91 (d, J_{HF}=8.7, 1H), 6.58 – 6.46 (overlapping, 2H), 5.40 (s, 2H), 4.01 (t, J=6.7, 2H), 3.92 (s, 3H), 1.92 – 1.80 (m, 2H), 1.07 (t, J=7.4, 3H).

4-((benzyloxy)carbonyl)-3,5-difluorophenyl-4'-butoxy-2'-methoxybenzoate 2.3c

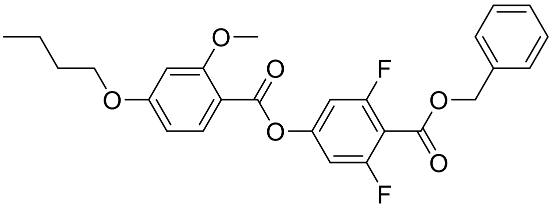

Method 2C

Benzoic acid 2.2c	436 mg,	2 mmol,	1 eq.
Benzyl-4-hydroxy-2,6-difluorobenzoate	264 mg,	2 mmol,	1 eq.
EDC.HCl	846 mg,	4.3 mmol,	2.1 eq.
DMAP	22 mg,	0.2 mmol,	0.1 eq.
DCM	10 ml		

18 hrs
Yield 160 mg.
^{1}H NMR (400 MHz, $CDCl_3$) δ = 8.00 (d, J=8.8, 1H), 7.48 – 7.31 (m, 5H), 6.90 (d, J_{HF}=8.8, 2H), 6.57 – 6.49 (m, 2H), 5.39 (s, 2H), 4.04 (t, J=6.5, 2H), 3.92 (s, 3H), 1.86 – 1.74 (m, 2H), 1.51 (h, J=7.4, 2H), 0.99 (t, J=7.4, 3H).

4-((benzyloxy)carbonyl)-3,5-difluorophenyl-4'-pentyloxy-2'-methoxybenzoate 2.3d

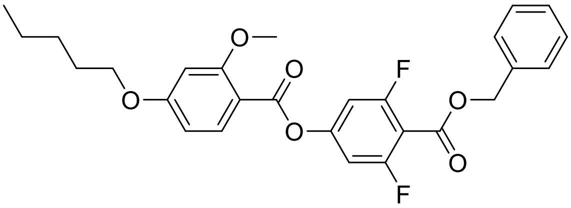

Method 2C

Benzoic acid 2.2d	514 mg,	2.1 mmol,	1 eq.
Benzyl-4-hydroxy-2,6-difluorobenzoate	870 mg,	2.1 mmol,	1 eq.
EDC.HCl	1.495 g,	3.2 mmol,	1.5 eq.
DMAP	15 mg,	0.1 mmol,	0.05 eq.
DCM	10 ml		

26 hrs
Yield 508 mg.
^{1}H NMR (400 MHz, $CDCl_3$) δ = 8.00 (d, J=8.7, 1H), 7.48 – 7.30 (m, 5H), 6.94 – 6.87 (m, 2H), 6.58 – 6.45 (overlapping signals, 2H), 5.40 (s, 2H), 4.04 (t, J=6.5, 2H), 3.92 (s, 2H), 1.82 (p, J=6.8, 2H), 1.52 – 1.35 (m, 4H), 0.99 – 0.91 (m, 3H).

4-((benzyloxy)carbonyl)-3,5-difluorophenyl-4’-hexyloxy-2’-methoxybenzoate 2.3e

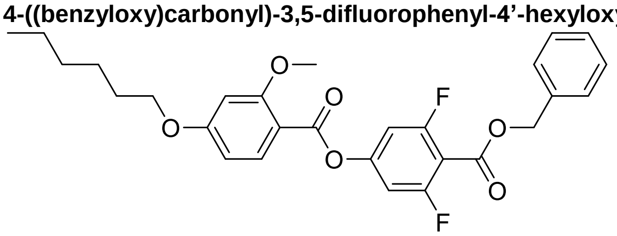

Method 2C

Benzoic acid 2.2e	627 mg,	2.5 mmol,	1.1 eq.
Benzyl-4-hydroxy-2,6-difluorobenzoate	608mg,	2.3 mmol,	1 eq.
EDC.HCl	887 mg,	4.5 mmol,	2 eq.
DMAP	20 mg,	0.2 mmol,	0.1 eq.
DCM	20 ml		

15 hrs
Colum chromatography: R_F (DCM) 0.44.
Yield 250 mg.
^{1}H NMR (400 MHz, $CDCl_3$) δ = 8.00 (d, *J*=8.7, 1H), 7.49 – 7.30 (m, 5H), 6.95 – 6.86 (m, 2H), 6.58 – 6.49 (overlapping signals, 2H), 5.40 (s, 2H), 4.04 (t, *J*=6.6, 2H), 3.92 (s, 3H), 1.81 (p, *J*=6.7, 2H), 1.50 – 1.45 (m, 2H), 1.40 – 1.31 (m, 4H), 1.01 – 0.86 (m, 3H).

4-((benzyloxy)carbonyl)-3,5-difluorophenyl-4’-heptyloxy-2’-methoxybenzoate 2.3f

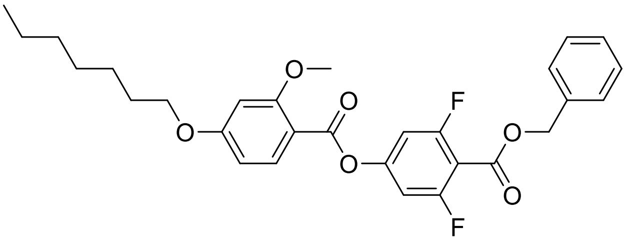

Method 2C

Benzoic acid 2.2f	664 mg,	2.5 mmol,	1.1 eq.
Benzyl-4-hydroxy-2,6-difluorobenzoate	610 mg,	2.3 mmol,	1 eq.
EDC.HCl	903 mg,	4.6 mmol,	2 eq.
DMAP	10 mg,	0.2 mmol,	0.04 eq.
DCM	15 ml		

15 hrs
Colum chromatography: R_F (DCM) 0.5.
Yield 206 mg.
^{1}H NMR (400 MHz, $CDCl_3$) δ = 8.00 (d, *J*=9.0, 1H), 7.48 – 7.30 (m, 5H), 6.91 (d, J_{HF}=9.7, 2H), 6.52 (overlapping signals, 2H), 5.40 (s, 2H), 4.03 (t, *J*=6.1, 2H), 3.92 (s, 3H), 1.85 – 1.77 (m, 2H), 1.50 – 1.44 (m, 2H), 1.40 – 1.30 (m, 6H), 0.92 – 0.88 (m, 3H).

4-carboxyl-3,5-difluorophenyl-4’-ethoxy-2’-methoxybenzoate 2.4a

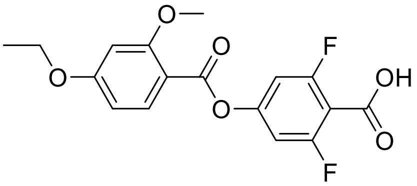

Method 2D

Benzyl ester 2.3a	165 mg,	0.4 mmol,	1 eq.
Triethylsilane	0.2 ml,	1.3 mmol,	3 eq.
5 % Pd/C	42mg,		
Ethanol	3 ml		
DCM	3 ml		

Yield 114 mg.
^{1}H NMR (400 MHz, $CDCl_3$) δ = 8.02 (d, *J*=8.5, 1H), 6.96 (d, J_{HF}=9.9, 2H), 6.58 – 6.51 (overlapping signals, 2H), 4.13 (q, *J*=6.9, 2H), 3.93 (s, 3H), 1.46 (t, *J*=6.9, 3H).

4-carboxyl-3,5-difluorophenyl-4'-propyloxy-2'-methoxybenzoate 2.4b

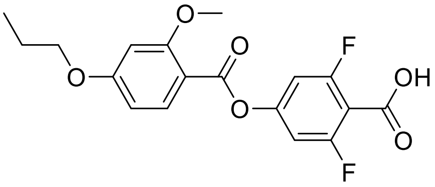

Method 2D

Benzyl ester 2.3b	158 mg,	0.4 mmol,	1 eq.
Triethylsilane	0.2 ml,	1.3 mmol,	3 eq.
5 % Pd/C	47 mg,		
Ethanol	3 ml		
DCM	3 ml		

Yield 101 mg.
^{1}H NMR (400 MHz, $CDCl_3$) δ = 8.01 (d, J=8.7, 1H), 6.96 (d, J_{HF}=9.9, 2H), 6.59 – 6.51 (overlapping signals, 2H), 4.01 (t, J=6.5, 2H), 3.93 (s, 3H), 1.86 (t, J=7.1, 2H), 1.07 (t, J=7.1, 3H).

4-carboxyl-3,5-difluorophenyl-4'-butyloxy-2'-methoxybenzoate 2.4c

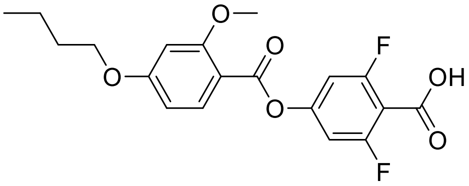

Method 2D

Benzyl ester 2.3c	154 mg,	0.3 mmol,	1 eq.
Triethylsilane	0.2 ml,	1.3 mmol,	3 eq.
5 % Pd/C	42 mg,		
Ethanol	3 mL		
DCM	3 ml		

Yield 97 mg.
^{1}H NMR (400 MHz, $CDCl_3$) δ = 8.01 (d, J=8.6, 1H), 6.95 (d, J_{HF}=9.7, 2H), 6.55 – 6.50 (overlapping signals, 1H), 4.05 (t, J=6.5, 2H), 3.93 (s, 3H), 1.83 – 1.77 (m, 2H), 1.55 – 1.49 (m, 2H), 1.00 (t, J=7.4, 3H).

4-carboxyl-3,5-difluorophenyl-4'-pentyloxy-2'-methoxybenzoate 2.4d

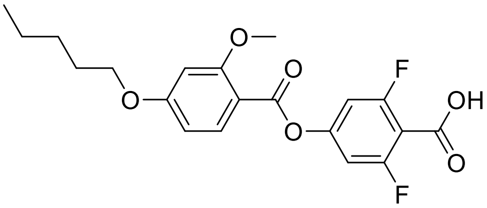

Method 2D

Benzyl ester 2.3d	500 mg,	1 mmol,	1 eq.
Triethylsilane	0.55 ml,	3.6 mmol,	3 eq.
5 % Pd/C	116 mg,		
Ethanol	6 ml		
DCM	6 ml		

Yield 377 mg.
^{1}H NMR (400 MHz, $CDCl_3$) δ = 8.01 (d, J=9.1, 1H), 6.99 – 6.92 (m, 2H), 6.58 – 6.50 (overlapping signals, 2H), 4.05 (t, J=6.5, 2H), 3.93 (s, 3H), 1.83 (p, J=6.7, 2H), 1.47 – 1.36 (m, 4H), 0.99 – 0.91 (m, 3H).

4-carboxyl-3,5-difluorophenyl-4'-hexyloxy-2'-methoxybenzoate 2.4e

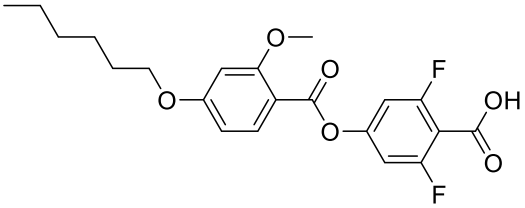

Method 2D

Benzyl ester 2.3e	230 mg,	0.5 mmol,	1 eq.
Triethylsilane	0.22 ml,	1.4 mmol,	3 eq.
5 % Pd/C	45 mg,		

Ethanol	5 ml		
DCM	5 ml		

Yield 170 mg.

^{1}H NMR (400 MHz, $CDCl_3$) δ = 8.03 (d, J=8.8, 1H), 7.28 (s, 2H), 6.97 (d, J_{HF}=9.0, 1H), 6.60 – 6.52 (overlapping signals, 2H), 4.06 (t, J=6.6, 2H), 3.95 (s, 3H), 1.84 (p, J=6.7, 2H), 1.57 – 1.44 (m, 2H), 1.41 – 1.34 (m, 4H), 0.98 – 0.90 (m, 3H).

4-carboxyl-3,5-difluorophenyl-4'-hexyloxy-2'-methoxybenzoate 2.4f

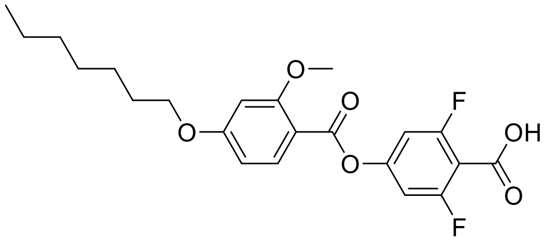

Method 2D

Benzyl ester 2.3f	230 mg,	0.5 mmol,	1 eq.
Triethylsilane	0.22 ml,	1.4 mmol,	3 eq.
5 % Pd/C	45 mg,		
Ethanol	5 ml		
DCM	5 ml		

Yield 170 mg.

^{1}H NMR (400 MHz, $CDCl_3$) δ = 8.04 (d, J=8.7, 1H), 6.98 (d, J_{HF}=9.0, 1H), 6.61 – 6.52 (overlapping signals, 2H), 4.07 (t, J=6.6, 2H), 3.95 (s, 3H), 1.90 – 1.79 (m, 2H), 1.55 – 1.41 (m, 2H), 1.44 – 1.30 (m, 6H), 0.96 – 0.89 (m, 3H).

Ester GS-2-Re

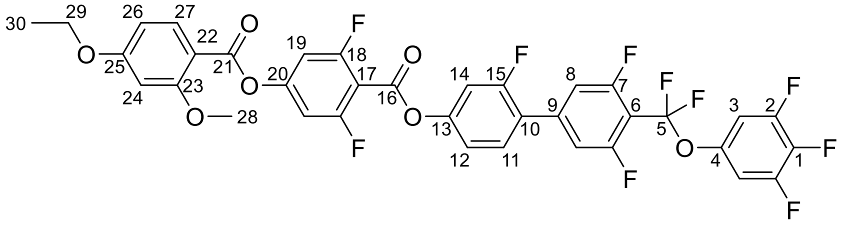

Method 2C

Benzoic acid 2.4a	57 mg,	0.16 mmol,	1.1 eq.
Phenol 2.6	61 mg,	0.14 mmol,	1 eq.
EDC.HCl	44 mg,	0.22 mmol,	1.5 eq.
DMAP	2 mg,	0.016 mmol,	0.1 eq.
DCM	10 ml		

13 hrs

Yield 27 mg.

m.p.105 °C, $T_{SmCF\text{-}NF}$ 66 °C, $T_{NF\text{-}N}$ 183 °C, $T_{N\text{-}I}$ 234 °C

^{1}H NMR (400 MHz, $CDCl_3$) δ = 8.04 (d, J=8.8, 1H, Ar-**H,** H27), 7.50 (t, $^3J_{HF}$=8.5, 1H, Ar-**H,** H14), 7.29 – 7.18 ($m_{overlapping}$, 4H, Ar-**H**, H18, H11, H13), 7.06 – 6.96 ($m_{overlapping}$, 4H, Ar-**H**, H3, H19), 6.61 – 6.52 ($m_{overlapping}$, 2H, Ar-**H**, H24, H26), 4.14 (q, J=7.0, 2H, O-**CH$_2$**-CH$_3$, H29), 3.94 (s, 3H, O-**CH$_3$**, H28), 1.47 (t, J=7.0, 3H, O-CH$_2$-**CH$_3$**, H30).

^{19}F NMR (376 MHz, $CDCl_3$) δ = -61.82 (t, $^4J_{FF}$=26.3, 2F, F5), -106.88 (d, $^3J_{HF}$=9.6, 2F, F18), -110.36 (td, $^4J_{FF}$=26.3, $^3J_{HF}$=10.6, 2F, F7), -113.63 (t, $^3J_{HF}$ =9.8, 1F, F15), -132.45 (dd, $^3J_{FF}$ =20.8, $^3J_{HF}$=8.0, 2F, F2), -163.11 (tt, $^3J_{FF}$ =20.8, 5.8, 1F, F1).

^{13}C NMR (101 MHz, $CDCl_3$) δ = 165.19 (1C), 162.78 (1C), 161.84 (1C), 161.67 (dd, $^1J_{CF}$=259.2, $^3J_{CF}$=7.3, 1C), 161.34 – 158.46 (m, 2C), 159.50 (d, $^1J_{CF}$=252.5, 2C), 158.93 (s, apparent t, 1C), 155.43 (t, $^3J_{CF}$=14.2, 1C), 151.62 (d, $^3J_{CF}$=11.0, 1C, 152.53 – 149.50 (m, 2C), 144.85 – 144.36 (m, 1C), 140.87 – 140.35 (m, 1C), 134.72 (1C), 130.61 (d, $^3J_{CF}$=3.9, 1C), 123.59 (d, $^3J_{CF}$=13.6, 1C), 120.22 – 119.97 (m, 2C), 118.35 (d, $^4J_{CF}$=3.7, 1C), 113.12 (dt, $^2J_{CF}$=25.0, $^4J_{CF}$=3.4, 2C), 110.84 (d, $^2J_{CF}$=25.8, 1C), 109.23 (1C), 107.47 (dd, $^2J_{CF}$=17.8, $^3J_{CF}$=7.3, 2C), 107.14 (dd, $^2J_{CF}$=25.4, $^4J_{CF}$=3.8, 2C), 106.62 – 106.32 (m, 1C), 105.51 (1C), 99.41 (1C), 64.05 (1C), 56.03 (1C), 14.63(1C).

HRMS (ESI): m/z calcd for $C_{36}H_{20}O_7F_{10}$ $[M+H]^+$: 755.11221. Found: 755.11225. Difference 0.052 ppm

IR ν_{max} (cm^{-1}): 3100 (C-H stretch), 2983 (C-H stretch, 1747 (C=O stretch, ester), 1709.

Ester GS-3-Re

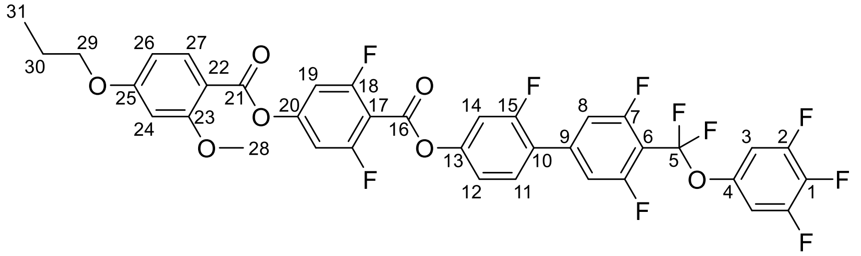

Method 2C

Benzoic acid 2.4b	50 mg,	0.15 mmol,	1.3 eq.
Phenol 2.6	51 mg,	0.12 mmol,	1 eq.
EDC.HCl	34 mg,	0.17 mmol,	1.4 eq.
DMAP	2 mg,	0.016 mmol,	0.1 eq.
DCM	10 ml		

13 hrs
Yield 14 mg.

m.p.82 °C, $T_{SmCF\text{-}NF}$ 90 °C, $T_{NF\text{-}N}$ 167 °C, $T_{N\text{-}I}$ 225 °C

^{1}H NMR (400 MHz, $CDCl_3$) δ = 8.03 (d, *J*=8.6, 1H Ar-**H,** H27), 7.50 (t, $^{3}J_{HF}$=8.6, 1H, Ar-**H,** H14), 7.26 – 7.17 ($m_{overlapping}$, 4H, Ar-**H**, H18, H11, H13), 7.06 – 6.96 ($m_{overlapping}$, 4H, Ar-**H**, H3, H19), 6.60 – 6.52 ($m_{overlapping}$, 2H, Ar-**H**, H24, H26), 4.02 (t, *J*=6.5, 2H, O-$\mathbf{CH_2}$-CH_2, H29), 3.94 (s, 2H, O-$\mathbf{CH_3}$, H28), 1.93 – 1.80 (m, 2H, $\mathbf{CH_2}$-CH_3, H30), 1.08 (t, *J*=7.4, 3H, CH_2-$\mathbf{CH_3}$, H31).

^{19}F NMR (376 MHz, $CDCl_3$) δ = -61.82 (t, $^{4}J_{FF}$ =26.3, 2F, F5), -106.90 (d, $^{3}J_{HF}$=9.8, 2F, F18), -110.37 (td, $^{4}J_{FF}$ =26.3, $^{3}J_{HF}$=6.3, 2F, F7), -113.63 (t, $^{3}J_{HF}$ =9.8, 1F, F15), -132.45 (dd, $^{3}J_{FF}$ =21.0, $^{3}J_{HF}$=8.4, 2F, F2), -163.12 (tt, $^{3}J_{FF}$ =21.0, 6.0, 1F, F1).

^{13}C NMR (101 MHz, $CDCl_3$) δ = 165.51 (1C), 162.89 (1C), , 163.56 – 159.99 (m, 1C), 161.94, (1C), 161.58 – 158.84 (m, 2C), 159.58 (d, $^{1}J_{CF}$=251.1, 2C), 158.75 (1C), 156.05 – 155.27 (m, 1C), 151.71 (d, $^{3}J_{CF}$=9.9, 1C), 152.76 – 149.43 (m, 2C), 145.03 – 144.30 (m, 1C), 140.76 (1C), 134.78 (1C), 130.69 (1C), 123.91 – 123.46 (m, 1C123.07 – 116.98 (m, 1C), 118.42 (1C), 113.92 – 112.71 (m, 2C), 110.93 (d, $^{2}J_{CF}$=26.1, 1C), 109.24 (1C), 107.76 – 107.38 (m, 2C), 107.41 – 106.66 (m, 2C106.65 – 106.48 (m, 1C), 105.69 (1C), 99.48 (1C), 70.07 (1C), 56.11 (1C), 22.53 (1C), 10.55 (1C).

HRMS (ESI): *m/z* calcd for $C_{37}H_{22}O_7F_{10}$ $[M+H]^+$: 769.12786. Found: 769.12802. Difference 0.207 ppm

IR ν_{max} (cm^{-1}): 3109 (C-H stretch), 2945 (C-H stretch), 2880 (C-H stretch), 1746 (C=O stretch, ester), 1709.

Ester GS-4-Re

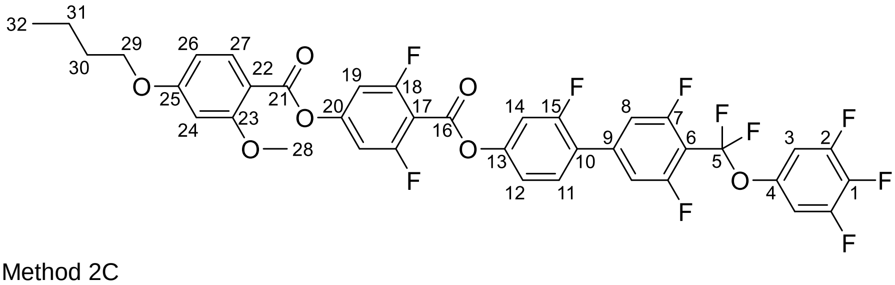

Method 2C

Benzoic acid 2.4c	168 mg,	0.49 mmol,	1.3 eq.
Phenol 2.6	152 mg,	0.36 mmol,	1 eq.
EDC.HCl	158 mg,	0.81 mmol,	2.3 eq.
DMAP	5 mg,	0.05 mmol,	0.1 eq.
DCM	10 ml		

7 hrs, purified by column chromatography (DCM) R_f 0.82, then recrystallized from EtOH
Yield 11 mg.

m.p. 61 °C, $T_{SmCF\text{-}NF}$ 90 °C, $T_{NF\text{-}N}$ 157 °C, $T_{N\text{-}I}$ 229 °C

^{1}H NMR (400 MHz, $CDCl_3$) δ = 8.03 (d, *J*=8.7, 1H Ar-**H,** H27), 7.50 (t, $^{3}J_{HF}$=8.6, 1H, Ar-**H,** H14), 7.29 – 7.19 ($m_{overlapping}$, 4H, Ar-**H**, H18, H11, H13), 7.05 – 6.96 ($m_{overlapping}$, 4H, Ar-**H**, H3, H19), 6.60 – 6.52 ($m_{overlapping}$, 2H, Ar-**H**, H24, H26), 4.06 (t, *J*=6.5, 2H, O-$\mathbf{CH_2}$-CH_2, H29), 3.95 (s, 2H, O-$\mathbf{CH_3}$, H28), 1.82 ($p_{apparent}$, *J*=7.0, 2H, O-CH_2-$\mathbf{CH_2}$, H30), 1.59 – 1.46 (m, 2H, $\mathbf{CH_2}$-CH_3, H31), 1.01 (t, *J*=7.4, 3H CH_2-$\mathbf{CH_3}$, H32).

^{19}F NMR (376 MHz, $CDCl_3$) δ = -61.81 (t, $^{4}J_{FF}$ =26.6, 2F, F5), -106.91 (d, $^{3}J_{HF}$=9.9, 2F, F18), -110.36 (td, $^{4}J_{FF}$ =26.5, $^{3}J_{HF}$=10.8, 2F, F7), -113.63 (t, $^{3}J_{HF}$ =9.6, 1F, F15), -132.45 (dd, $^{3}J_{FF}$ =20.9, $^{3}J_{HF}$=8.0, 2F, F2), -163.12 (tt, $^{3}J_{FF}$ =20.9, 6.0, 1F, F1).

^{13}C NMR (101 MHz, $CDCl_3$) δ = 165.57 (1C), 162.94 (1C), 162.01 (1C), 161.82 (dd, $^{1}J_{CF}$=259.0, $^{3}J_{CF}$=7.7, 1C), 160.07 (dd, $^{1}J_{CF}$=256.1, $^{3}J_{CF}$=4.7, 2C), 159.65 (d, $^{1}J_{CF}$=252.5, 2C), 159.08 (s, apparent t, 1C), 155.61 (t, $^{3}J_{CF}$=14.5, 1C), 151.77 (d, $^{3}J_{CF}$=11.1, 1C), 151.15 (ddd, $^{1}J_{CF}$ =251.4, $^{2}J_{CF}$=10.4, $^{3}J_{CF}$=5.3, 2C), 145.13 – 144.33 (m, 1C), 141.35 – 140.51 (m, 1C), 138.61 (dt, $^{1}J_{CF}$=251.4, $^{2}J_{CF}$=16.6, 1C), 134.84 (1C), 130.76 (d, $^{3}J_{CF}$=3.8, 1C), 123.74 (d, $^{3}J_{CF}$=12.7, 1C), 120.26 (t, $^{1}J_{CF}$=272.1, 1C), 118.49 (d, $^{4}J_{CF}$=3.8, 1C), 113.27 (dt, $^{2}J_{CF}$=24.7, $^{4}J_{CF}$=3.4, 2C), 112.70 – 112.09 (m, 1C), 110.98 (d, $^{2}J_{CF}$=25.9, 1C), 109.29 (1C), 107.61 (dd, $^{2}J_{CF}$=17.8, $^{3}J_{CF}$=6.5, 2C), 107.28 (dd, $^{2}J_{CF}$=25.4, $^{4}J_{CF}$=3.9, 2C), 106.61 (t, $^{2}J_{CF}$=16.6, 1C), 105.75 (1C), 99.53 (1C), 68.36 (1C), 56.17 (1C), 31.24 (1C), 19.32 (1C), 13.94 (1C).

HRMS (ESI): *m/z* calcd for $C_{38}H_{24}O_7F_{10}$ $[M+H]^+$: 783.14351. Found: 783.14383. Difference 0.407 ppm

IR ν_{max} (cm^{-1}): 3110 (C-H stretch), 2945 (C-H stretch), 2880 (C-H stretch), 1746 (C=O stretch, ester), 1709.

Ester GS-5-Re

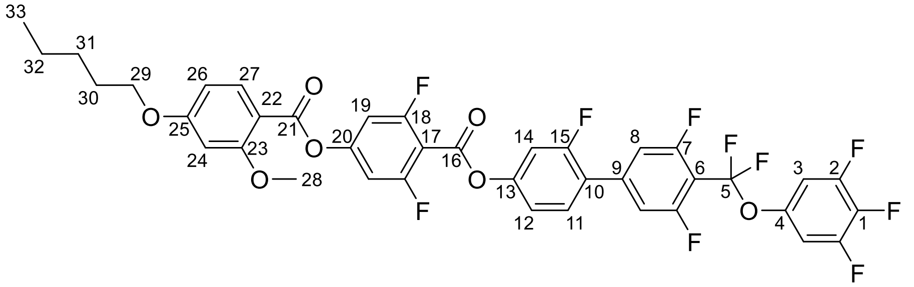

Method 2C			
Benzoic acid 2.4d	88 mg,	0.25 mmol,	1.3 eq.
Phenol 2.6	85 mg,	0.2 mmol,	1 eq.
EDC.HCl	62 mg,	0.32 mmol,	1.5 eq.
DMAP	3 mg,	0.02 mmol,	0.2 eq.
DCM	10 ml		
12 hrs			
Yield 27 mg.			

m.p. 73 °C, $T_{SmCF-NF}$ 91 °C, $T_{NF-SmAP}$ 126 °C $T_{SmAP-SmA}$ 129 °C T_{SmA-N} 136 °C, T_{N-I} 209 °C

^{1}H NMR (400 MHz, $CDCl_3$) δ = 8.03 (d, *J*=8.6, 1H Ar-**H,** H27), 7.50 (t, $^{3}J_{HF}$=8.5, 1H, Ar-**H,** H14), 7.25 – 7.18 ($m_{overlapping}$, 4H, Ar-**H**, H18, H11, H13), 7.06 – 6.96 ($m_{overlapping}$, 4H, Ar-**H**, H3, H19), 6.60 – 6.52 ($m_{overlapping}$, 2H, Ar-**H**, H24, H26), 4.06 (t, *J*=6.6, 2H, O-$\mathbf{CH_2}$-CH_2, H29), 3.94 (s, 2H, O-$\mathbf{CH_3}$, H28), 1.84 ($p_{apparent}$, *J*=6.6, 2H, O-CH_2-$\mathbf{CH_2}$, H30), 1.53 – 1.36 (m, 4H, $\mathbf{CH_2}$-$\mathbf{CH_2}$-CH_3, H31, H32), 0.96 (t, *J*=7.0, 3H CH_2-$\mathbf{CH_3}$, H33).

^{19}F NMR (376 MHz, $CDCl_3$) δ = -61.82 (t, $^{4}J_{FF}$ =26.3, 2F, F5), -106.90 (d, $^{3}J_{HF}$=9.8, 2F, F18), -110.36 (td, $^{4}J_{FF}$ =26.3, $^{3}J_{HF}$=10.6, 2F, F7), -113.63 (t, $^{3}J_{HF}$ =9.8, 1F, F15), -132.45 (dd, $^{3}J_{FF}$ =20.8, $^{3}J_{HF}$=8.1, 2F, F2), -163.12 (tt, $^{3}J_{FF}$ =20.8, 5.7, 1F, F1).

^{13}C NMR (101 MHz, $CDCl_3$) δ = 165.41 (1C), 162.94 (1C), 161.85 (1C), 161.67 (dd, $^{1}J_{CF}$=258.9, $^{3}J_{CF}$=7.7, 1C), 160.85 – 157.89 (m, 2C), 161.31 – 158.52 (m, 2C), 158.94 (s, apparent t, 1C), 155.76 – 155.22 (m, 1C), 151.62 (d, $^{3}J_{CF}$=11.1, 1C), 151.00 (ddd, $^{1}J_{CF}$ =251.4, $^{2}J_{CF}$=10.8, $^{3}J_{CF}$=5.6, 2C), 144.72 – 144.30 (m, 1C), 140.89 – 140.45 (m, 1C), 139.94 – 136.89 (m, 1C), 134.70 (1C), 130.61 (d, $^{3}J_{CF}$=3.8, 1C), 123.64 (d, $^{3}J_{CF}$=12.1, 1C), 120.11 (t, $^{1}J_{CF}$=266.3, 1C), 118.35 (d, $^{4}J_{CF}$=3.9, 1C), 113.13 (dt, $^{2}J_{CF}$=23.5, $^{4}J_{CF}$=3.1, 2C), 110.84 (d, $^{2}J_{CF}$=25.8, 1C), 109.14 (1C), 107.75 – 107.26 (m, 2C), 107.14 (dd, $^{2}J_{CF}$=25.3, $^{4}J_{CF}$=3.9, 2C), 106.47 (t, $^{2}J_{CF}$=16.3, 1C), 105.58(1C), 99.38(1C), 68.51 (1C), 56.03 (1C), 28.77 (1C), 28.11 (1C), 22.42 (1C), 14.00 (1C).

HRMS (ESI): *m/z* calcd for $C_{39}H_{26}O_7F_{10}$ $[M+H]^+$: 797.15916. Found: 797.15946. Difference 0.375 ppm

IR ν_{max} (cm^{-1}): 3099 (C-H stretch), 2939 (C-H stretch), 2875 (C-H stretch), 1734 (C=O stretch, ester).

Ester GS-6-Re

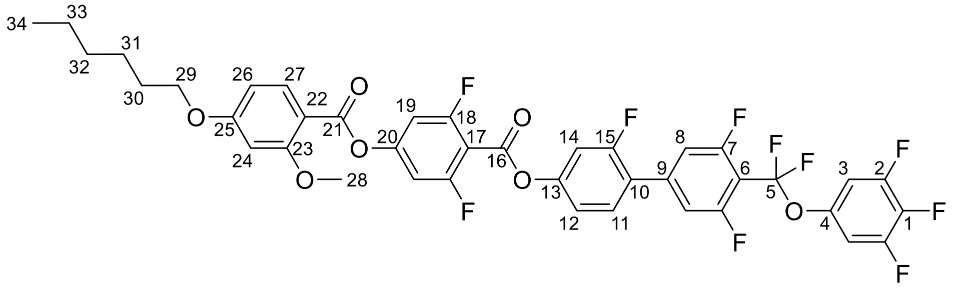

Method 2C

Benzoic acid 2.4e	122 mg,	0.3 mmol,	1.1 eq.
Phenol 2.6	112 mg,	0.27 mmol,	1 eq.
EDC.HCl	113 mg,	0.58 mmol,	2 eq.
DMAP	3 mg,	0.02 mmol,	0.1 eq.
DCM	10 ml		

14 hrs, purified by column chromatography (2:1 DCM:Hexane) then recrystallized from EtOH. R_f (DCM) 0.5. Yield 37 mg.

m.p. 85 °C, $T_{SmCF\text{-}SmA}$ 81 °C, $T_{SmA\text{-}N}$ 152 °C, $T_{N\text{-}I}$ 204 °C

^{1}H NMR (400 MHz, $CDCl_3$) δ = 8.02 (d, *J*=8.7, 1H Ar-**H,** H27), 7.50 (t, $^3J_{HF}$=8.9, 1H, Ar-**H,** H14), 7.26 – 7.17 ($m_{overlapping}$, 4H, Ar-**H**, H18, H11, H13), 7.06 – 6.96 ($m_{overlapping}$, 4H, Ar-**H**, H3, H19), 6.60 – 6.51 ($m_{overlapping}$, 2H, Ar-**H**, H24, H26), 4.05 (t, *J*=6.5, 2H, O-$\mathbf{CH_2}$-CH_2, H29), 3.94 (s, 2H, O-$\mathbf{CH_3}$, H28), 1.88 – 1.77 (m, 2H, O-CH_2-$\mathbf{CH_2}$, H30), 1.55 – 1.43 (m, 2H, O-CH_2-CH_2-$\mathbf{CH_2}$, H31, H32), 1.41 – 1.32 (m, 4H, $\mathbf{CH_2}$-$\mathbf{CH_2}$-CH_3, H32, H33) 0.97 – 0.88 (m, 3H CH_2-

^{19}F NMR (282 MHz, $CDCl_3$) δ = -61.78 (t, $^4J_{FF}$=26.6, 2F, F5), -106.88 (d, $^3J_{HF}$=9.2, 2F, F18), -110.33 (td, $^4J_{FF}$=26.7, $^3J_{HF}$=10.4, 2F, F7), -113.60 (t, $^3J_{HF}$=9.6, 1F, F15), -132.43 (dd, $^3J_{FF}$=20.9, $^3J_{HF}$=7.5, 2F, F2), -163.12 (tt, $^3J_{FF}$=20.9, 5.8, 1F, F1).

^{13}C NMR (101 MHz, $CDCl_3$) δ = 165.46 (1C), 162.83 (1C), 161.90 (1C), 161.72 (dd, $^1J_{CF}$=259.2, $^3J_{CF}$=7.5, 1C), 161.53 – 158.43 (m, 2C), 159.55 (d, $^1J_{CF}$=252.6, 2C), 158.97 (s, apparent t, 1C), 155.73 – 155.19 (m, 2C), 151.67 (d, $^3J_{CF}$=11.1, 1C), 152.57 – 149.59 (m, 2C), 144.96 – 144.39 m, 1C), 140.96 – 140.51 (m, 1C), 134.73 (1C), 130.65 (d, $^3J_{CF}$=3.9, 1C), 123.64 (d, $^3J_{CF}$=11.6, 1C), 118.39 (d, $^4J_{CF}$=4.0, 1C), 113.17 (dt, $^2J_{CF}$=23.7, $^4J_{CF}$=2.2, 2C), 110.88 (d, $^2J_{CF}$=25.8, 1C), 109.21 (1C), 107.76 – 107.30 (m, 2C), 107.18 (dd, $^2J_{CF}$=25.3, $^4J_{CF}$=3.9, 2C), 106.89 – 106.42 (m, 1C), 105.65 (1C), 99.44 (1C), 68.58 (1C), 56.08 (1C), 29.08 (1C), 25.69 (1C), 22.62 (1C), 14.06 (1C).

HRMS (ESI): *m*/*z* calcd for $C_{40}H_{28}O_7F_{10}$ $[M+H]^+$: 811.17481. Found: 811.17446. Difference -0.433 ppm

IR ν_{max} (cm^{-1}): 3101 (C-H stretch), 2940 (C-H stretch), 2873 (C-H stretch), 1738 (C=O stretch, ester).

Ester GS-7-Re

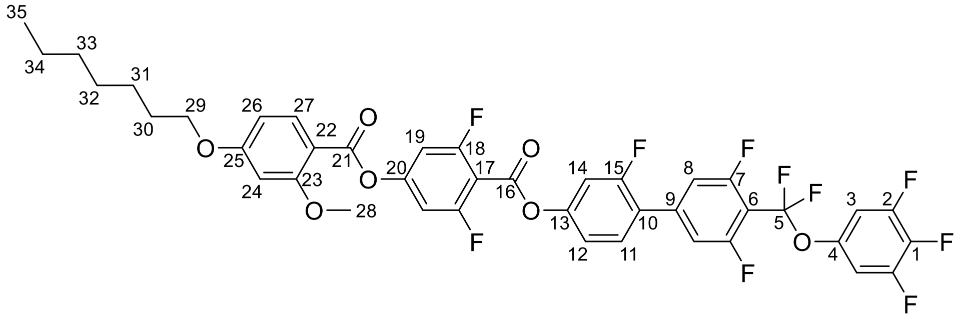

Method 2C

Benzoic acid 2.4f	104 mg,	0.25 mmol,	1.1 eq.
Phenol 2.6	95 mg,	0.23 mmol,	1 eq.
EDC.HCl	105 mg,	0.54 mmol,	2.3 eq.
DMAP	3 mg,	0.02 mmol,	0.1 eq.
DCM	15 ml		

14 hrs, purified by column chromatography (2:1 DCM:Hexane) then recrystallized from EtOH. R_F (DCM) 0.5. Yield 24 mg.
m.p. 73 °C, $T_{SmCF\text{-}SmA}$ 53 °C, $T_{SmA\text{-}N}$ 160 °C, $T_{N\text{-}I}$ 196 °C

^{1}H NMR (400 MHz, $CDCl_3$) δ = 8.03 (d, *J*=8.7, 1H Ar-**H,** H27), 7.50 (t, $^3J_{HF}$=8.6, 1H, Ar-**H,** H14), 7.29 – 7.17 ($m_{overlapping}$, 4H, Ar-**H**, H18, H11, H13), 7.06 – 6.96 ($m_{overlapping}$, 4H, Ar-**H**, H3, H19), 6.60 – 6.51 ($m_{overlapping}$, 2H, Ar-**H**, H24, H26), 4.05 (t, *J*=6.6, 2H, O-**CH_2**-CH_2, H29), 3.94 (s, 2H, O-**CH_3**, H28), 1.83 ($p_{apparent}$, *J*=6.8, 2H, O-CH_2-**CH_2**, H30), 1.48 ($p_{apparent}$, *J*=7.0, 2H, O-CH_2-CH_2-**CH_2**, H31, H32), 1.42 – 1.28 (m, 6H, **CH_2**-**CH_2**-**CH_2**-CH_3, H32, H33, H34) 0.95 – 0.87 (m, 3H CH_2-**CH_3**, H35).

^{19}F NMR (282 MHz, $CDCl_3$) δ = -61.78 (t, $^4J_{FF}$ =26.5, 2F, F5), -106.89 (d, $^3J_{HF}$=9.2, 2F, F18), -110.34 (td, $^4J_{FF}$ =26.5, $^3J_{HF}$=10.3, 2F, F7), -113.60 (t, $^3J_{HF}$ =10.0, 1F, F15), -132.44 (dd, $^3J_{FF}$ =20.9, $^3J_{HF}$=8.1, 2F, F2), -163.11 (tt, $^3J_{FF}$ =20.9, 5.8, 1F, F1).

^{13}C NMR (101 MHz, $CDCl_3$) δ = 165.55 (1C), 162.92 (1C), 162.08 – 161.88 (m, 1C), 161.80 (dd, $^1J_{CF}$=258.2, $^3J_{CF}$=8.8, 1C), 161.46 – 158.63 (m, 2C), 161.07 – 158.04 (m, 2C), 159.04 (s, apparent t, 1C155.78 – 155.33 (m, 1C), 151.73 (d, $^3J_{CF}$=7.4, 1C), 152.60 – 149.55 (m, 2C), 144.92 – 144.40 (m, 1C), 134.80 (1C), 130.91 – 130.46 (m, 2C), 123.70 (d, $^3J_{CF}$=15.9, 1C), 120.29 – 120.06 (m, 1C), 118.63 – 118.36 (m, 1C), 113.23 (m, 2C), 110.96 (d, $^2J_{CF}$=26.4, 1C), 109.30 (1C), 107.90 – 107.39 (m, 2C), 107.41 – 106.93 (m, 2C), 105.73 (1C), 99.52 (1C), 68.66 (1C), 56.16 (1C), 31.88 (1C), 29.21 (1C), 29.1 (1C), 26.06 (1C), 22.73 (1C), 14.20 (1C).

HRMS (ESI): *m/z* calcd for $C_{41}H_{30}O_7F_{10}$ $[M+H]^+$: 825.19046. Found: 825.18986. Difference -0.729 ppm

IR ν_{max} (cm^{-1}): 3113 (C-H stretch), 2928 (C-H stretch), 2857 (C-H stretch), 1747 (C=O stretch, ester), 1736.

NMR Spectra GS-*n*-Re

GS-2-Re

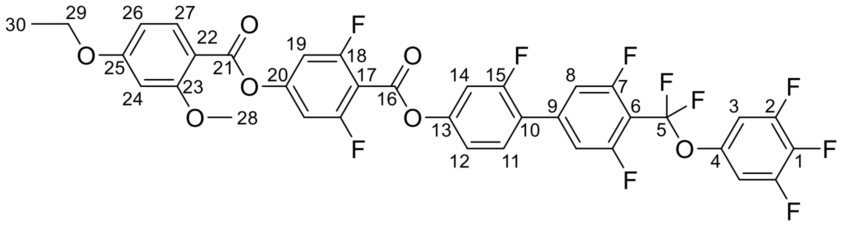

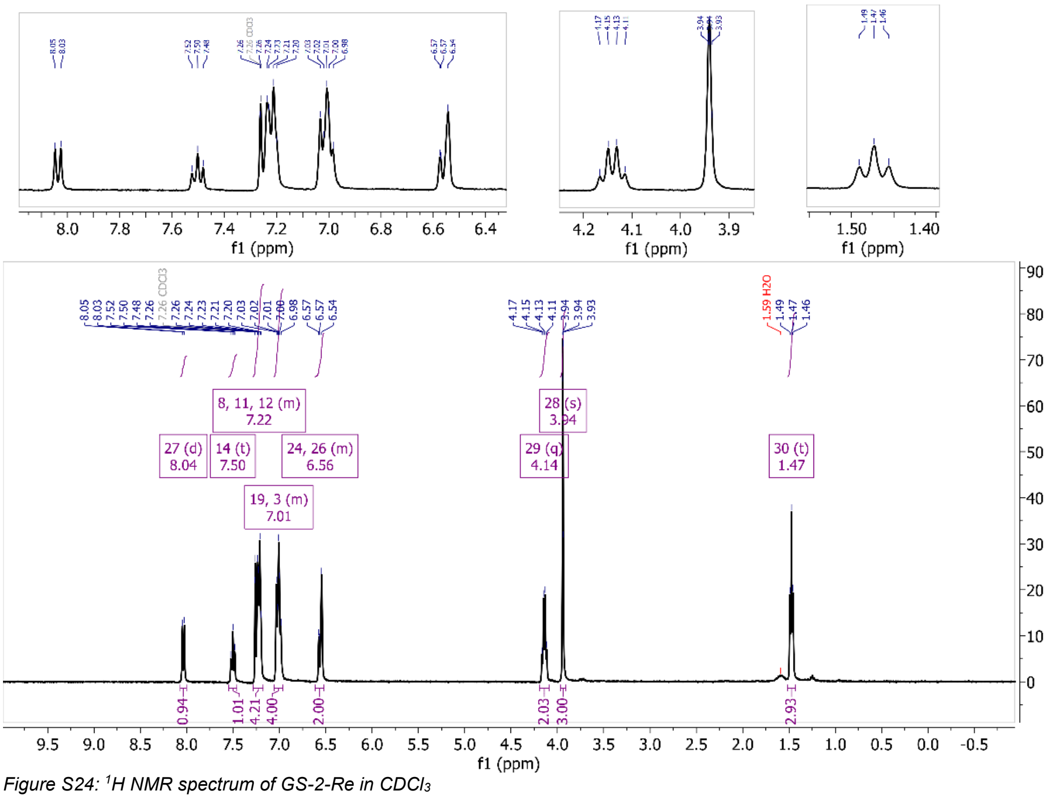

Figure S24: ^{1}H NMR spectrum of GS-2-Re in $CDCl_3$

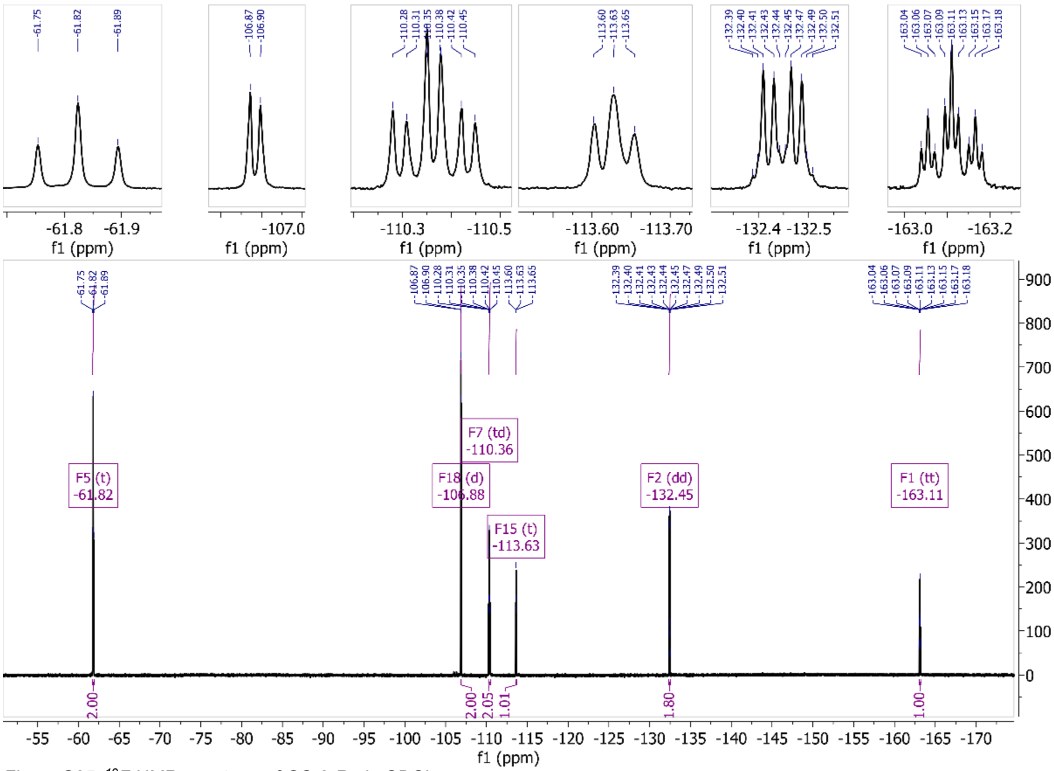

Figure S25: ^{19}F NMR spectrum of GS-2-Re in $CDCl_3$

Ester GS-3-Re

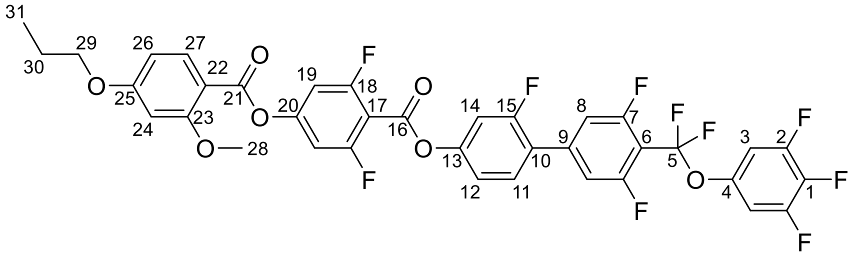

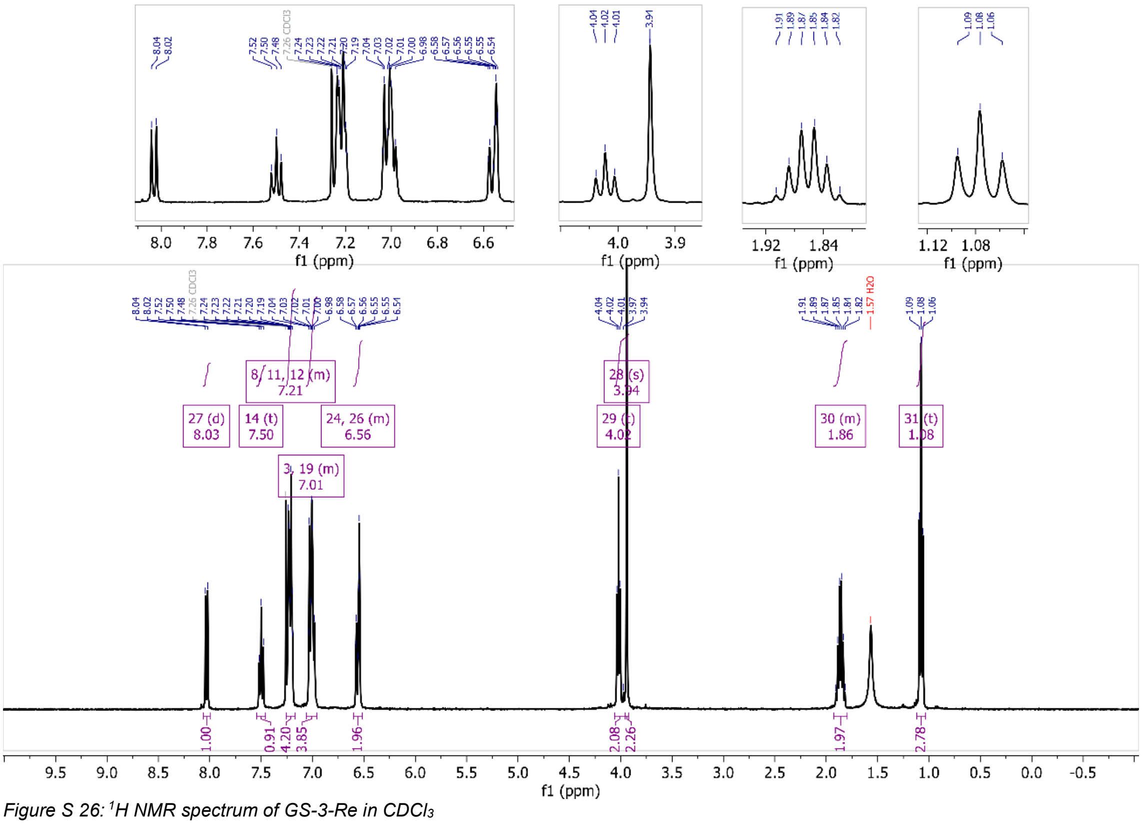

Figure S 26: ^{1}H NMR spectrum of GS-3-Re in $CDCl_3$

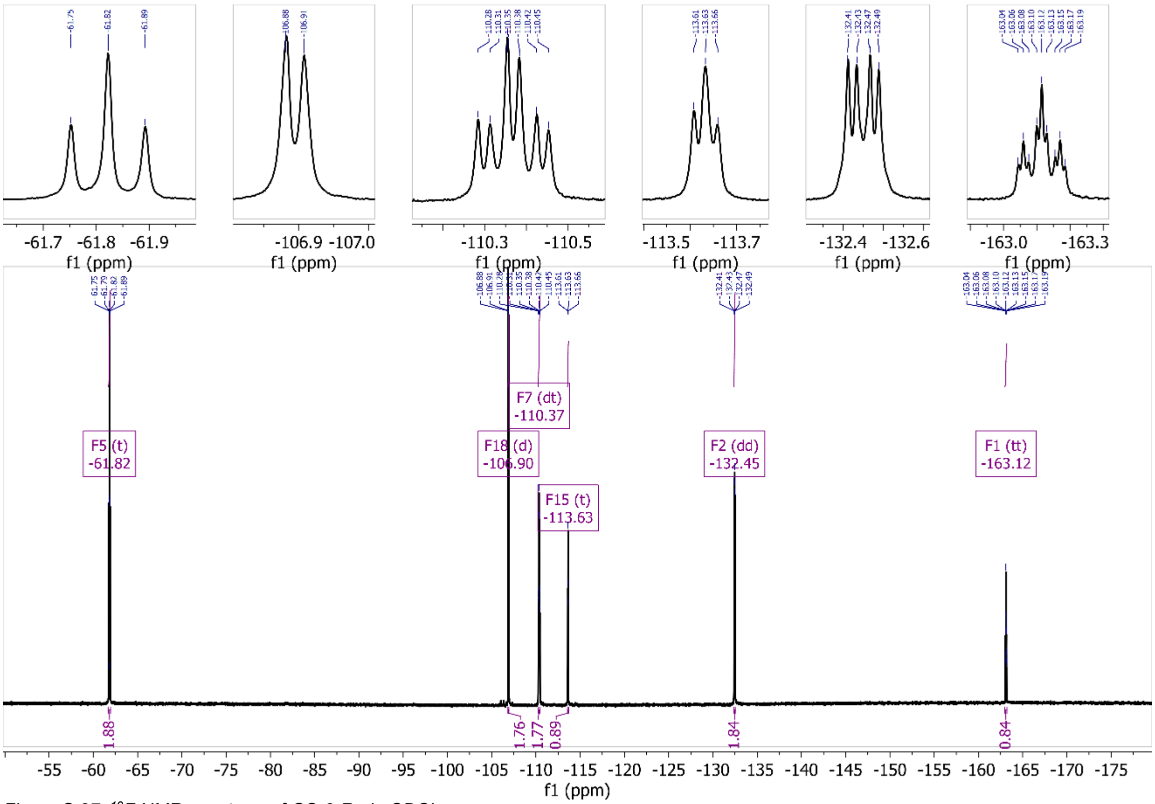

Figure S 27: ^{19}F NMR spectrum of GS-3-Re in $CDCl_3$

Ester GS-4-Re

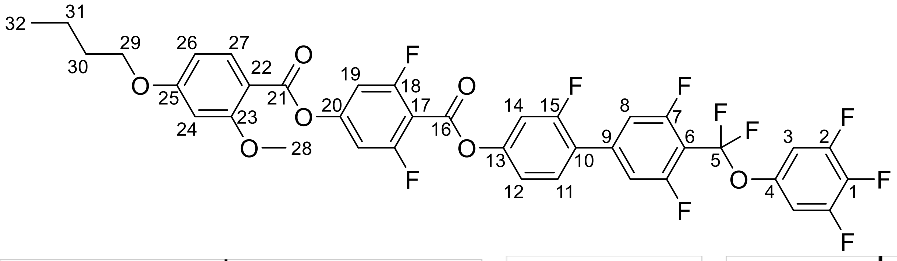

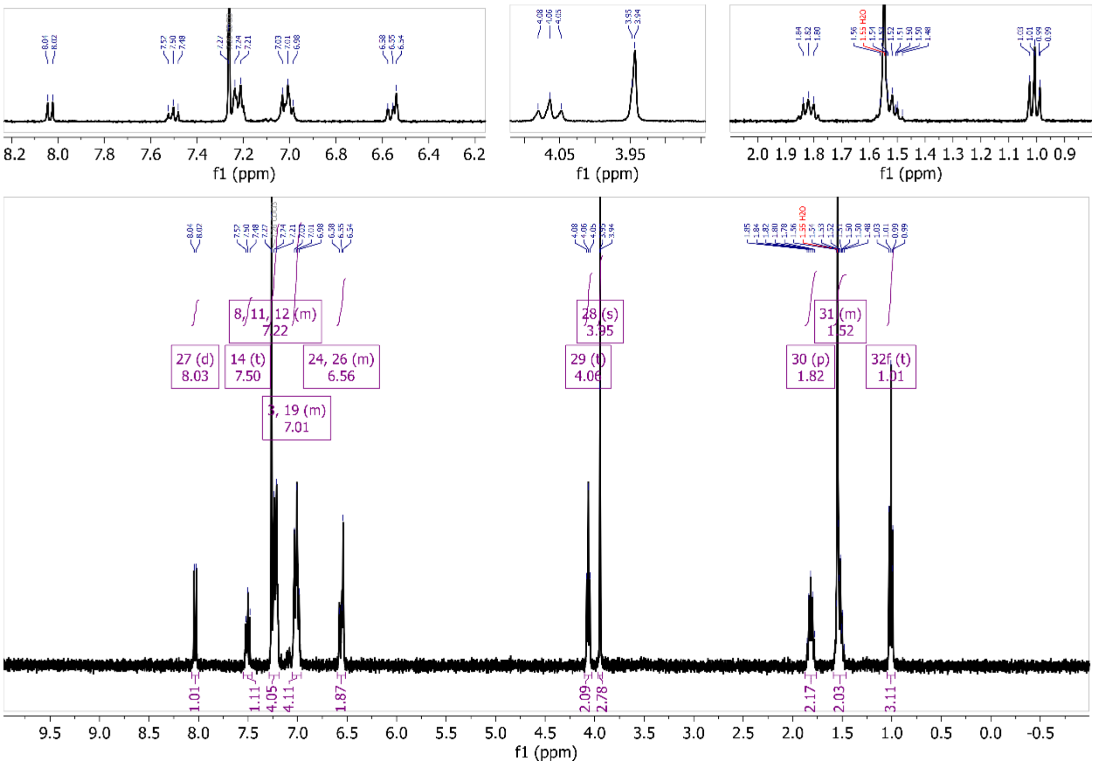

Figure S28: ^{1}H NMR spectrum of GS-4-Re in CDCl₃

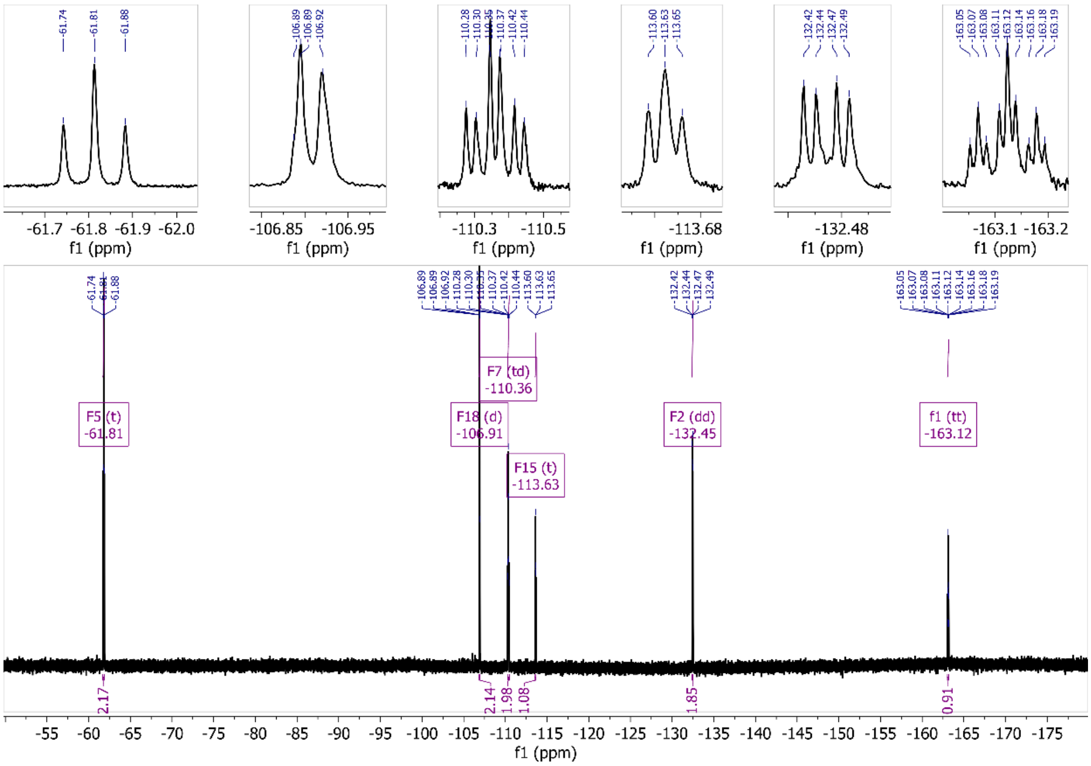

Figure S29: ^{19}F NMR spectrum of GS-4-Re in CDCl₃

Ester GS-5-Re

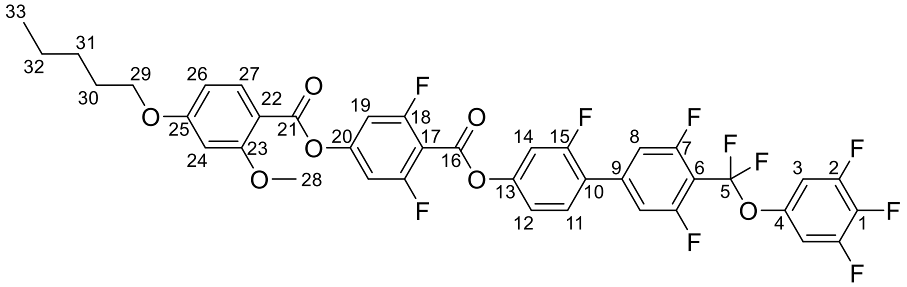

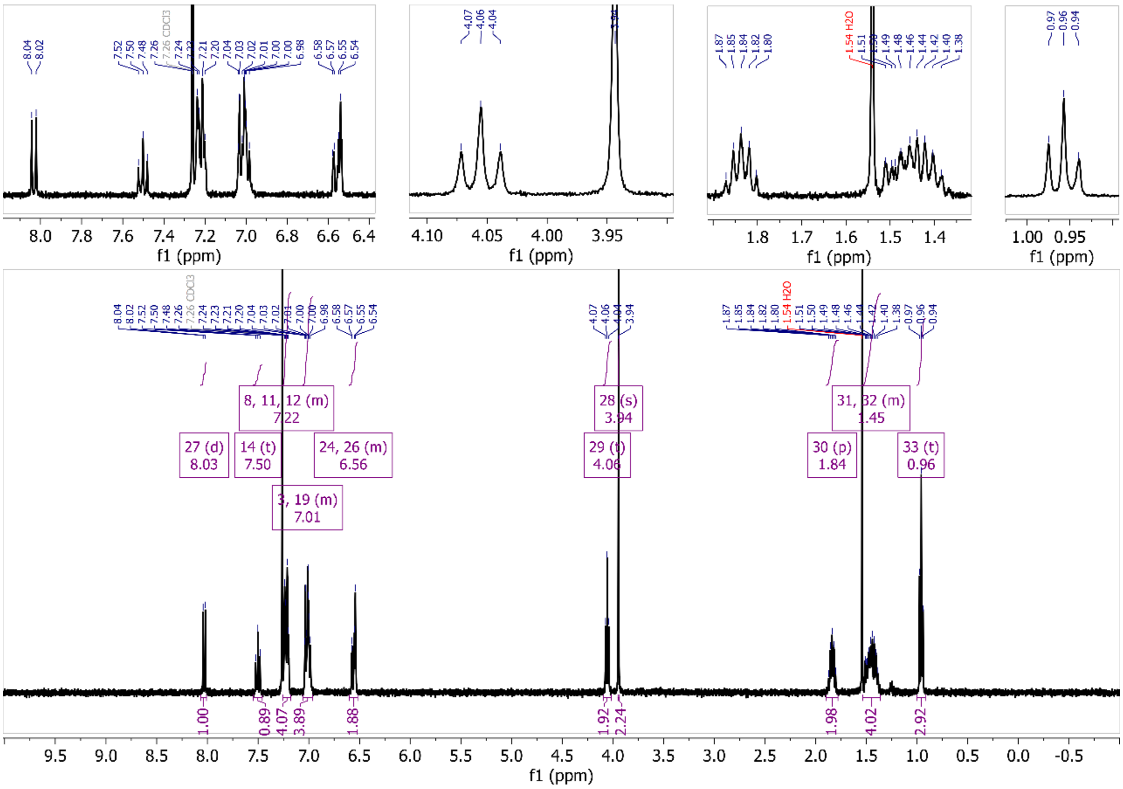

Figure S30: ¹H NMR spectrum of GS-5-Re in CDCl₃

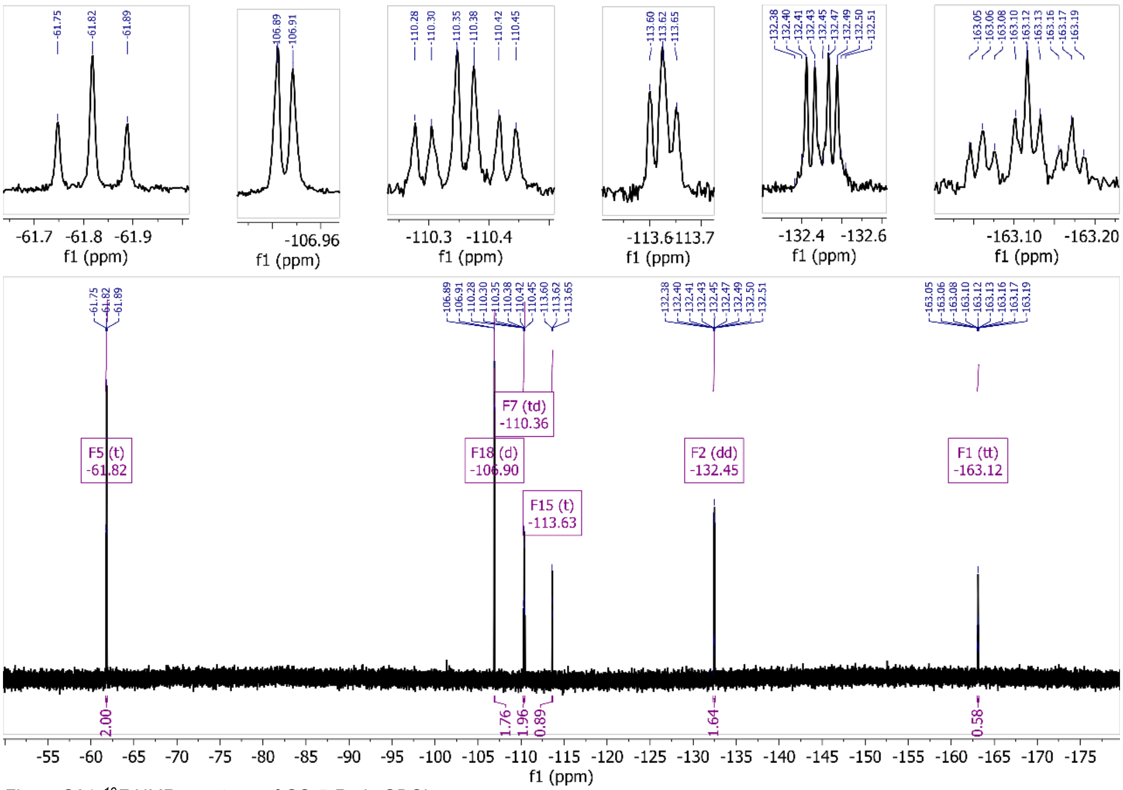

Figure S31: ¹⁹F NMR spectrum of GS-5-Re in CDCl₃

Ester GS-6-Re

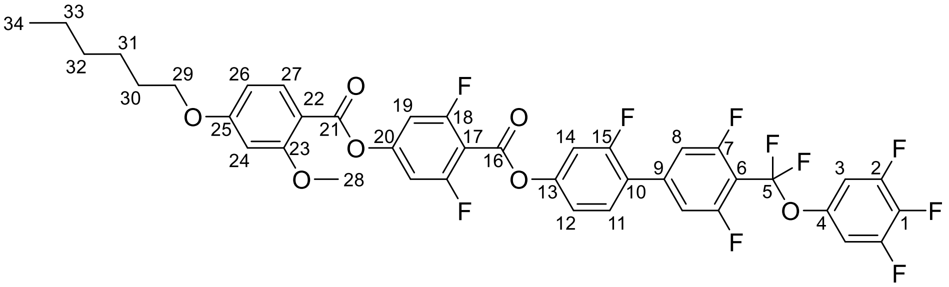

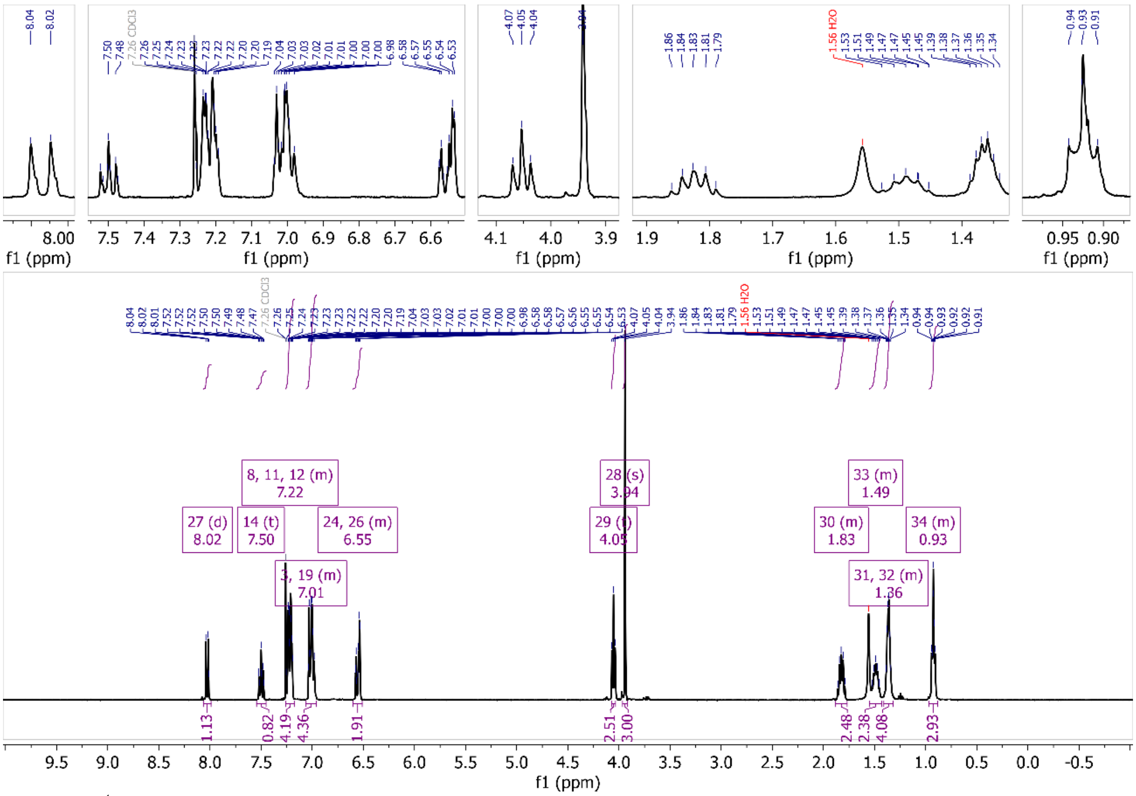

Figure S32: ¹H NMR spectrum of GS-6-Re in CDCl₃

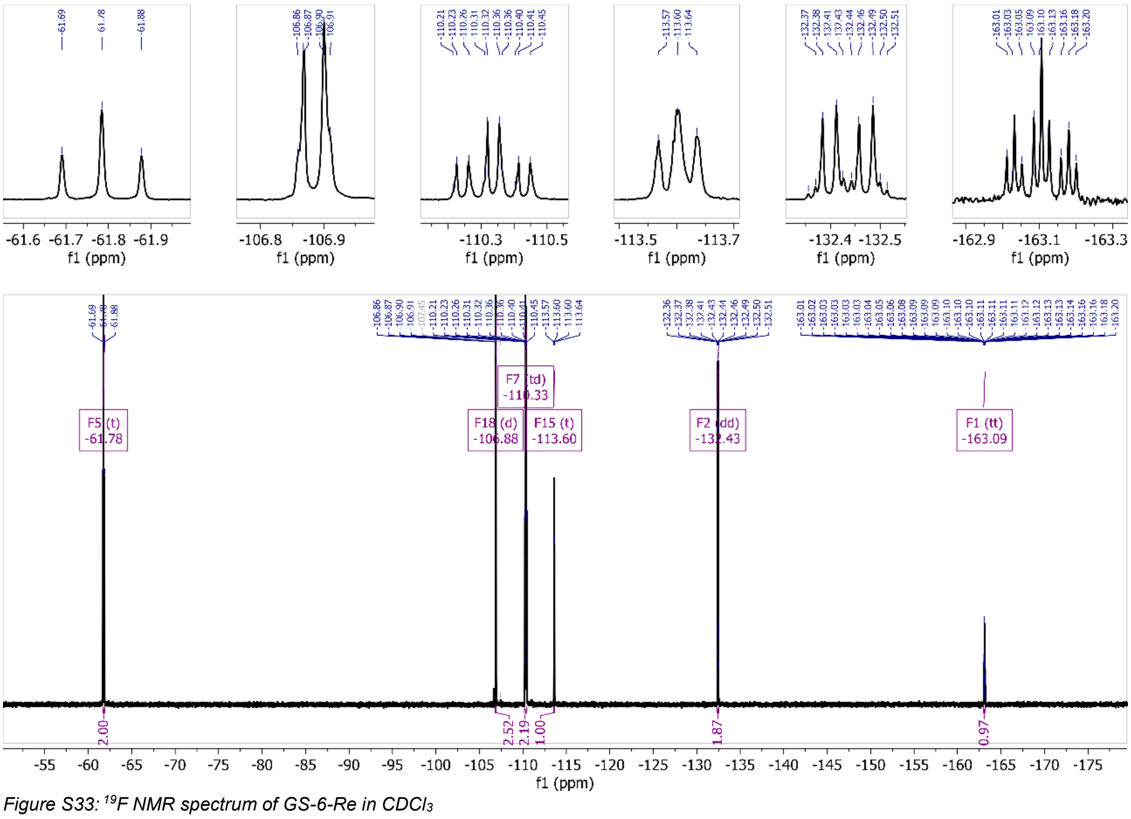

Figure S33: ¹⁹F NMR spectrum of GS-6-Re in CDCl₃

Ester GS-7-Re

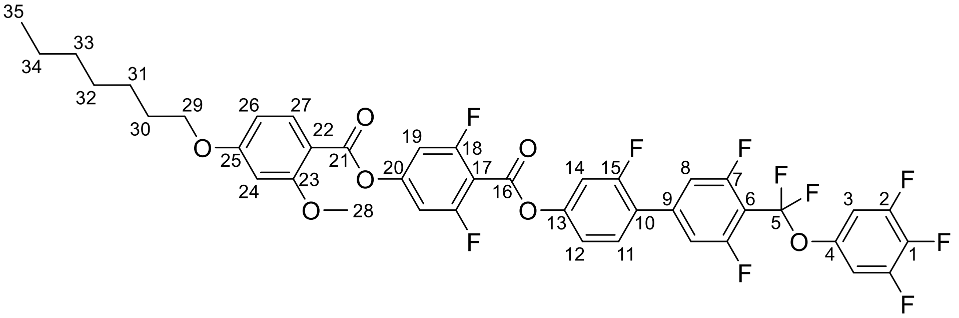

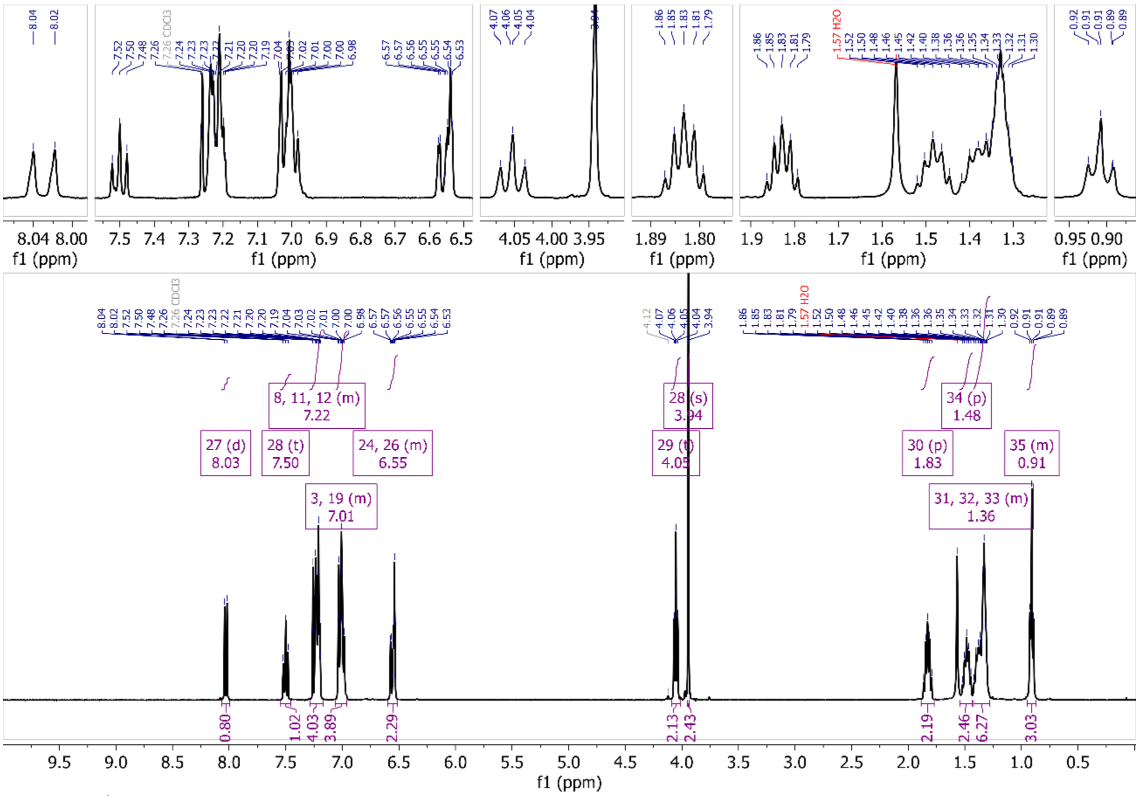

Figure S34: ^{1}H NMR spectrum of GS-7-Re in $CDCl_3$

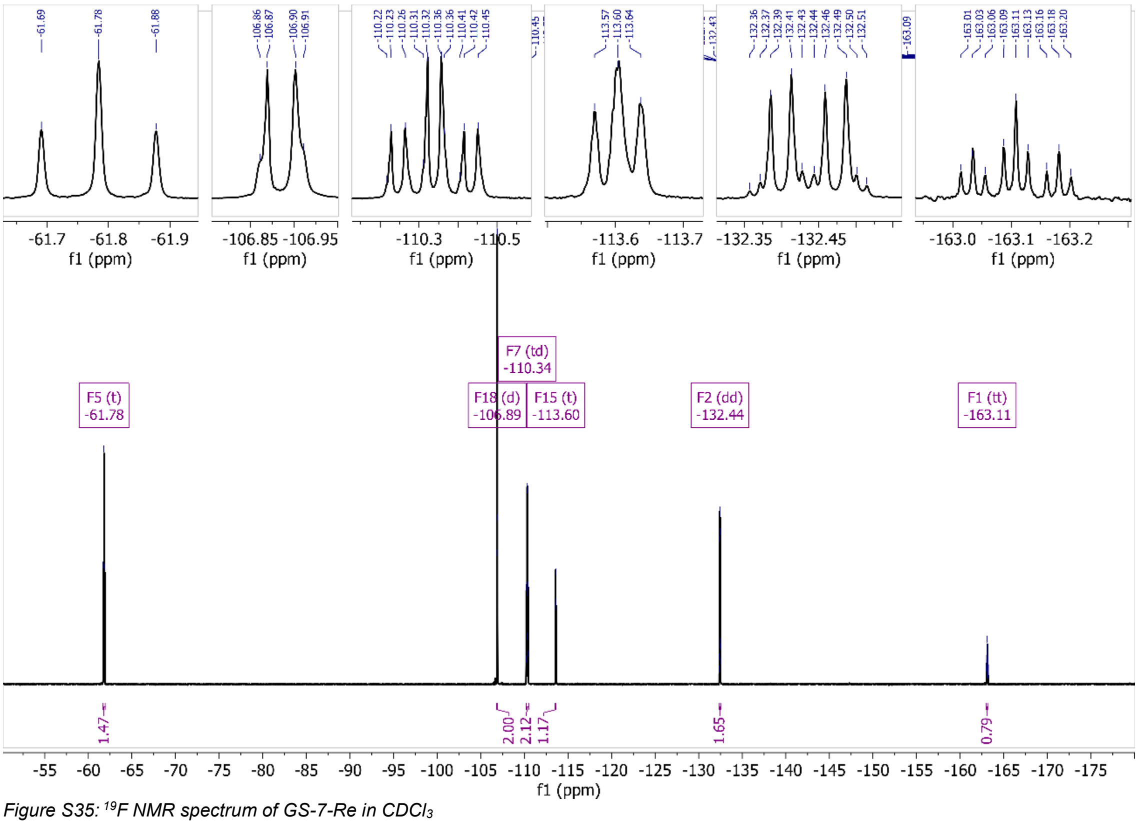

Figure S35: ^{19}F NMR spectrum of GS-7-Re in $CDCl_3$

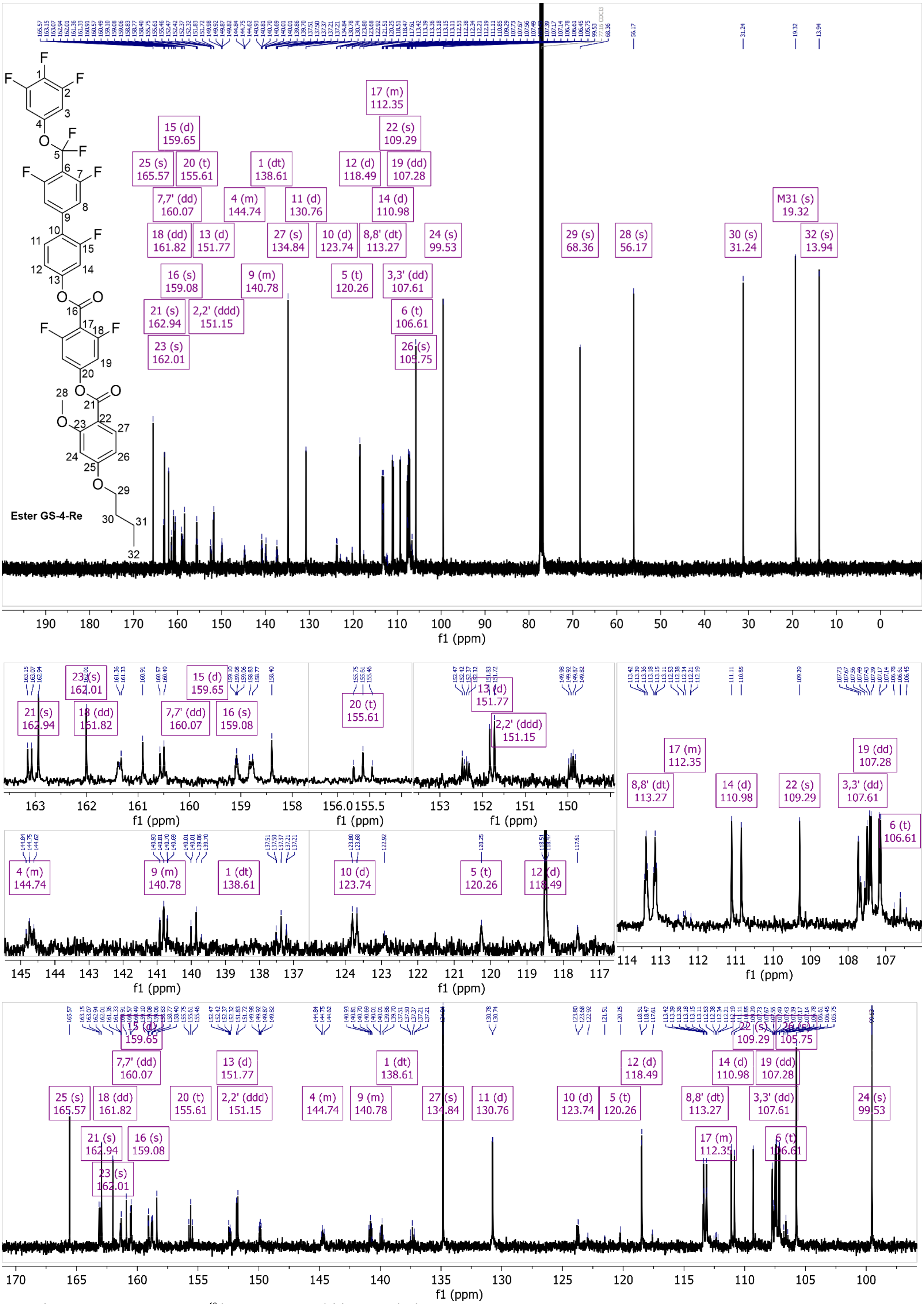

Figure S36: Representative assigned ^{13}C NMR spectrum of GS-4-Re in $CDCl_3$. Top: Full spectrum; bottom: enlarged aromatic region.

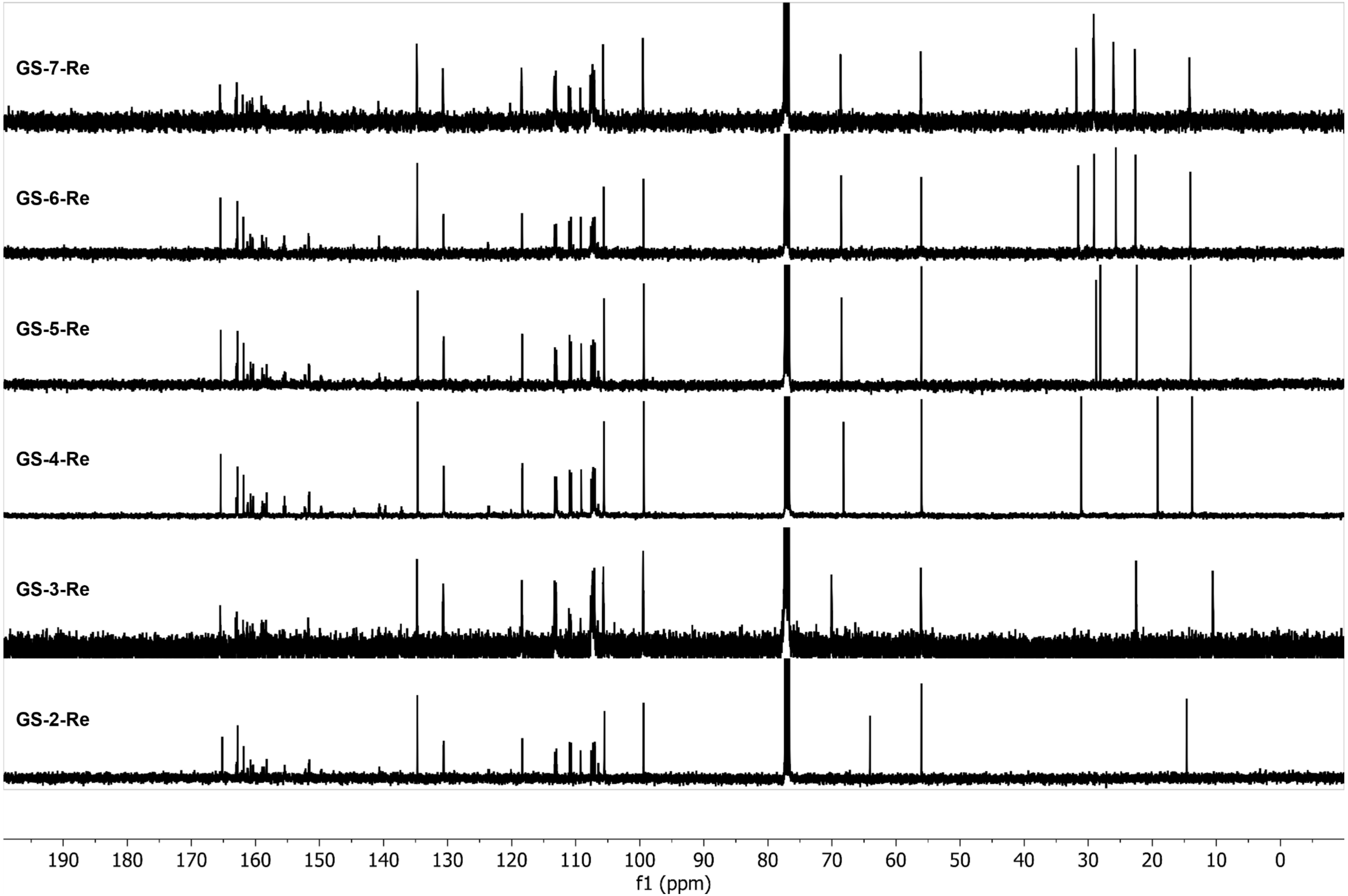

Figure S37: Stacked ^{13}C NMR spectra for series GS-n-Re.

4. Additional References

[28] M. Juodka, G. J. Strachan, S. Brown, D. Pociecha, E. Gorecka, J. M. D. Storey, C. T. Imrie, R. Walker, *Liquid Crystals* **2025**, 1–11. https://doi.org/10.1080/02678292.2025.2507807.

[29] D. Pociecha, J. Szydlowska, N. Vaupotič, K. Kwiatkowska, M. Juodka, J. Spiess, J. M. D. Storey, C. T. Imrie, R. Walker, E. Gorecka, *Adv. Sci.* **2025***, e08405.*.

[30] E. Cruickshank, N. Tufaha, R. Walker, S. Brown, E. Gorecka, D. Pociecha, J. M. D. Storey, C. T. Imrie, *Liquid Crystals* **2024**, *51*, 401–415.

[31] G. J. Strachan, E. Górecka, J. Hobbs, D. Pociecha, *J. Am. Chem. Soc.* **2025**, *147*, 6058–6066.
